# Absence of effects of widespread badger culling on tuberculosis in cattle

**DOI:** 10.1038/s41598-024-67160-0

**Published:** 2024-07-15

**Authors:** Paul R. Torgerson, Sonja Hartnack, Philip Rasmussen, Fraser Lewis, Thomas E. S. Langton

**Affiliations:** 1 Section of Veterinary Epidemiology, Vetsuisse Faculty, University of Zürich, Zürich, Switzerland; 2 London, UK; 3 Herpetofauna Consultants International, Suffolk, UK; 4 Department of Veterinary and Animal Sciences, University of Copenhagen, Copenhagen, Denmark

**Keywords:** Statistical methods, Diseases, Infectious diseases, Agroecology, Ecological epidemiology

## Abstract

Government policy in England aims for the elimination of bovine tuberculosis (bTB). This policy includes culling of European badger (*Meles meles*) to reduce cattle TB incidence. The rationale is based on a field trial, the Randomised Badger Culling Trial (RBCT) 1998–2005, which reported a substantial decrease in bTB herd incidence where badger culling had been implemented, in comparison to untreated control areas. The RBCT was undertaken because previous studies of reductions in badgers by culling, reported a possible association between bTB in badger and cattle, but none could directly show causation. The effect of intensive widespread (proactive) culling in the RBCT was reported in 2006 in the journal *Nature*. Analysis of an extensive badger removal programme in England since 2013 has raised concerns that culling has not reduced bTB herd incidence. The present study re-examined RBCT data using a range of statistical models. Most analytical options showed no evidence to support an effect of badger culling on bTB herd incidence ‘confirmed’ by visible lesions and/or bacterial culture *post mortem* following a comparative intradermal skin test (SICCT). However, the statistical model chosen by the RBCT study was one of the few models that showed an effect. Various criteria suggest that this was not an optimal model, compared to other analytical options available. The most likely explanation is that the RBCT proactive cull analysis over-fitted the data with a non-standard method to control for exposure giving it a poor predictive value. Fresh appraisal shows that there was insufficient evidence to conclude RBCT proactive badger culling affected bTB breakdown incidence. The RBCT found no evidence of an effect of culling on ‘total’ herd incidence rates. Total herd incidences include those confirmed as bTB at necropsy and those herds where there was at least one animal animal positive to the comparative intradermal skin test, the standard diagnostic test used for routine surveillance, but not confirmed at necropsy. This was also the case using the more suitable statistical models. Use only of ‘confirmed’ herd incidence data, together with a more recent (2013) published perception that RBCT data presented *‘a strong evidence base….with appropriate detailed statistical or other quantitative analysis’* should be reconsidered. The results of the present report are consistent with other analyses that were unable to detect any disease control benefits from badger culling in England (2013–2019). This study demonstrates one form of potential driver to the reproducibility crisis, in this case with disease control management in an increasingly intensified livestock industry.

## Introduction

*Mycobacterium bovis*, the causal organism of bovine tuberculosis (bTB), was first isolated in European badger (*Meles meles*) in Britain in 1971^1^. Since then, it has been assumed that the disease in badgers has a causal association with the spread and maintenance of the disease in cattle. After over 25 years of uncertainty and expert recommendations, the Secretary of State for the Ministry of Agriculture Fisheries and Food commissioned a randomised field trial: the Randomised Badger Culling Trial (RBCT) 1998–2005, at a cost of circa £49 million^2^. This included 10 triplet areas of equal size (approx. 100 sq.km.) where adult and juvenile badgers were either (a) rapidly culled with a projected 70% population reduction ‘proactively’, throughout an entire cull area, (b) by culling reactively (all badgers culled locally around a confirmed incident) or (c) being left unculled as a control. Results of proactive culling were published in *Nature*^3^ [the 2006 paper]. This reported a significant reduction in bTB herd incidence ‘confirmed’ by visible lesions and / or bacterial culture *post mortem* following a positive comparative intradermal skin test ( SICCT ) in proactively culled areas, compared to areas where badgers were not culled. The reactive culling experiment was not fully completed.

The effect of proactively culling badgers was reported to decrease subsequent ‘confirmed’ herd incidents of bTB in cattle by approximately 19%. The 2006 paper remains the central evidence of a link between badger culling and bTB reduction, and upon which Government badger culling policy for England (with revisions from 2011 to 2020) has originated. The results were reiterated in correspondence to *Nature* in 2015 indicating that at least 70% of badgers should be culled to obtain the theoretical benefits^4^. This policy has cost many £10s of millions in implementation and in related commissioned research derived from the RBCT data and analysis in terms of modelling of likely outcomes of badger culls. Research into vaccinating badgers as a possible alternative to culling for bTB control in cattle also rests on the RBCT findings. These applications relate strongly to the veracity of the results of the 2006 paper, and many use the RBCT main conclusion to make practical risk pathway and disease control assumptions.

Badger culling, implemented by the UK Department of Environment, Food and Rural Affairs (DEFRA) since 2013, as a means of controlling bTB in cattle herds, has been rolled out across England. Around 230,000 badgers have been reported culled to-date, depleting badger populations locally by up to 95%, representing the largest ever destruction of a protected and recovering native wild species in the UK^5^. The policy is controversial, expensive and many believe it to be ethically unacceptable on animal welfare grounds and due to uncertainty over the potential ecological impacts of removal upon other wild species and habitats. In 2022, an additional tranche of eleven 4-year proactive badger cull licences and authorisations were granted.

In 2022, analysis of published government data (2013–2019) failed to detect any evidence that seven years of proactive badger culling had reduced bTB herd incidence in cattle but strongly suggested that improved cattle testing and other cattle related measures had brought significant benefits^6^. A subsequent study of a similar period, but with additional areas, confirmed similar reductions to those attributed to cattle testing and movement controls yet was not designed to distinguish the effects of different interventions^7^. Central to exploring reasons for the reported lack of an effect of proactive badger culling in England between 2013 and 2019, was the re-analysis of data generated from the RBCT using a range of statistical models to try to replicate previous results and to search for inconsistencies that might explain the contrasting results. The RBCT also suggested evidence for the ‘bTB perturbation effect hypothesis’^8^ proposing that disturbance of badgers caused by culling can increase bTB transmission between badgers and cattle. Consequently this study examines the strength of this hypothesis.

Further rationale and justification for the reanalysis of the data from which the results and conclusions of the 2006 paper are based is given in supplementary material 1.

## Materials and methods

Supplementary material of ‘the 2006 paper’^3^ was downloaded from the *Nature* website. It is also provided as supplementary materials (supplementary materials 2,3,4). All analyses were undertaken in R^9^ and the code and output is provided as supplementary material 5. A generalised linear modelling approach was used, similar to that of the 2006 paper. Thus, the RBCT data was initially analysed using a Poisson regression model to explore the association of badger culling with the number of ‘confirmed’ bTB herd incidents. Poisson regression has a count variable, in this case numbers of herd incidents, as the dependent variable. A Poisson model also assumes that the mean number of counts is equal to the variance of the mean number of counts. Here, a confirmed herd incident is one where at least one animal in the herd is confirmed to be infected by bTB by the discovery of a pathognomonic lesion and successful culture of *Mycobacterium bovis* from a sample following *post mortem* examination. This is often referred to in the veterinary literature as a “confirmed breakdown”. The data analysis was initially adjusted for over or under dispersion using a quasipoisson model. As an alternative, a generalized Poisson model was used, so models with over-dispersion could be analyzed, i.e. when there is significant departure from the assumption of the count data following a Poisson distribution. This has an additional parameter, in comparison to a Poisson, as the variance (ν) varies with the mean (µ) as ν = ɸµ. This becomes a Poisson when ɸ = 1, is over-dispersed if ɸ > 1, and under dispersed when ɸ < 1^10,11^. For this we used the glmmTMB package in R^12^. This allowed an information-theoretic approach for model comparison. This was based on the Akaike Information Criterion (AIC) and the small sample size correction of the AIC (AICc)^13^. We also applied model averaging to the competing models. Model averaging is a method of allowing model uncertainty in estimates which can provide better estimates and more reliable confidence intervals than model selection^14^. It involves calculating a weighted mean of the estimates obtained from each of the candidate models, with the weights reflecting a measure of the potential value of that model for estimation.

The data were also analysed using a linear (or Gaussian) model with the assumption that the dependent variable of ‘confirmed’ bTB herd incidence rate (i.e. number of herd incidences/exposure) was normally distributed. In addition, residuals were analysed to examine if models were correctly specified using the DHARMa package in R^15^. As an alternative, the data were also analysed using Bayesian regression models with minimally informative priors. Competing models were compared using marginal likelihood.

Additional analysis examined the effect of the latent (unobserved) incidence by estimating total herd incidence using the diagnostic parameters of the screening test. Thus, an ‘unconfirmed’ bTB herd incident is a herd in which there had been one or more individuals with a positive SICCT, but where no bTB lesions were found and there was a failure to culture bTB from samples following *post mortem* of these animals^16^^.^. In the veterinary literature this was often referred to as an “unconfirmed breakdown”. The SICCT is reported to have a specificity of 100% and a sensitivity (at the individual animal level) of approximately 50%^17^. Estimates of the sensitivity at the herd level, based on the number of herds diagnosed in the abattoir, that had passed the surveillance test has been suggested to be approximately 85%^18^. The incidence was adjusted accordingly in the analysis.

## Results and discussion

### Statistical models examining the effect of proactive badger culling

The most important result from the 2006 paper is presented in Table 1.

**Table 1:** Log-linear regression model fitted to ‘confirmed’ incidents (breakdowns) in whole trial areas (inner and outer regions combined), since the initial proactive cull, based on VETNET location data.

Parameter		Estimate	Overdispersion-adjusted SE	Overdispersion-adjusted p-value
Intercept		− 0.362	0.932	0.70
Treatment	Proactive	− 0.207	0.073	0.005
	Survey-only	–	–	–
Triplet	A	− 0.269	0.234	
	B	0.156	0.166	
	C	0.432	0.161	
	D	− 0.304	0.190	
	E	− 0.026	0.175	
	F	− 0.159	0.186	
	G	0.482	0.195	
	H	− 0.248	0.189	
	I	− 0.502	.0.197	
	J	–	–	–
Log of baseline herds		0.047	0.248	0.85
Log historic incidence*		1.241	0.213	< 0.001

* Historic incidence was calculated over a three-year period and was restricted to ‘confirmed’ incidents.

This is a reproduction from the supplementary information provided in the 2006 paper.

Here, the parameter treatment is highly significant and is interpreted as a reduction in the herd incidence of 18.7% in areas where badgers had been culled. What is a little unusual in this analysis is that the parameter Log of baseline herds was not fixed at 1. The rate is normally the count divided by some unit of exposure, in this case the exposure would be the number of baseline herds.

If exposure was the same across all triplets, then the log linear Poisson regression would be

$$log{\mu }_{x}={\beta }_{0}+{\beta }_{1}x+...$$

To control for baseline *B*_x_, as there is varying levels of exposure, this then becomes:

$$log\frac{{\mu }_{x}}{{B}_{x}}={\beta }_{0}+{\beta }_{1}x+...$$

 =  > 

$$log{\mu }_{x}={\beta }_{0}+{\beta }_{1}x+{logB}_{x}+...$$

The usual practice in Poisson regression with a variable exposure across groups would be to fix the parameters for *logB*_x_ at 1, and code it as an offset variable. This then would assume that the incidence in each triplet area is directly proportional to the number of baseline herds, that is if the number of baseline herds is doubled, there is a doubling of the incidence when other parameters are held constant. However, in the 2006 paper, the parameter for *logB*_x_ was not fixed at 1, but was analysed as an explanatory variable and hence estimated by the model. This could be a reasonable approach if there was some a priori reason why the number of herd breakdowns was not linearly associated with the number of herds in the baseline. All areas in each triplet had an area of approximately 100 km^2^. But with a relatively high variation in the number of herds in each area, there was considerable variation in herd density. Standard theory on infectious diseases does indicate that transmission is dependent on density (i.e. contact rate)^19^ so this might be a justification for not fixing the *logB*_x_ parameter at 1. Models suggest that disease transmission increases as the density or numbers of cattle and badgers increases^20^. Hence, with a fixed area for each triplet, the parameter of log(baseline) or log(herd years at risk) might be expected to be above 1. However, the parameter value that is estimated is close to, and not significantly different, from zero (Table 1). This is not consistent with a density dependent increase in transmission. Furthermore, the interpretation for this is that the number of bTB herd incidents is independent of the number of herds in a triplet area. That is, if the number of baseline herds is doubled, the incidence does not change. This does not seem to be credible.

Alternatively, the *logB*_x_ parameter could be fixed at 1 as an offset term. Doing this, and keeping the same variables in the regression and correcting for overdispersion (as reported in the 2006 paper), the result reported in Table 2 is obtained. This is model 2:

**Table 2:** Log-linear regression model fitted to ‘confirmed’ incidents (breakdowns) in whole trial areas (inner and outer regions combined), since the initial proactive cull, based on VETNET location data.

Parameter		Estimate	Overdispersion-adjusted SE	Overdispersion-adjusted p-value
Intercept		− 3.408	0.824	0.003
Treatment	Proactive	− 0.146	0.117	0.246
	Survey-only	–	–	–
Triplet	A	0.328	0.285	
	B	0.214	0.271	
	C	0.244	0.252	
	D	− 0.004	0.284	
	E	0.205	0.268	
	F	− 0.396	0.283	
	G	0.000	0.251	
	H	− 0.048	0.292	
	I	− 0.277	0.309	
	J	–	–	–
Log of baseline herds		1*		
Log historic incidence		0.741	0.263	0.023

*Fixed at 1. Codes as an offset variable.

Model 2 suggests that badger culling had no effect on confirmed incidents. However, the two models are not comparable by model selection techniques such as AIC or AICc as Model 1 is a Poisson model and model 2 is a quasipoisson model. Therefore, competing models were analysed using the generalized Poisson model which allowed direct comparison of over- or under-dispersed Poisson models. It is also possible to compare models with and without the explanatory variables presented in the 2006 paper. Since there are 20 data points, but up to 14 variables (9 for Triplet, baseline herds, historic incidence, treatment and over-dispersion or variance parameter), the small sample size AIC (AICc) was used for model selection and comparison. Model 3 is the full model with log(Baseline) as an explanatory variable. With model 3, the most parsimonious model was without triplet as an explanatory variable. Statistical parsimony is when a simpler model with fewer parameters is favored over more complex models with more parameters, provided the models fit the data similarly well. Models with the lowest AIC(AICc) are generally selected as the most parsimonious. With model 3, treatment (culling) had no effect on ‘confirmed’ incidents. Model averaging also indicated that there was no evidence that treatment had an effect. All models where triplet was included as an explanatory variable had a higher AICc than the null model with intercept only. The null model has no explanatory (independent) variables. This strongly indicates a degree of overfitting in models that included these parameters.

Supplementary information provided with the 2006 paper demonstrates that each triplet had a variable time at risk, ranging from 2.72 years (triplet D) to 6.73 years (triplet B) (Table 3). Thus, the effect of triplet in the 2006 paper is likely to be due to the variation in exposure time in addition to number of baseline herds. Time at risk was not explicitly included in the analysis of the data in the 2006 paper. There was an independent statistical auditor to oversee the analysis of the data generated by the RBCT. In the first report of the statistical auditor it was stated that, “to some extent, the number of triplets and the years of observation are interchangeable”^21^. This may have motivated the investigators in the 2006 paper to use the 9 categorical variables of triplet as an alternative to the single continuous variable of time at risk. Standard Poisson regression techniques would have time at risk and baseline herds included in the offset variables to fully control for exposure. Multiplying time at risk by the number of baseline herds achieves this, to give herd years at risk. This then can be included in the regression models as either an explanatory variable, or preferably, as an offset variable. It is not possible to use the categorical variables of triplet as offset variables. Model 4 repeats the analyses of models 2 to 3, but in this case using herd years at risk as a replacement for baseline.

**Table 3:** Incidence, baseline herds and incidence rates by triplet and treatment for trial areas and neighbouring areas. Figures are using the VETNET database and are for ‘confirmed’ incidents from initial cull.

Triplet	Treatment	Years since initial cull	Whole trial area			Neighbouring area		
			Incidence	Baseline herds	Incidence rate*	Incidence	Baseline herds	Incidence rate*
A	Proactive	5.60	37	71	9.3%	24	60	7.1%
A	Survey-only	5.60	52	86	10.8%	20	68	5.2%
B	Proactive	6.73	87	152	8.5%	65	154	6.3%
B	Survey-only	6.73	61	132	6.9%	44	68	9.6%
C	Proactive	5.85	29	105	4.7%	35	117	5.1%
C	Survey-only	5.85	84	174	8.2%	37	121	5.2%
D	Proactive	2.72	36	97	13.7%	17	47	13.3%
D	Survey-only	2.72	40	106	13.9%	15	57	9.7%
E	Proactive	5.28	36	116	5.9%	26	94	5.2%
E	Survey-only	5.28	56	97	10.9%	31	72	8.2%
F	Proactive	5.13	15	138	2.1%	13	61	4.2%
F	Survey-only	5.13	61	191	6.2%	32	127	4.9%
G	Proactive	4.82	72	245	6.1%	30	154	4.0%
G	Survey-only	4.82	40	131	6.3%	29	129	4.7%
H	Proactive	4.72	31	63	10.4%	46	73	13.3%
H	Survey-only	4.72	27	130	4.4%	27	94	6.1%
I	Proactive	2.91	27	100	9.3%	19	70	9.3%
I	Survey-only	2.91	21	98	7.4%	9	60	5.2%
J	Proactive	2.88	34	114	10.3%	32	123	9.0%
J	Survey-only	2.88	36	123	10.2%	19	102	6.5%

*Calculated as number of incidents (breakdowns) per baseline herd-year at risk.

This table is a reproduction from the supplementary information of the 2006 paper.

As when using baseline herds in the analysis, the only models that suggest an effect of treatment are models which included all the 9 explanatory variables of triplet. However, these models are not supported by AICc, with the most parsimonious model only including historical incidence and herd years at risk as explanatory variables. Likewise model averaging failed to support an effect of treatment on ‘confirmed’ bTB herd incidence rate.

### Linear model

The linear model. where the dependent variable was incidence divided by herd years at risk, is arguably more easily understood by the non-specialist. This was examined using model 5. Examination of the residuals indicated this was a suitable model for examination of the data. As with the Poisson models, there was insufficient statistical evidence to conclude treatment influenced ‘confirmed’ bTB herd incidents. The most parsimonious model did not include treatment as an explanatory variable.

### bTB Perturbation effect hypothesis

The 2006 paper reported an increase in herd incidence in the unculled areas neighbouring the areas where badgers had been culled, leading to the proposal that badgers might transmit bTB to cattle at enhanced rates after culling has commenced. However, using the same modelling approach (model 6) as for the badger cull areas, there is insufficient evidence to support the perturbation hypothesis. The most parsimonious model (AICc) does not include an effect of treatment.

### Latent breakdowns

The analysis confirms the result of the RBCT. That is the total herd incidents (confirmed and unconfirmed) have a similar rate of occurrence in areas where badgers were culled compared to control areas. This was analysed using a (generalized) Poisson model as described above (model 7). Again, there was insufficient evidence to conclude an effect of badger culling on the incidence of bTB at the herd level.

### Bayesian analysis

The model in the 2006 paper was also compared to the most parsimonious Poisson model and linear model using an alternative Bayesian analysis. Minimally informative priors were used. The same priors for the parameters were used in the model comparisons. As expected, a Bayesian analysis of the model from the 2006 paper gave almost identical parameter estimates. However, in terms of Bayes factor, the most parsimonious Poisson model was favoured over the model in the 2006 paper by a factor of 7.2 × 10^10^. There was no evidence of an effect of proactive culling on herd incidence. Indeed, the marginal likelihood indicated that models where the treatment covariate was removed completely from the model indicated better support. The posterior probability distributions of the parameters in the (overdispersed) Poisson models are given in Fig. 4.

All frequentist models, are summarized in Table 4. The R code and the R output for all model runs are given in the supplementary file 5.

**Table 4:** Statistical models analysed.

	Model	AIC	AICc	Number of coefficients	Effect of culling reduction (CI)	P (for culling)	Post hoc analysis#
1	Nature, 2006	142.3	202.9	13	6.2% to 29.5%	0.005	Quantile deviation significant. Some evidence of dispersion
2	log(baseline) fixed at 1*	NA	NA	13	− 8.5% to 31.4%	0.246	NA
3	Generalized Poisson model	133.2	217.2	14	12.3% to 24.6%	< 0.001	Quantile deviation qqplot OK
3a	Most parsimonious model	157.7	160.4	4	NA	NA	No issues detected
3b	Model average	NA	NA	NA	− 8.9% to 28.4%	NA	NA
4	Treatment retained, log(herd years at risk) fixed at 1	156.7	217.4	13	0.3% to 25%	0.045	No issues detected
4a	Most parsimonious model	166.6	168.1	3	NA	NA	Quantile deviation. qqplot OK
4b	Model average	NA	NA	NA	− 13.3% to 35.3%	0.311	NA
4c	4,but log(herd years at risk) as explanatory variable, not offset	133.2	217.2	14	12.2% to 24.6%	< 0.001	Quantile deviation. qqplot OK
4d	Most parsimonious model	151.5	154.2	4	NA	NA	No issues detected
4e	Model average	NA	NA	NA	− 4.7% to 25.9%	0.185	NA
5	Linear model	− 91.8	− 31.2	13	− 16.9% to 53.2%	0.615	Quantile deviation significant, some evidence of dispersion
5a	Most parsimonious model	− 84.3	− 82.2	3	NA	NA	No issues detected
5b	Model average	NA	NA	NA	− 37% to 41%	0.886	NA
6	Nature, 2006, perturbation	135.4	196	13	− 5.5% to − 57.2%	0.012	Some evidence of dispersion
6a	Log(baseline) replaced by log(herd years at risk). Log(herd years at risk) fixed at 1. Generalised Poisson model	150	210.7	13	− 33.0% to 9.1%	0.329	No issues detected
6b	Most parsimonious model	149.2	149.9	2	NA	NA	No issues detected
6c	Model Average				− 20.2% to 13.6%	0.597	NA
7	Nature, 2006, all breakdowns*	NA	NA	13	− 9.7% to 22.7%	0.384	NA
7a	Log(baseline) replaced by log(herd years at risk)*. Triplet removed. Incidence adjusted for herd level sensitivity of test. Generalised Poisson model	169.3	253.3	14	− 2.3% to 16.9%	0.127	Quantile deviation. qqplot OK
7b	Most parsimonious model	165.2	167.87	4	NA	NA	No issues detected
7c	Model average	NA	NA	NA	− 7.4% to 19.7%	0.081	NA

*Over or under dispersion modelled as a quasi-Poisson model.

^#^ Analysed by simulating residuals with the R package DHARMa.

AIC and AICc are only comparable between models with the same dependent variable on the same scale. Thus linear models cannot be compared to Poisson models. The most parsimonious model is the one with the lowest AICc value in any one group of models (ie model grouped as 3,4, 5, 6 or 7). This was always the one in which the parameter “treatment” had been removed, thus indicating that there was no treatment effect of culling.

### Overdispersion

**Figure 1:**
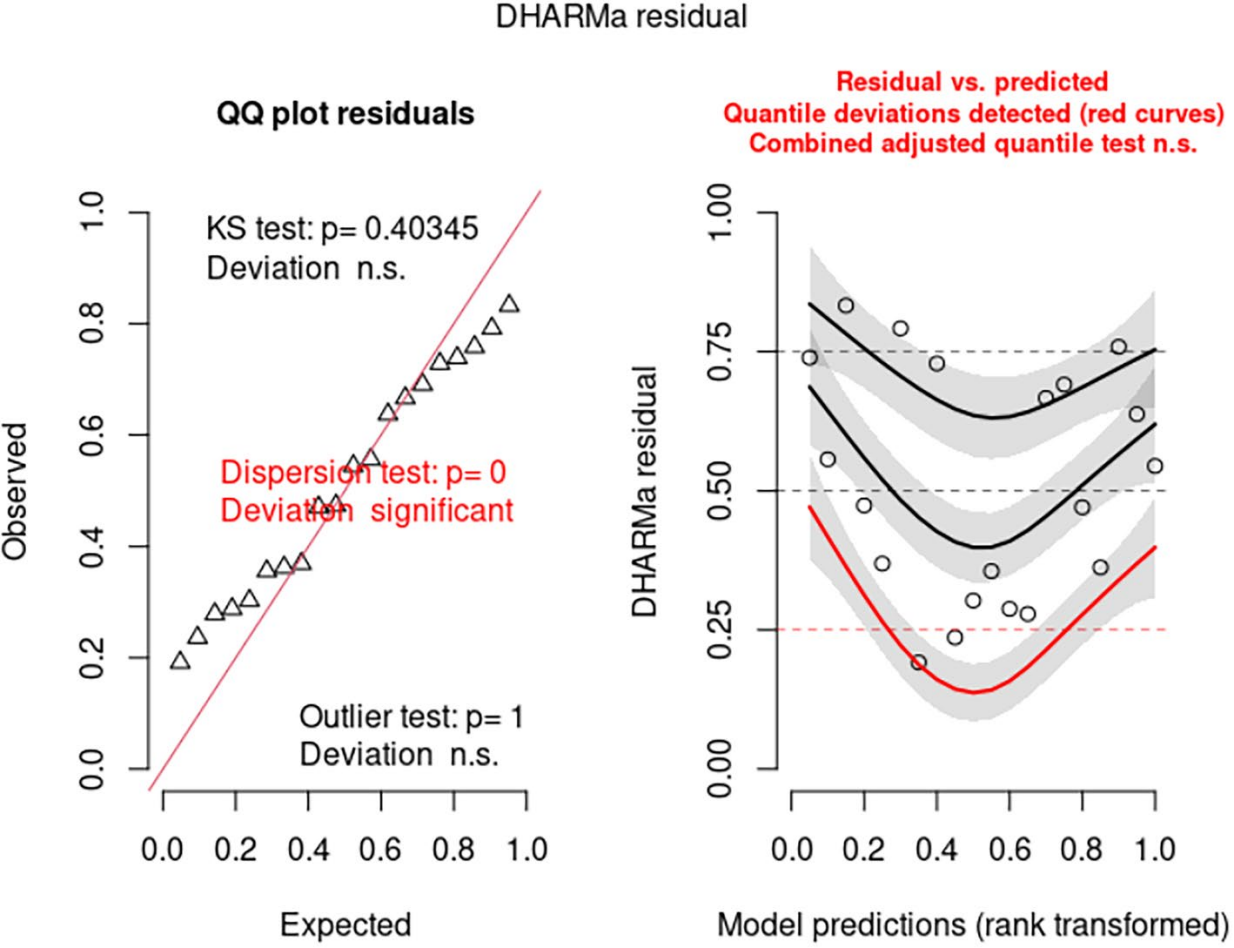
Analysis of residuals from the model reported in the 2006 paper.

The 2006 paper specifically indicated that RBCT results were corrected for overdispersion. However, the model indicated by the results in Table 1 (from the 2006 paper), had no evidence of overdispersion and was a Poisson regression model (model 1). Other statistical models that were explored usually had evidence of overdispersion. This was corrected by modelling as a generalized Poisson model. It was then possible to extract AIC and AICc for model comparison. Only the full model, which included triplet and treatment suggested a significant effect of treatment, and there was some evidence that there was underdispersion in this model. However, using AICc for model selection, it was clear that the most parsimonious model only had log(herd years at risk) and log (historical three year incidence) as significant explanatory variables. Model averaging confirmed the absence of an effect of treatment (i.e. badger culling). AICc is now recommended for model selection rather than AIC, although the two information criteria converge with large sample sizes. An important aspect is that competing models should have normally distributed residuals. Details of the theory and assumptions can be found in Burnham and Anderson^13^, which includes examples of selection of Poisson models using AICc. For the model from the 2006 paper, there is evidence from the qqplot that the residuals are not normally distributed (Fig. 1), and this may be because there was under-dispersion and hence model misspecification. Better specification can be achieved with the generalized Poisson model. This also confirmed that, using the generalized Poisson model, the residuals were normally distributed and hence, the use of AICc for model selection is valid, at least for this set of models.

### Posthoc analysis

**Figure 2:**
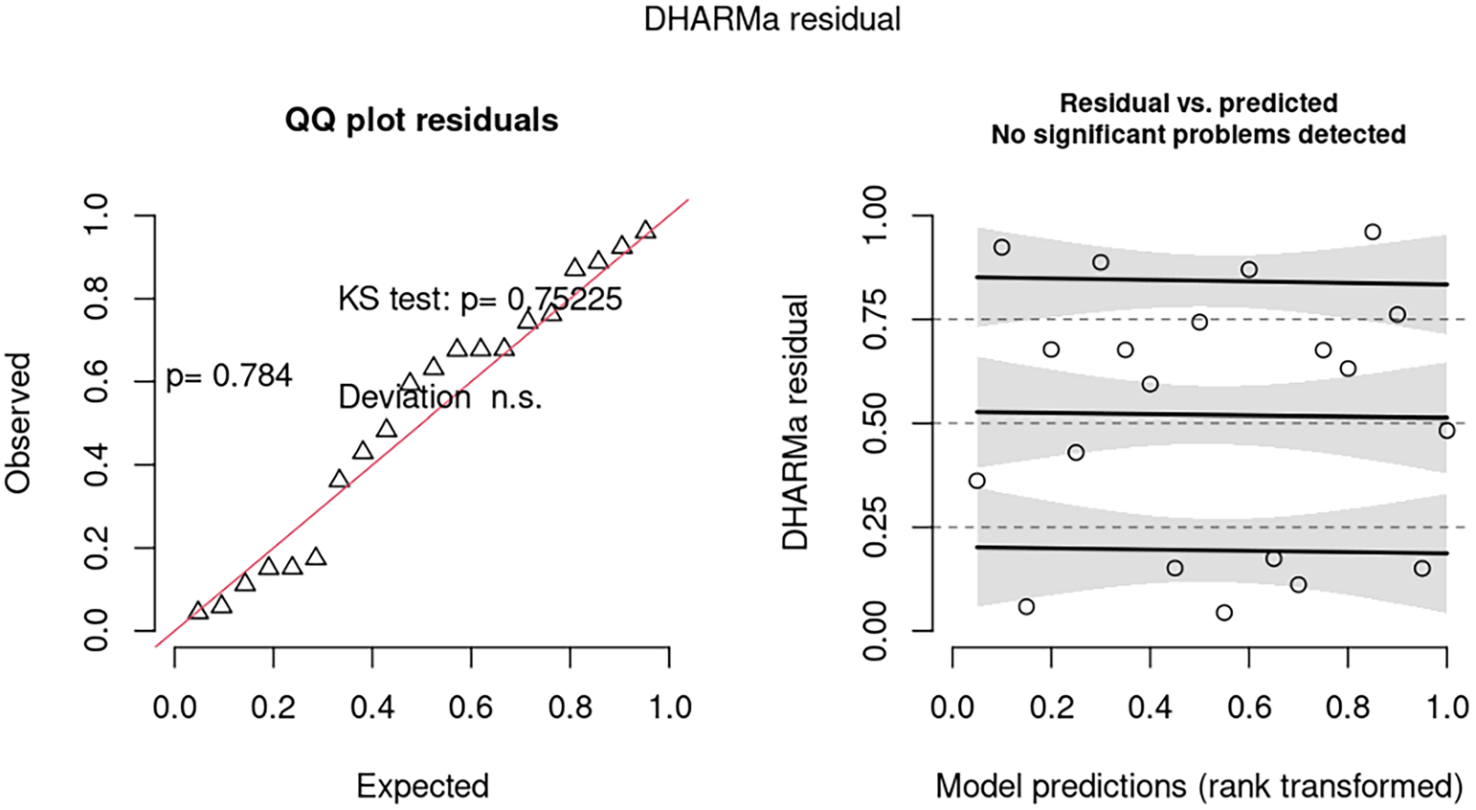
Analysis of residuals from the most parsimonious (over dispersed) (generalized) poisson model (model 4d).

**Figure 3:**
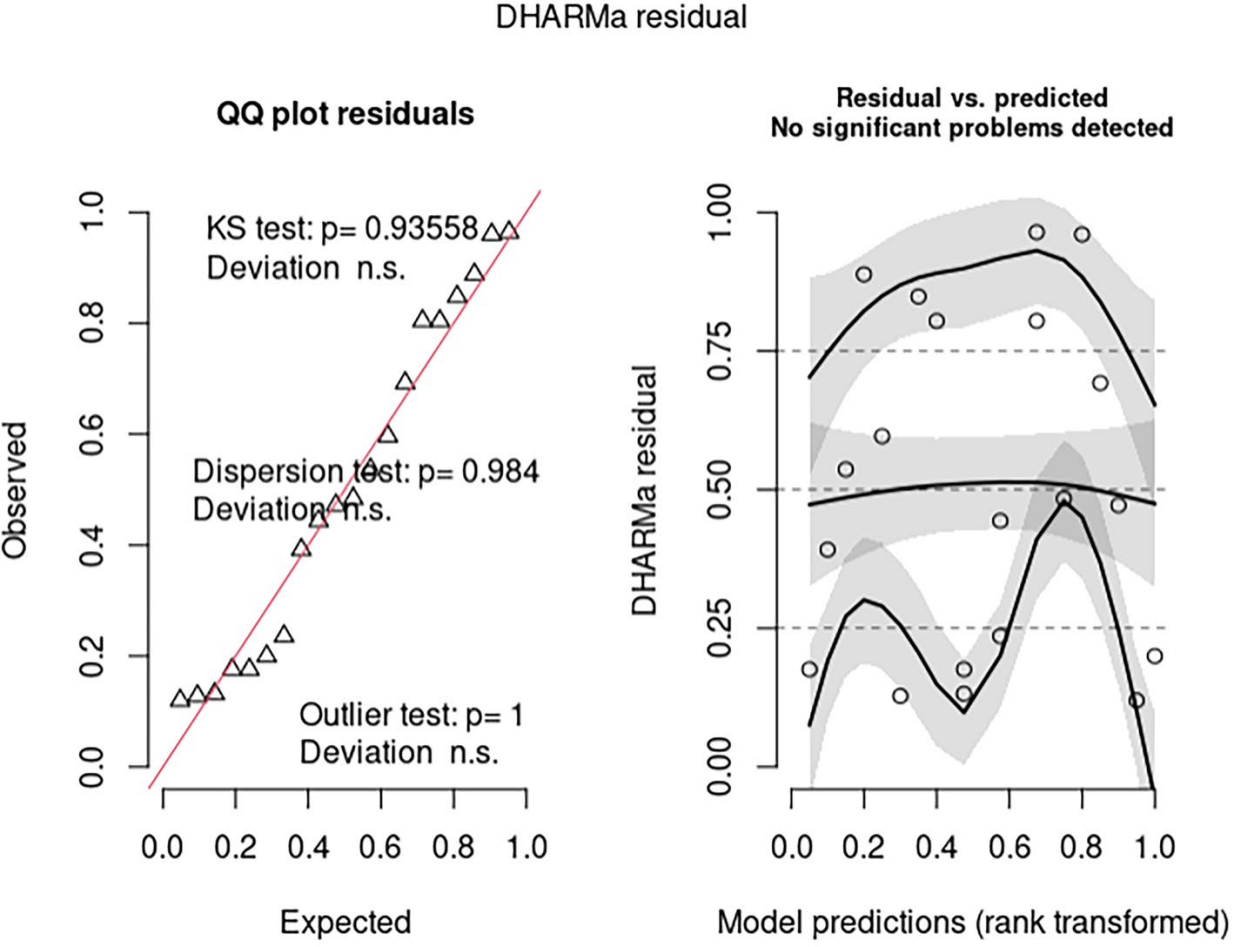
Analysis of residuals from the most parsimonious linear model without triplet as an explanatory variable. Treatment was removed as an explanatory variable as there was no significant effect.

Analysis of the residuals generated from the model in 2006 paper indicates there may be misspecification issues for model 1 (Fig. 1). The most parsimonious model, in terms of AICc, analyzed was model 4d. In this model, log(baseline) and Triplet were replaced by log(herd years at risk). Treatment was removed as an explanatory variable as there was no significant effect. This model had no misspecification issues (Fig. 2). The linear model 5a also had no misspecification issues (Fig. 3).

### Appropriate statistical modelling

**Figure 4:**
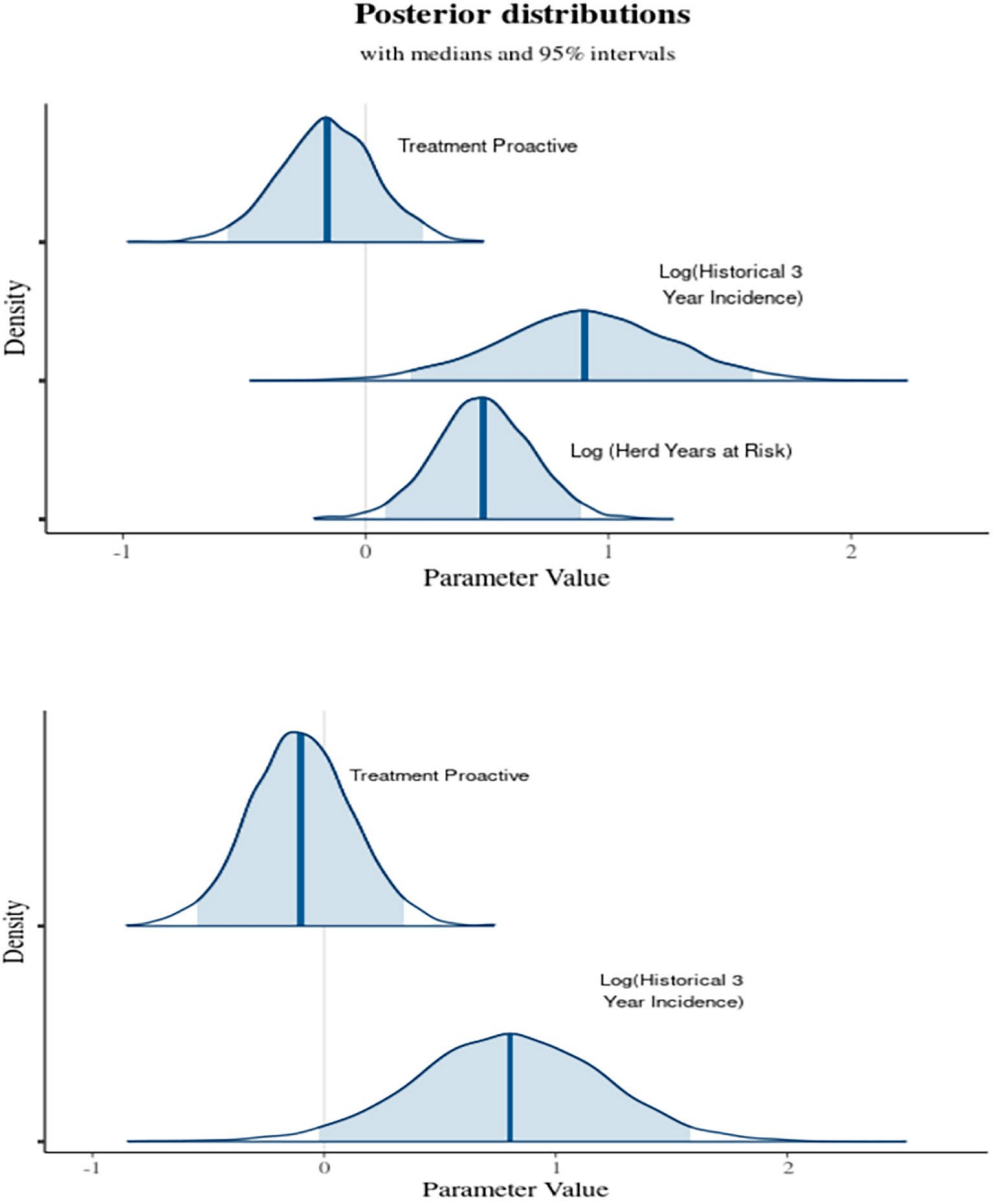
Density of the parameters following the Bayesian analysis of the most parsimonious frequentist Poisson model (top) with the log(herd years at risk) as an explanatory variable. The is analogous to the analysis in the 2006 paper but including time at risk. The 2006 paper had log(baseline herds) as an explanatory parameter. The parameter for proactive treatment has considerable parts of its density on either side of zero. Removing this parameter completely, improves the marginal likelihood and hence gives better support for the model. Below is the same model with log(herd years at risk) set as an offset.

Our analysis of the original RBCT data in the 2006 paper strongly suggests that there is insufficient evidence to conclude that badger culling reduced the rate of ‘confirmed’ herd incidents compared to control areas with no badger culling. The only statistical models that suggested an unequivocal effect was the model used in the 2006 paper and in the 2006 model where log(baseline) was replaced by log(herd years at risk) (model 4c). However, the interpretation of the results of these models, is that herd incidence of bTB was independent of number of herds studied (2006 model), or indeed the time at risk. Hence this reduces credibility from an epidemiology perspective. In addition, post hoc analysis of the statistical model used strongly suggests a degree of misspecification of the 2006 model. Although it could be argued, that in terms of AIC, this model produced the best fit of the count models, in terms of AICc it was statistically far more inferior to the better specified models (Fig. 4).

Examination of over-dispersed models, using the generalized Poisson model also suggested the best fitting model was one where treatment had no effect. Furthermore, the most parsimonious linear model, which analysed incidence rates, had no issues with misspecification and was also unable to detect an effect of culling badgers. Finally, a Bayesian analysis strongly suggested that there was little evidence for an effect of a proactive cull.

It seems likely that the statistical model reported in the 2006 paper was heavily overfitted. There were 13 free parameters, yet only 20 data points. As a result, the model is useful in reference only to its initial data set, which would include the specific idiosyncrasies of the data within each triplet, but it would have little predictive power.

Models with herd years at risk either as a covariate or, preferably, as an offset variable are more logical, they give more credible results and have the lowest AICc of competing models. A simple linear model of incidence rate as the dependent variable offered no evidence of misspecification and failed to show an effect of culling.

The aim of badger culling is to prevent bTB herd incidents. Concerns regarding undetected herd infections were raised in 2015, prior to the roll out of mass badger culling in England^22^. Since then, policy has altered to recognise ‘all SICCT reactors’ as potentially bTB infected for risk management purposes. Analysing the RBCT data to model total herd incidents, including undetected (i.e. latent) incidents, also indicated that culling had no effect on the herd incidence of bTB. When the statistical model included either baseline number of herds, or herd years at risk as a covariate rather than an offset, the parameter values were credible, indicating the number of herd bTB incidents increases both with years at risk and number of baseline herds, which is epidemiologically credible.

With regards to the veracity of the bTB perturbation effect hypothesis^23^, the present analysis demonstrates that any such effect is most likely to be an artefact of unsuitable analysis. Taken together, this considerably weakens any view that badgers are responsible for a substantial proportion of the transmission of bTB between cattle herds claimed by subsequent studies based upon the RBCT results. It also suggests that the data, rather than the statistical model, from the RBCT is consistent with the recently published paper by Langton et al.^6^, which failed to demonstrate a reduction in bTB incidence and prevalence associated with the culling of badgers.

It is also worth noting the data in Table 3, in the present report is taken directly from the supplementary material of the 2006 paper. In 6 of the proactive comparison areas, after treatment the incidence rates were higher in the survey only areas, but in 4 of the triplets the incidence rates were higher in the proactive area. There was also considerable heterogeneity ranging from a reduction in the proactive areas of 66% (Triplet E) to an increase of 236% (Triplet H). This, together with very marginal differences in some triplets (e.g. Triplet J and Triplet D), illustrates why there was likely to be no significant effect of culling in the present analysis.

### Implications

The model in the 2006 paper had a credibility issue because of the parameter value for baseline herds and evidence of misspecification in its post hoc analysis. Furthermore, models where there was no effect of treatment also had a lower AICc. A linear model assuming normal approximations had no evidence of misspecification and failed to demonstrate an effect of culling.

The present analysis has important implications. Much subsequent investment in research has assumed that the coefficient value (i.e. -0.207) in the RBCT results for the proactive culling parameter in the log linear Poisson model is a true reflection of the expected effect size for reduction in incidence in response to culling. There are many studies and reports which rely on this^20,24–30^. The view that the RBCT analysis represented a *‘strong evidence base involving experimental studies or field data collection on bTB with appropriate detailed statistical or other quantitative analysis.’*^31^ must now be considered unsafe. Re-examination of data from the 2006 paper, suggests otherwise and hence undermines the badger culling policy in England and Ireland. Other recent papers have suggested that badger culling has an effect on bTB incidence in cattle^32,33^. Both reports suffer the same statistical issue as the 2006 paper, that is the misuse of the exposure variable. In both these papers the authors report their results as incidence rate ratios (IRR), when in fact they report incidence ratios (IR), that is they did not correctly control for exposure by using an offset. Furthermore, these reports have other limitations such as sample size and short duration^6^. The present study is also strongly supported by recent results from Ireland, which suggests that the main drivers of the bTB epidemic there is cattle to cattle transmission with a minor role (if any) played by badgers^34^.

Vaccinating badgers as a possible alternative to culling for bTB control in cattle is also being trialled as an alternative to culling badgers to reduce transmission (for example Gormley et al.^35^). However, if culling badgers has no effect on the incidence of bTB in cattle, then vaccination of badgers is unlikely to reduce transmission to cattle. This is supported by the results of a recent trial in Ireland, with test, vaccinate and removal of badgers (TVR). Whilst this reduced the prevalence of bTB in badgers there was no effect on the incidence of bTB in cattle^36^. Of note is that this TVR study used an offset variable for exposure in their analysis, which is consistent with our analysis and in contrast to the 2006 paper.

The study suggests that the relationship between badgers, badger culling and bTB herd incidence is likely to be lower than has been reported and the relationship may be minimal or extremely infrequent. Our findings suggest that any method of controlling the disease in badgers, such as culling or badger vaccination are most probably equally likely to have a negligible effect in controlling tuberculosis in cattle.

## Conclusions

Prior to the RBCT it was felt that the results from several badger removal operations in England and Ireland demonstrate the importance of establishing adequate experimental controls, as some other unidentified factor could have been responsible for changes in herd breakdown rates^37^. Equally, most key studies cited in government policy that are subsequent to the RBCT that commenced in 1998, are derivative of it and use the RBCT model from 2006 to achieve their results and interpretations (For example studies reported by Jenkins et al.^24^, Bielby et al.^25^, Vial & Donnelly^26^, Vial, et al.^27^, Fenwick^28^, McCulloch et al.^29^ and Donnelly and Nouvellet^30^).

From the analysis presented, there is evidence that a controversial, expensive and disruptive programme of badger culling in England since 2013 has an inadequate scientific basis. It provides a clear example of an issue of scientific reproducibility. The present proactive cull in the High Risk Area of England is not reproducing the reported results of the RBCT. This is likely due to over-fitting of data in the 2006 paper resulting in it having a poor predictive value. In the 2006 paper it is unclear if other models were analysed, other than including additional covariates. Our results suggest that if alternative models had been analysed at the time, then great caution would have been given to concluding that badger culling has an effect on the herd incidence of bTB, whether “confirmed” or otherwise. Indeed the 2006 paper did state that there was no evidence of an effect on total herd bTB incidence, which our analysis reiterates. This further indicates that the intimation from the RBCT data remains that badger culling is unlikely to make any meaningful contribution to bTB control in cattle herds, but for different reasons, to those suggested by the RBCT analysis and report. While transmission of bTB to cattle by badgers may be possible in some circumstances, after extensive effort with experimentation and large applied culls, there is no evidence of an effect of badger culling on the incidence of bovine tuberculosis in cattle herds.

The results of the present report are consistent with other analyses that were unable to detect any disease control benefits from badger culling in England (2013–2019).

This study demonstrates a potential driver of the reproducibility crisis, in this case related to disease control management in an increasingly intensified livestock industry. A planned analysis for an epidemiological trial should usefully include a detailed analytical protocol that can be widely appreciated before and after the experiment.

### Supplementary Information

Supplementary Information 1.

Supplementary Information 2.

Supplementary Information 3.

Supplementary Information 4.

Supplementary Information 5.

## Data Availability

All data is available as supplementary material. It is also available as supplementary material in the 2006 paper.
