# Supplementary material for: Absence of effects of widespread badger culling on tuberculosis in cattle
Source: Sci Rep. 2024 Jul 15;14:16326. doi: 10.1038/s41598-024-67160-0 (PMC11251155; doi:10.1038/s41598-024-67160-0)
Supplement: Supplementary file 1 — Supplementary Information 1. [file 41598_2024_67160_MOESM1_ESM.pdf]

Supplementary Information 1 : Justification for study.

Absence of effects of widespread badger culling on tuberculosis in cattle

Paul R. Torgerson, Sonja Hartnack, Philip Rasmussen, Fraser Lewis, Thomas E. S. Langton

Introduction

For planned analyses, reappraisal with different models may be considered as unwarranted. However, with experimentation around the epidemiology of bovine tuberculosis and badger culling in the 1990s, the subject matter was fraught with uncertainties. Whether a planned analysis or not, the Randomised Badger Culling Trial (RBCT) findings benefit from examination after real-time application (English2013 onwards badger culls) and especially if there is discovery of concerns with aspects of the published experiment, that do not appear without re-running the data. There is increasing interest and concern regarding statistical uncertainty and reliance on single models to claim an effect (Wagenmakers, Sarafoglou and Aczel 2022).

Of central importance, the analysis undertaken in Donnelly et al. (2006) ‘the 2006 paper’, is not what is implied by the published methodology. When trying to repeat the analysis, results are different to those published. It was only by trying different approaches to those of the text of the 2006 paper, that the published result were matched. Anyone reading the 2006 paper but not re-running the data will have been given the wrong impression. Only after a careful and detailed examination of the 2006 paper’s Supplementary Information, does the anomaly become clear.

The fact that the most parsimonious approach to the analysis produces a non-significant finding, is why this discovery becomes as important as the original paper. It is arguably more important, due to the large volume of work since 2006 that have assumed the 2006 findings are sufficiently safe to extrapolate from, and to build upon.

The 2006 paper was published within just a few weeks of submission. It is notable that the methodological anomaly escaped the attention of the peer-reviewers, and it is suspected that they may not have re-run the analysis. There is some explanation as to how the anomaly happened in the published literature in 2010, as follows.

Does the issue of independent replication of the analysis question arise?

The response of Cox and Donnelly (2010) to Keiding (2010), some four years after the 2006 paper, in the Journal *Biostatistics*, discussed approaches using a range of statistical methods on a data set to compare their performance, and the choosing of a planned statistical approach that best compliments the subject matter. They proposed that selection of a specific statistical approaches may involve ***'subtle considerations about the interplay between subject-matter and statistical aspects and the detailed nature of the data and its compilation.'*** And with respect to the peer-review of results, they contend quite boldly: ***'the suggestion of requiring independent replication of specific statistical analyses as a general check before publication seems not merely unnecessary but a misuse of relatively scarce expertise.'***

By example, Cox and Donnelly offer the RBCT proactive badger culling findings, citing Donnelly et al. 2006, Donnelly et al 2007 and Jenkins et al. 2008. These publications, along with the independent audit of data handling, were said by them to provide the appropriate transparency necessary for a publicly funded disease control trial.

Krebs et al. (1997) had previously stated the importance of other researchers being able to use RBCT data, and this was further reiterated by Godfray et al. (2004). The availability of the data sets for independent study was also said by Cox and Donnelly (2010), to be 'welcome'. Cox and Donnelly placed emphasis on the value of data and analysis being available for discussion with subject matter specialists (in this case presumably veterinary epidemiologists with statistical knowledge) for formulation and investigation of new questions. This implied that a planned analysis with audit should effectively be a closed matter in terms of reproach. If this is the approach taken by the peer-reviewers to that manuscript in 2006, it explains how the significant problem developed.

Generally, concerns that subsequent analysis can be used to 'model dredge' for an alternative answer, do not apply to this case. The present study came about while attempting to repeat the original analysis and the discovering of an unusual treatment that was not clear in the published methodology. In particular, the reanalysis of the RBCT data was undertaken to try to discover why the conclusions were so different from those reported in Langton et al., 2022. This is completely different to any claim that the 2006 analysis is simply being challenged to disprove a theory established with a planned analysis. It does however counter any argument that checking prior to publication is "misuse of relatively scarce expertise". Checking and considering alternatives is undeniably good practice, particularly in medical, veterinary and biological applications. This paper therefore becomes evidence that counters the position of Cox and Donnelly in 2010 and does so using their own example.

The present study proves this point. It also checked material from the commencement of the RBCT study: the RBCT reports on statistical analysis (Bourne, et al. 1998, 1999, 2000), and the recommendations of statistical audits (Mollison, 2000, 2004, 2005). By and large, despite some disruption and limitations, the recommended approaches to experimental field methodology were followed in terms of practicalities of the field experiment. However, the commencement of triplets was highly staggered and disrupted and extended over time, by the Foot and Mouth Disease outbreak in 2001.

A first part of the RBCT experiment published earlier (Donnelly et al. 2003) is also an example where experimental process can go wrong. Here the published findings were found by government audit and independent review (Mollison 2004, Godfray et al. 2004) to lack the required statistical strength for significance. Yet they passed rapidly into the published record where they have been cited as if they were significant. Mollison's second RBCT audit agreed with an Independent Review Group and the Independent Scientific Group itself, that the reactive trials were abandoned prematurely, and in terms of sufficient data for scientific rigor, despite 'abandonment planning' being a pre-planned component of the first audit.

In Donnelly et al. (2003) 'the 2003 paper', of the ten planned triplets (paired comparisons) only three; A, B, and C, ran for more than two years, and three; E, F and G ran for less than 16 months (Table 1.).

Triplet	1999	2000	2001 No culling	2002	2003 (Actual duration)	Total duration	Triplet months	Total badgers removed, to August 2003
**A**		1		2	3 (3 months)	2 yrs, 3 months	27	117
**B**	1	2		3	4 (3 months)	3 yrs, 4 months	40	301
**C**		1		2	3 (3 months)	2 yrs, 3 months	27	395
**D**						1 month	1	61 ? VOID AS NO RESPONSE MEASURABLE IN 1 MONTH
**E**				1	2 (2 months)	1 year, 2 months	14	>62
**F**				1	2 (3 months)	1 year, 3 months	15	>145
**G**				1	2 (1 month)	1 year, 1 month	13	>172
**H**				1		1 year	12	17 LOW CULL NUMBERS: VOID
**I**				1		1 year	12	0 VOID
**J**						0	0	0 VOID

Table 1. Number of RBCT reactive cull areas active each year from 1998-2003 included in the 2003 paper. Showing cull duration per triplet and overall and problems with triplet D, H, I and J being of short duration or not done, compared with the planned four-year duration. Sources: Donnelly et al 2003, Bourne et al. 2007.

Inclusion of those running for up to 12 months (see Figure 2. of the 2003 paper) are questionable for epidemiological reasons, relating to the time needed for; a cycle of new infection; transfer of new infection; testing, and reporting. No triplets ran for the full four years. Of the four audits referred to in the 2003 paper, only one, (Mollison 2000) relates to badger culling efficacy. There, the auditor states *"I see the most important part of my future role as monitoring the ISG's statistical work once such analysis starts."* However, there is no further audit report prior to the 2003 paper, that like the 2006 paper, passed swiftly through peer review in the journal *Nature* in November 2003. It is not clear if the peer reviewer/s were taking the view of Cox and Donnelly (2010) on manuscript checking.

It is also notable that Godfray et al. (2004) found that after all the problems with the prematurely terminated reactive culling experiment, that it was ***"important that there is a single Project Manager responsible for the whole programme from data collection to analysis, and who can ensure the quality and consistency of the data. He or she should have a background that includes experience in statistical epidemiology."*** It is not clear if anyone was appointed.

Timeline of consideration on statistical approach

The First Annual Report of the Independent Scientific Group on Cattle TB to MAFF, (Bourne et al. 1998), refers to a study where the primary incidence analysis is based on bTB herd breakdown incidence rates:

"The primary analysis will be the comparison of the incidence of TB in cattle in the three treatment groups, initially on an intent-to-treat basis (i.e. all farms regardless of their co-operation with the trial will be included in the analysis). Incidence rates will be computed and analysed both on a per head of cattle and per farm basis."

Baseline herds and/or time at risk is needed as an offset in Poisson regression to calculate rates, but in the 2006 paper, rates were not used. So effectively the 2006 paper analysed incidence (or counts) rather than incidence rates.

Further, while the text indicates that analysis will be conducted on both tuberculin test-positive results and additional herds detected at slaughter ('confirmed' breakdowns), there is no indication that confirmed breakdowns only will be used, as was the case in the 2003 and 2006 papers.

"It is anticipated that the incidence of new herd breakdowns arising from routine tuberculin testing and from slaughterhouse detection will be differentiated and separate analyses will be undertaken."

The third Annual Report of the Independent Scientific Group on Cattle TB, (Bourne et al. 2001) simply refers to details provided to an Agricultural select committee (Bourne et al. 2000), recommending that the original data and accompanying analysis be verified by an independent expert. Professor D. Mollinson of Heriot-Watt University had been appointed by MAFF as its statistical auditor.

The fourth report (Bourne et al 2004) after the 2003 paper, said in respect of any results on the effect of badger culling on cattle TB incidence, that ***"an answer will become available by early to mid-2006, and this view is shared by the independent statistical auditor to the trial"*.** Table 3.6 reproduced below from the 4th report, indicates examination was to be made of triplet duration (Years At Risk), but this is absent from the final analysis and is not included as an offset.

Table 3.6 Variables tested to examine stability of treatment effects. Counts were log transformed before analysis.

Variable	Description
**1. Historical variables**	
Number of historical breakdowns	Confirmed herd breakdowns in the three years before the initial proactive cull or before the 2001 UK FMD epidemic for triplets first culled in 2002
Number of badgers culled in badger removal operations	Number of badgers caught during the interim strategy (April 1 1986 to December 12 1998)
Number of badger removal operations	Number of culling operations conducted during the interim strategy (April 1 1986 to December 12 1998)
Badger *M. bovis* prevalence in badger removal operations	Percentage of *M. bovis* positive badgers caught during the interim strategy
**2. Time related variables**	
Triplet duration	Total number of years since the initial proactive cull in the triplet
Calendar year of breakdown	Analysis conducted on yearly incidence by calendar year and adjusting for the number of days in the year
Year since enrolment	Analysis conducted on yearly incidence by the year since the initial proactive cull
Culling year	Analysis conducted on yearly incidence by culling year May 1 to April 30
**3. Trial badger variables**	
Number of active main setts	Number of active main setts identified during initial surveys before randomisation
Number of active setts	Number of active setts identified during initial surveys before randomisation
Number of badgers caught in initial and first follow-up proactive culls	
Initial badger prevalence	Percentage of *M bovis* positive badgers caught at initial proactive culls
**4. Other trial variables**	
Number of baseline herds	Baseline herds must have had a whole herd test in the five years before the initial proactive cull or during the trial and also have been in existence on VETNET
Trap opportunities	Percentage of all cage traps set to catch, which were available to catch badgers, i.e. that were not damaged, and did not catch non-target species etc.
Occupier compliance	Percentage of occupiers agreeing to cull and survey at the time of the analysis
Wildlife unit	The two Defra Wildlife Unit bases, which undertook trial fieldwork including culling operations.

Figure 1. Table 3.6 from Bourne et al. (2004)

In the section *Effect of the reactive treatment (para 3.39)*, statistical analysis of the effect of culling treatments has been undertaken at intervals since 2000 and the findings reported to the independent statistical auditor. Analyses, based on data on herd breakdowns up to 31 August 2003, reported that ***"reactive badger culling was associated with an estimated increase of 27% in the incidence of confirmed cattle herd breakdowns (95% overdispersion-adjusted confidence interval (CI): -2.4% decrease to 65% increase)."***

Despite the small sample size and an Independent Scientific Group recommendation to the Minister to continue the experiment until 2004 to gain the required data, the ISG felt the 'position' in relation to findings was unlikely to change significantly before then and the experiment was ended early. The

ISG had referred to a ‘controversial‘ statistical literature on early stopping of trials, but did not think such approaches were helpful. The early termination was surprising and may have related to the assumption of herds being caused to break down by the experiment, with legal implication for farm stock potentially infected, as well as the assumption of moral duty towards not causing further experimentally induced infection in domestic and wild animals. In any case, from a scientific perspective the decision to stop was surprising and the process involved was soon recorded as undesirable (Godfray et al. 2004). The duration of culling became inadequate for confident analysis as the confidence intervals included 0, casting doubt as to whether reactive culling was actually having any effect at all (King 2007).

Auditor reports

With proactive culling, Mollison stated in his 2000 report that he purposefully asked the two ISG statisticians Cox and Donnelly to set out their approach to the trials and this was subsequently presented (Bourne et al. 2000). Mollison stated that he subsequently held a meeting with the statisticians and the ISG's Chairman, Prof. John Bourne, to discuss details.

Use of a generalized Poisson model automatically deals with all the overdispersion issues which are frequently stated everywhere in the 2006 and related papers. However, doing so shows that there is no effect of badger culling on bTB herd breakdowns when there is the correct use of the offset for exposure and/or the most parsimonious model is selected by small sample size AIC (AICc). The absence of such an approach may have been due to an oversight by the statisticians who analysed the data in the RBCT, which the auditor failed to see. Possibly the auditor suggested the approach used by the two ISG statisticians for some reason.

Conclusions

From the beginning, RBCT (ISG) reports and MAFF audits indicate that there would be proactive cull analysis of <u>incidence ***rates*** by Poisson regression</u>, and this is what was passed as approved on numerous occasions. What was done however, was to analyse <u>***counts*** by Poisson regression</u>, a subtle yet critical change. The difference is the employment of the offset, which is the correct way to control for number of baseline herds and time at risk (exposure to disease). The use of the offset makes it an analysis of rate, equivalent to that used by Langton et al. (2022).

Placing baseline as an explanatory variable with a view that it was being made a rate, is possibly the fundamental mistake made. In later papers (Brunton et al. 2017; Downs et al 2019) a rate analysis is claimed, but again there is no offset, when this is what is correctly required.

The unusual analysis used in the 2006 paper complimented the findings of the incomplete reactive cull experiment and the 2003 paper.

For these reasons there are compelling reasons justifying this study:

a) To point out the disparity in the 2006 paper in terms of published methodology and what was done.
b) To show how the correct and plausible analysis produces different (opposite) results.
c) Alert the scientific community to the lack of support for the bovine TB and badger *perturbation effect hypothesis* and the serious weaknesses of the 2003 and 2006 analyses.
d) Draw attention to additional RBCT derivative studies that use the same methodology, and in order to question their validity too.
e) Draw attention to active government policy originating from 2003 and 2006 study outcomes and later publications that rest heavily or completely on them.

That the two main statistical authors for the 2006 paper should publish a case that such work need not be checked and simply used to inform plans for future study is interesting. It juxtapositions with the high level of challenge to RBCT reactive cull findings by farm veterinarians in 2003, and with government advice (King 2007). The reactive cull experiment published in 2003, had no further published qualification following subsequent audit and review.

A re-examination of the 2006 paper is both legitimate and has discovered significant matters of direct interest to the scientific community, of high policy and legislative relevance and of substantial public interest.

References

Brunton, L.A., Donnelly, C.A., O'Connor, H., Prosser, A., Ashfield, S., Ashton, A., Upton, P., Mitchell, A., Goodchild, A.V., Parry, J.E. and Downs, S.H. Assessing the effects of the first 2 years of industry ‐ led badger culling in England on the incidence of bovine tuberculosis in cattle in 2013–2015. *Ecology and evolution*, *7*(18), pp.7213-7230 (2017).

Bourne, J., Donnelly, C., Cox, D., Gettinby, G., McInerney, J., Morrison, I. and Woodroffe, R. Towards a sustainable policy to control TB in cattle (First Annual Report of the Independent Scientific Group on Cattle TB). MAFF Publications, London (1998).

Bourne, J., Donnelly, C., Cox, D., Gettinby, G., McInerney, J., Morrison, I. and Woodroffe, R. An epidemiological investigation into Bovine Tuberculosis (Second Annual Report of the Independent Scientific Group on Cattle TB). MAFF Publications, London (1999).

Bourne, J., Donnelly, C., Cox, D., Gettinby, G., McInerney, J., Morrison, I. and Woodroffe, R. Notes on statistical aspects of the badger culling trial (Memorandum to the Agriculture Committee – Annex A) (2000). http://www.defra.gov.uk/animalh/tb/publications/progress/isganna.htm

Bourne, J., Donnelly, C., Cox, D., Gettinby, G., McInerney, J., Morrison, I. and Woodroffe, R. An Epidemiological Investigation into Bovine Tuberculosis PB5801 Third Annual Report of the Independent Scientific Group on Cattle TB, Defra Publications, London (2001) .

Bourne, J., Donnelly, C., Cox, D., Gettinby, G., McInerney, J., Morrison, I. and Woodroffe, R. An Epidemiological Investigation into Bovine Tuberculosis Towards a science-based control strategy Fourth Annual Report of the Independent Scientific Group on Cattle TB, Defra Publications, London (2004).

Cox, D.R. and C. Donnelly, Commentary, *Biostatistics*, Volume 11, Issue 3, July 2010, Pages 381–382, (2010). https://doi.org/10.1093/biostatistics/kxq026

Downs, S.H., Prosser, A., Ashton, A., Ashfield, S., Brunton, L.A., Brouwer, A., Upton, P., Robertson, A., Donnelly, C.A. and Parry, J.E., Assessing effects from four years of industry-led badger culling in England on the incidence of bovine tuberculosis in cattle, 2013–2017. *Scientific Reports*, *9*(1), pp.1-14 (2019).

Donnelly, C.A., Woodroffe, R., Cox, D.R., Bourne, J., Gettinby, G., Le Fevre, A.M., McInerney, J.P., Morrison, W.I. Impact of localized badger culling on tuberculosis incidence in British cattle. Nature. Dec 18;426(6968):834-7 (2003). doi: 10.1038/nature02192. Epub 2003 Nov 23. PMID: 14634671.

Donnelly, C.A., Gao, W., Johnston, W.T., Cox, D.R., Woodroffe, R., Bourne, F.J., Cheeseman, C.L., Clifton-Hadley, R.S., Gettinby, G. and Gilks, P. Impacts of widespread badger culling on cattle tuberculosis: concluding analyses from a large-scale field trial, International Journal of Infectious Diseases, vol. 11, pg. 300-308 (2007)

Donnelly, C.A., Woodroffe, R., Cox, D.R., Bourne, F.J., Cheeseman, C.L., Clifton-Hadley, R.S., Wei, G., Gettinby, G., Gilks, P. and Jenkins, H. Positive and negative effects of widespread badger culling on tuberculosis in cattle, Nature, vol. 439, pg. 843-846(2006).

Godfray, H.C.J. et al. 2004. Independent Scientific Review of the Randomised Badger culling trials and associated epidemiological research. Report to Mr Ben Bradshaw MP 4th March 2000

Godfray, C., Curnow, R., Dye, C., Pfeiffer, D., Sutherland, W., & Woolhouse, M. Independent Scientific Review of the Randomised Badger Culling Trial and Associated Epidemiological Research (2004).

Jenkins, H.E., Woodroffe, R., Donnelly, C.A. The effects of annual widespread badger culls on cattle tuberculosis following the cessation of culling, International Journal of Infectious Diseases, 2008, vol. 12, pg. 457-465, (2008).

Keiding, N. Reproducible research and the substantive context *Biostatistics*, Volume 11, Issue 3, pp. 376–378 (2010). doi:10.1093/biostatistics/kxq033

King, D. Bovine tuberculosis in cattle and badgers. A report by the chief scientific advisor, Sir David King, Submitted to the Secretary of State, DEFRA, July 2007. (2007)

Krebs, J.R., Anderson, R.M., Clutton-Brock, T., Morrison, W.I., Young, D., & Donnelly, C.A. Bovine Tuberculosis in Cattle and Badgers. Ministry of Agriculture, Fisheries and Food (MAFF) Publications, London (1997).

Langton, T. E. S., Jones, M. W. & McGill, I. Analysis of the impact of badger culling on bovine tuberculosis in cattle in the high-risk area of England, 2009–2020. Veterinary Record 190, e1384 (2022).

Mollison, D. First report of the Statistical Auditor on the badger culling trial. Ministry of Agriculture, Fisheries and Food, London (2000).

Mollison, D. Statistical design of the badger culling trial (2nd audit) May 2004 (2004).

Mollison, D. Statistical design of the badger culling trial (3rd audit) December 2005 (2005).

Wagenmakers, E-J. & Sarafoglou, A. and B. Aczel (2022). One statistical analysis must not rule them all. Nature. 605. 423-425. 10.1038/d41586-022-01332-8.
