# Supplementary material for: Absence of effects of widespread badger culling on tuberculosis in cattle
Source: Sci Rep. 2024 Jul 15;14:16326. doi: 10.1038/s41598-024-67160-0 (PMC11251155; doi:10.1038/s41598-024-67160-0)
Supplement: Supplementary file 2 — Supplementary Information 2. [file 41598_2024_67160_MOESM2_ESM.pdf]

Supplementary Information 2 : Supplementary material from the 2006 study

Absence of effects of widespread badger culling on tuberculosis in cattle

Paul R. Torgerson, Sonja Hartnack, Philip Rasmussen, Fraser Lewis, Thomas E. S. Langton

Supplementary Information

Table of contents

1. Trial areas....2
   Land area....2
   Proactive culling operations....4
   Consent....7
   Measures of badger activity before the first proactive cull....8
2. Cattle TB incidence data....9
   Surveillance among herds in land neighbouring trial areas....11
3. Historical badger culling....15
4. Historic cattle TB incidence....15
5. Statistical analysis....16
   Overdispersion....16
   Paired t-tests....17
   Log-linear regression models, estimated treatment effects and predicted incidence 18
   P-values....19
6. Detailed results for confirmed breakdowns and all breakdowns....20
7. The search for systematic variation in the effect associated with proactive culling...31
8. Robustness of results to different measures of the size of the population at risk.......35
9. Interpretation of analyses from different time periods....38
10. Effects of widespread culling in previous studies....40
11. Literature cited....43

1. Trial areas

Land area

Analyses presented in the main paper, and in this Supplementary Information, concern twenty areas (ten proactive, ten survey-only, Figure SI1), each of which may be divided into three regions. The 'inner trial area' refers to the region falling ≥2km inside the trial area boundary, the 'outer trial area' lies <2km inside the trial area boundary, and the 'neighbouring area' describes land ≤2km outside the trial area boundary. (Figure SI2). Table 1 gives the area (in km^2) of each analysed region. Note that each trial area is the sum of the inner and outer regions, roughly 100 km^2 in size in total. The 'neighbouring area' was chosen to extend 2km outside trial area boundaries (unless otherwise noted) because ecological studies had shown effects on badger population density and ranging behaviour across this spatial scale[1].

Table 1 **Size of areas analysed by triplet, treatment and region. All figures are in km^2**

		Triplet										
Treatment	Region	A	B	C	D	E	F	G	H	I	J	Mean
Proactive	inner trial area	24.9	26.1	35.1	31.1	22.7	21.5	30.9	29.6	28.3	29.5	28.0
	outer trial area	70.8	73.8	70.0	67.8	82.5	74.1	71.0	65.7	71.5	71.3	71.8
	neighbouring area	103.5	104.0	101.8	98.7	106.1	53.4*	99.8	97.5	102.5	103.3	97.1
Survey-Only	inner trial area	27.6	15.9	30.2	32.0	18.4	32.3	28.1	25.8	28.5	33.0	27.2
	outer trial area	71.8	80.6	75.4	67.6	83.5	70.0	71.0	75.2	71.8	66.9	73.4
	neighbouring area	102.9	75.0	105.6	98.0	105.6	79.8	109.1	104.8	103.6	94.2	97.8

*The proactive trial area in triplet F borders on the sea and hence the neighbouring area is substantially smaller than those of other trial areas.

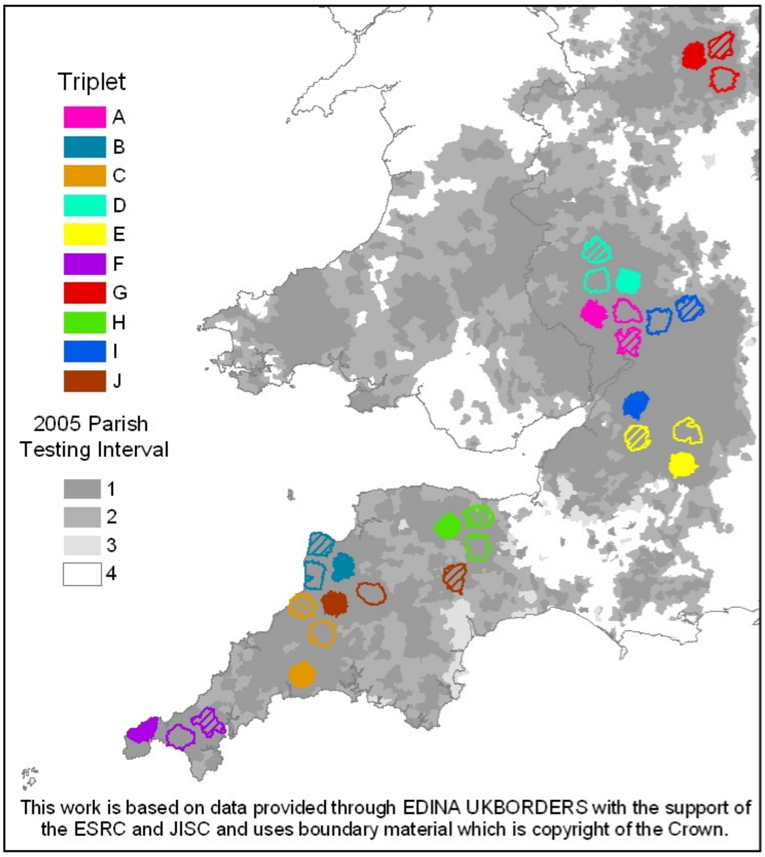

Figure SI1 Map of proactive (shaded), reactive (hatched) and survey-only (open) trial areas of the RBCT. Grey shading indicates parish testing intervals, which give an approximate index of local TB incidence; parishes with the lowest incidence are assigned four yearly testing (white) and parishes with the highest incidence are assigned annual testing (dark grey). Testing was conducted annually inside all trial areas. Data from reactive areas were not included in the analyses presented here; however the locations of these areas are shown because this influenced the inclusion of herds in 'neighbouring areas' (Figure SI2).

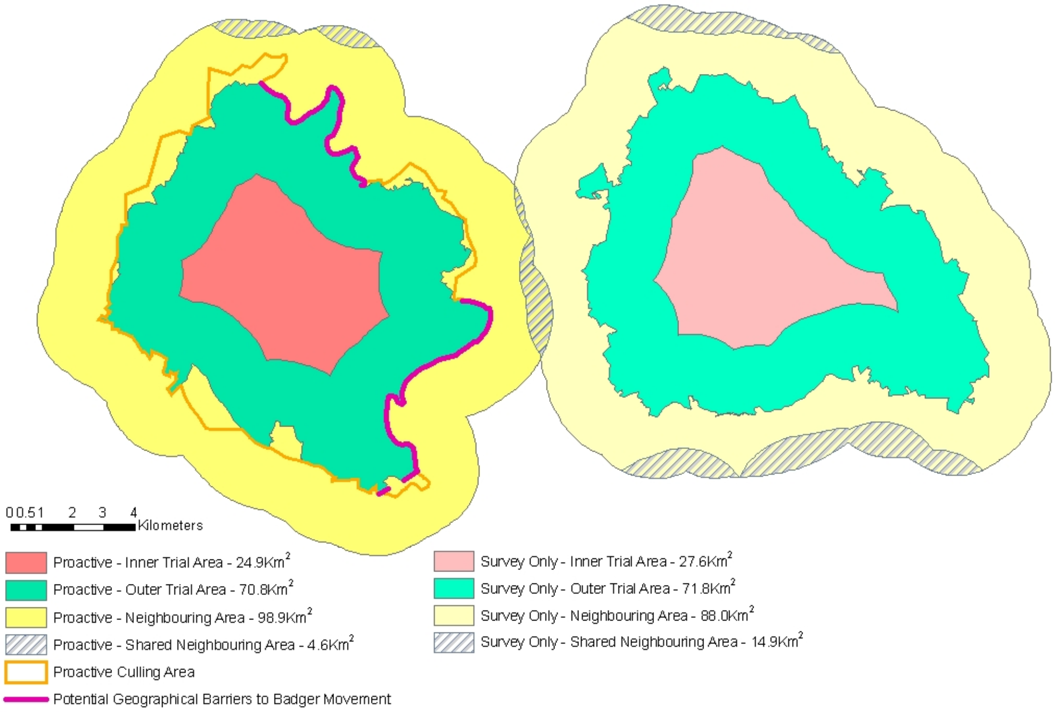

Figure SI2 Simplified map of proactive and survey-only areas of Triplet A, showing the size and disposition of the inner and outer trial areas, and neighbouring areas, relative to the area culled and possible geographical barriers to badger movement.

Proactive culling operations

Table 2 gives the date of each proactive cull to date. Table 3 gives the number of badgers proactively culled (to September 2005) by triplet and culling year. The numbers of badgers taken on particular culls will reflect both the numbers of badgers present in the culling area at the time, and the efficiency of badger capture. At any one time, the size of the badger population available for capture will be affected by prior culling, immigration

of badgers into the culled area, and survival and reproduction of badgers not captured on previous culls. Populations are particularly large at the start of the culling year (May-June) because cubs first emerge from their setts in spring. Capture success is known to vary seasonally[2], and was also influenced by weather conditions and disruption. In seven of the trial areas, the largest numbers of badgers captured was on the initial cull, probably reflecting the initially large numbers of badgers available for capture. The three trial areas for which this was not the case (A, D and H) all received their initial culls in mid-winter when capture success is known to be lowest[2]; for these areas the largest numbers of badgers were captured on the first follow-up culls (which took place in early summer when capture success is usually high[2]). We therefore expected that the proactive culling treatment might be more thoroughly applied following the first follow-up cull, rather than the initial cull. Partly for this reason, we conducted analyses of cattle TB incidence from the dates of completion of the first follow-up culls, as well as from completion of the initial culls (see Section 9 below).

Table 2 Dates of proactive culling by triplet and cull number. Shaded areas indicate culls that occurred before the 2001 epidemic of foot-and-mouth disease

Triplet	Cull number 1	2	3	4	5	6	7
A	Jan 2000	May 2002	Nov 2003	May 2004	Oct 2005		
B	Dec 1998	Nov-Dec 1999	Aug 2000 - Jan 2001*	Nov-Dec 2002*	Jun 2003	Jul-Aug 2004	Oct 2005
C	Oct 1999	Jan 2001	Aug-Nov 2002*	Oct 2003	Jun 2004	Sep 2005	
D	Dec 2002	May 2003	Sep 2004	May 2005			
E	May 2000	Jan 2001	Jun 2002 - Jan 2003*	Jun 2003	Jul 2004	Sep 2005	
F	Jul 2000	May 2002	Dec 2003	Sep 2004	Jun 2005		
G	Oct-Nov 2000	Jul 2002	Jul 2003	Jun 2004	Jun 2005		
H	Dec 2000	Jun-Jul 2002	Sep 2003	May 2004	Jul-Aug 2005		
I	Sept-Oct 2002	Sep-Oct 2003	Oct-Nov 2004	Jul 2005			
J	Oct 2002	Jul-Aug 2003	Oct-Nov 2004	May 2005			

*Culling was performed in sectors between these times; all other culls were carried out simultaneously across the entire trial area

Table 3 Summary of badgers culled within proactive culling areas to November 2005

Culling year*	1998-1999	1999-2000	2000-2001	2002-2003	2003-2004	2004-2005	2005-2006	Total
Triplet								
A		55		149	52	58	48	362
B	238	85	74	49	172	111	58	787
C		246	111	126	132	187	162	964
D				293	369	211	179	1052
E			744**	96	258	213	148	1459
F			451	248	103	220	155	1177
G			427	205	144	103	117	996
H			162	231	71	73	53	590
I				219	175	93	172	659
J				441	187	109	109	846
Total	238	386	1969	2057	1663	1378	1201	8892

*Culling years run from 1st May-31st January, with a closed season 1st February-30th April to avoid killing mothers with unweaned cubs. No badgers were culled during May 2001-January 2002 due to the suspension of fieldwork during the 2001 epidemic of foot-and-mouth disease.

**Combined total for initial and follow-up cull completed in the same year

Consent

Consent to survey and cull was sought from all identified land occupiers in all trial areas before random allocation to the treatments (proactive culling, reactive culling or no-cull survey-only). The consent status could change during the course of the trial due to a change in the occupier of a particular parcel of land or the occupier changing his/her consent. Thus, when describing consent levels within trial areas, a particular time point must be specified.

In March 2005, 72% of occupiers in proactive areas agreed to culling, 16% agreed to survey, but not culling, and 12% refused access. In terms of land area within proactive trial areas, consent to cull was then available for 68% of the land; an additional 9% of land was available for surveying but not culling and 9% of land belonged to occupiers that refused access for survey or cull. Finally, 13% of land within proactive areas was ‘unsigned’ in that the occupier was not identified or no response was received regarding consent to cull. These proportions varied considerably by triplet with 82% of land available for cull in triplet B but only 43% in triplet F (Table 4).

Table 4 Percentage of proactive trial area land by consent status

Triplet	Cull	Survey (but not cull)	Refusal	Unsigned
A	75%	5%	6%	14%
B	82%	5%	6%	8%
C	73%	6%	11%	9%
D	75%	4%	5%	16%
E	66%	10%	4%	19%
F	43%	19%	20%	18%
G	67%	9%	6%	19%
H	65%	9%	19%	6%
I	65%	12%	7%	16%
J	80%	9%	6%	5%

Measures of badger activity before the first proactive cull

Surveys of badger activity were conducted prior to treatment allocation, on all land for which consent was granted. To ensure consistency of data collection, surveys were carried out without reference to survey maps prepared in the course of badger removal operations that occurred prior to the RBCT. Surveys in Triplets A, B, C, D, E, and F were carried out by the Defra (Department of Environment, Food and Rural Affairs) Wildlife Unit; those in Triplets G, H, I and J were wholly or partly performed by outside contractors due to resource constraints with the Wildlife Units. To ensure comparability of data, trial areas within a triplet were surveyed simultaneously, by the same Wildlife Unit or contractor.

Survey teams worked on foot, in one or more pairs. In agricultural land, they walked all field boundaries, and in woodland they walked regularly spaced transects; in all cases, surveyors followed any badger paths that they located. The locations of all signs of badger activity (setts, latrines, and paths) were recorded in the field on 1:10,000 maps and later transferred to a geographical information system (GIS; Arcview, www.esri.com). Surveyors also assessed the activity of each sett, recording the number of active and inactive holes, and the presence of fresh digging, bedding, and tracks. Surveyors used these data to reach a subjective assessment of whether each sett was a 'main sett' (the centre of activity for a territorial social group). Field surveys were subjected to independent audit[3].

For each trial area, we calculated indices of badger activity prior to the random allocation of treatments, based on the numbers of (i) setts; (ii) active setts; (iii) main setts; (iv) active holes; and (v) latrines recorded. Each of these indices gives a measure of badger density, albeit a very approximate one[4-6]. Based on log-linear regressions of these outcome variables, adjusting for triplet and (log transformed) area available for

surveying, there were no significant pre-treatment allocation differences in recorded survey results between proactive and survey-only areas prior to treatment allocation (p-values: 0.23, 0.31, 0.10, 0.38 and 0.39, respectively).

2. Cattle TB incidence data

Cattle herds are subject to regulations made under the Animal Health Act[7] and EU legislation which require regular surveillance by TB testing. Cattle TB tests are arranged by the State Veterinary Service (SVS) and conducted by veterinarians. Great Britain is divided into counties and parishes. Each parish is assigned a parish testing interval of between four years and one year, where parishes with the lowest incidence are assigned four yearly testing and parishes with the highest incidence are assigned annual testing. Routine whole herd tests are carried out in accordance with the parish test interval and additional tests can be conducted at any time, for example, in response to slaughterhouse checks or breakdowns in neighbouring herds. All herds within the RBCT have been required to have annual whole herd tests for TB throughout the trial.

Cattle are given the single intradermal comparative tuberculin test, which involves injecting purified protein derivative (PPD) from *M. bovis* into the skin of the animal at one site on the neck, and injecting PPD from *M. avium* at another. Three days later the test is interpreted based on the size of reaction in the skin. If the reaction to *M. bovis* is more than 4mm (under so-called standard interpretation) or more than 2mm (under severe interpretation) larger than the reaction to *M. avium*, then the animal is categorised as a 'reactor'. The herd is put under movement restrictions, all reactors are compulsorily slaughtered and subject to post-mortem, and tissue samples cultured for *M. bovis*. If either lesions characteristic of TB are identified at post-mortem or the *M. bovis* organism is cultured, the breakdown is classified as 'confirmed' and the severe interpretation of the skin test applied to remaining members of the herd. Otherwise

breakdowns are classed as ‘unconfirmed’. In addition, the Meat Hygiene Service inspects all cattle sent for slaughter and any suspected cases of TB are reported to the SVS. These incidents contribute to the recorded incidence of TB breakdowns. During the 2001 foot and mouth disease (FMD) epidemic, few routine cattle TB tests were conducted, delaying the detection of infected herds.

The data from cattle TB tests are managed within the Defra animal health information system VETNET, operated and maintained by the SVS. The Veterinary Laboratories Agency receives these cattle data on a 6-weekly basis, which provide information on TB tests and breakdowns throughout Great Britain.

The observed numbers of breakdowns (confirmed and unconfirmed) are available in the associated Excel spreadsheet. These are broken down by triplet and allocated treatment. One set of analyses used point locations of herds, available from VETNET, to identify herds located inside trial areas and to allocate them to the ‘inner’ and ‘outer’ regions of trial areas (Figure SI2). For a second set of analyses, based on the RBCT database, the allocation of herds to either the inner or outer trial area was performed using GIS data which attributed land parcels within trial areas to herds. The location of each herd was calculated as the point location of the centre of the largest land parcel, and these point locations were used to allocate herds to regions within trial area boundaries. The GIS data files used to allocate land parcels to inner and outer regions were amended to reflect changes in occupancy noted during the course of field work. As such, if a new occupier took over a land parcel, the link between the land parcel and the previous occupier (and any associated herds) was removed. Hence, whilst it is known that the herd associated with the previous occupier was within a trial area, retrospective allocation of such herds to either inner or outer regions was not possible on the basis of current data. For this reason there are 43 additional baseline or new herds identified as being in the whole trial area which could not be allocated to the inner or

outer regions. These additional herds were associated with six confirmed breakdowns and nine unconfirmed breakdowns since the initial culls (six confirmed and seven unconfirmed since the first follow-up cull).

This paper presents analyses of the cattle TB incidence data up to 4 September 2005. Incidence data from reactive culling areas were excluded from these analyses because this treatment was discontinued in November 2003 and any effects on cattle TB incidence could be inconsistent over the period under study. Thus, data from 20 trial areas (10 proactively culled and 10 survey-only) were analysed in all cases.

Surveillance among herds in land neighbouring trial areas

Herds in the land neighbouring trial areas could only be systematically identified through VETNET location data, since the RBCT database did not aim to include herds as far as 2km from the boundaries of trial areas. Although, as previously mentioned, all herds within RBCT areas are required to have annual whole herd tests for TB throughout the trial, herds on neighbouring land will be tested according to their parish testing interval. Because trial areas were specifically chosen to be located in areas of high TB incidence in cattle, we expected that most herds on land neighbouring trial areas would also be in high risk areas and thus be on annual testing. However, we examined data on testing intervals to determine whether, within a triplet, the herds neighbouring the proactive trial area were on similar testing intervals to the herds neighbouring the survey-only trial areas.

The current test interval data revealed that the large majority (96%, 2075 of 2163) of herds living on land neighbouring trial areas are tested annually, 3% (65 of 2163) are tested every two years, 0.2% (4 of 2163) are tested every three years and 0.9% (19 of 2163) are tested every four years. All herds located on land up to 2km outside proactive

and survey-only trial areas were included in the analyses regardless of their recorded testing interval.

There was a significant tendency overall (exact p<0.001) and in two of the ten triplets (H and J with a similar non-significant trend in triplet C, see Table 5) to have more herds with longer testing intervals in the areas neighbouring survey-only trial areas, compared with herds neighbouring proactive areas. When triplets C, H and J were excluded, there was no evidence in the other seven triplets of any difference in the testing interval among such herds (exact p=0.17). Furthermore, when data from triplets C, H and J were excluded from analyses of the incidence of TB breakdowns among herds in land neighbouring proactive and survey-only trial areas, the estimated effects (a 24.7% increase with 95% CI: 19.7% reduction to 93.7% increase from the initial cull; and a 19.9% increase with 95% CI: 37.4% reduction to 129.6% increase from the first follow-up cull) were very similar to those obtained from the analysis of data from all 10 triplets (a 28.8% increase with 95% CI: 5.0-57.9% increase from the initial cull; and a 21.7% increase with 95% CI: 6.9% reduction to 59.0% increase from the first follow-up cull), except that as expected the confidence intervals were considerably wider due to the reduced dataset. Of course, since this analysis is based on the most recent data on test interval for these herds on land neighbouring trial areas, it is possible that the increased incidence observed on land neighbouring proactive trial areas, relative to the land neighbouring survey-only trial areas, led to the test intervals among herds in the former being shortened. In any case, we have demonstrated that the small differences observed in the test intervals were not responsible for the estimated increased incidence among herds on land neighbouring proactive trial areas.

Table 5 The numbers of cattle herds* in land neighbouring trial areas (≤ 2km outside trial area boundaries), sorted by their routine testing interval

Triple	Treatment	Test interval (in years)				Total	Exact p-value
		1	2	3	4		
A	Proactive	69			1	70	>0.99
	Survey-	85			1	86	
B	Proactive	194				194	1
	Survey-	91				91	
C	Proactive	126	6			132	0.073
	Survey-	129	16			145	
D	Proactive	54	3		1	58	0.67
	Survey-	61	1		1	63	
E	Proactive	86	7	3	4	100	0.13
	Survey-	76	5			81	
F	Proactive	71				71	>0.99
	Survey-	146	1			147	
G	Proactive	167	1	1	3	172	0.37
	Survey-	141	4		2	147	
H	Proactive	84	2		1	87	**0.026**
	Survey-	100	12		5	117	
I	Proactive	79				79	1
	Survey-	65				65	
J	Proactive	140				140	**0.004**
	Survey-	111	7			118	
Total		2075	65	4	19	2163	

*These herd numbers include all baseline herds and in addition all herds that came into existence since the initial proactive cull for their trial area

Table 6 Incidence, baseline herds and incidence rates by triplet and treatment for trial areas and neighbouring areas. Figures are using the VETNET database and are for confirmed breakdowns from initial cull

			Whole trial area			Neighbouring area		
Triplet	Treatment	Years since initial cull	Incidence	Baseline herds	Incidence rate*	Incidence	Baseline herds	Incidence rate*
A	Proactive	5.60	37	71	9.3%	24	60	7.1%
A	Survey-only	5.60	52	86	10.8%	20	68	5.2%
B	Proactive	6.73	87	152	8.5%	65	154	6.3%
B	Survey-only	6.73	61	132	6.9%	44	68	9.6%
C	Proactive	5.85	29	105	4.7%	35	117	5.1%
C	Survey-only	5.85	84	174	8.2%	37	121	5.2%
D	Proactive	2.72	36	97	13.7%	17	47	13.3%
D	Survey-only	2.72	40	106	13.9%	15	57	9.7%
E	Proactive	5.28	36	116	5.9%	26	94	5.2%
E	Survey-only	5.28	56	97	10.9%	31	72	8.2%
F	Proactive	5.13	15	138	2.1%	13	61	4.2%
F	Survey-only	5.13	61	191	6.2%	32	127	4.9%
G	Proactive	4.82	72	245	6.1%	30	154	4.0%
G	Survey-only	4.82	40	131	6.3%	29	129	4.7%
H	Proactive	4.72	31	63	10.4%	46	73	13.3%
H	Survey-only	4.72	27	130	4.4%	27	94	6.1%
I	Proactive	2.91	27	100	9.3%	19	70	9.3%
I	Survey-only	2.91	21	98	7.4%	9	60	5.2%
J	Proactive	2.88	34	114	10.3%	32	123	9.0%
J	Survey-only	2.88	36	123	10.2%	19	102	6.5%

*Calculated as number of breakdowns per baseline herd-year at risk

3. Historical badger culling

Badger culling has formed a component of British TB control policy since the 1970's[8-10]. Because the trial areas were placed in areas of high TB risk to cattle, most had been subject to some form of badger culling under previous policies. The 'gassing strategy' (1975-1981) killed badgers on land surrounding breakdown herds, primarily by pumping hydrogen cyanide into their setts. In the wake of concerns about welfare aspects of gassing, the 'clean ring strategy' was introduced in 1981. This involved cage trapping badgers on land occupied by affected cattle herds, then on adjoining land, expanding outwards until no further infected animals were captured. In 1986 this approach was replaced by the 'interim strategy' which involved culling badgers only on land occupied by affected cattle herds. The last 'interim' culls were performed in 1998, prior to the start of the RBCT. Capture methods used under the interim strategy were similar to those used in the RBCT, except that no closed season was in operation; instead, lactating females were immediately released[8] and in some cases the operations would be suspended until later in the year to avoid capture of further lactating females.

The numbers of badgers culled under the interim strategy in each trial area are shown in Table 7.

Table 7 The numbers of badger culled under the "interim strategy" (between 1986 and 1998) on land that subsequently fell inside RBCT areas

	Triplet										
Treatment	A	B	C	D	E	F	G	H	I	J	Total
Proactive	115	399	199	67	203	480	0	55	385	78	1,981
Survey-Only	186	342	319	14	239	240	0	31	38	0	1,409

4. Historic cattle TB incidence

Unless stated otherwise, the historic incidence of cattle TB was calculated for each trial area, for the three-year period before the initial proactive cull, except in triplets D, I and J where it was calculated for the three years prior to the start of the

2001 epidemic of foot and mouth disease which resulted in the suspension of all field activity and most routine cattle testing for TB. We also considered historic incidence over periods of one and ten years.

An analysis restricted to survey-only trial areas (excluding both proactive and reactive trial areas) comparing the incidence of confirmed breakdowns (since the completion of the initial proactive cull within that triplet) in inner trial areas, outer trial areas and neighbouring areas found no significant differences (p=0.18) between these three unculled regions when triplet, baseline herds and historic TB incidence (calculated over three years) were adjusted for. This result demonstrates the comparability of the survey-only control areas during the RBCT.

The numbers of historic breakdowns (over one- and three-year periods) are presented in the associated Excel spreadsheet. As described in section 2, there are discrepancies between the numbers of herds in the whole trial area and the total of the herds in the inner and outer trial areas which will have given rise to discrepancies in the historic incidence data from the RBCT database. The 43 additional herds were associated with three confirmed breakdowns and five unconfirmed breakdowns in the one-year period and seven confirmed and six unconfirmed in the three-year period.

5. Statistical analysis

Overdispersion

The differences between proactive and survey-only areas sometimes varied significantly more than would have been expected, had breakdowns occurred at random in each trial area ($p<0.05$ for a test based on the magnitude of the model deviance allowing for the residual degrees of freedom). Such unexplained variation is known as extra-Poisson overdispersion. Standard errors, and therefore confidence intervals and p-values from log-linear regression models, were adjusted for overdispersion by using an

overdispersion-adjustment factor (or inflation factor), which is the square root of the model deviance divided by the degrees of freedom. For most of the analyses the inflation factor was relatively small, less than 1.5, and frequently non-significant; however, in the interests of caution this factor has been used to inflate standard errors, widen confidence limits and hence inflate p-values in all cases where its value was greater than 1.

Paired t-tests

The simplest method of analysis for the incidence data is the paired t-test. Because the data are in the form of counts, the logarithm is taken of the number of breakdowns for each proactive and each survey-only area (denoted n_P and n_S, respectively). The within-triplet difference between these logarithms ($D=\ln(n_P)-\ln(n_S)$) has approximate variance $1/n_P + 1/n_S$. The t-test is performed on the ten within-triplet differences. For example, in the case of confirmed TB breakdowns from the initial cull (within whole trial areas using VETNET location data) the mean within-triplet difference was -0.208, which corresponds to a 18.8% reduction, 100%*(exp(-0.208)-1), in confirmed TB breakdowns associated with proactive culling within whole trial areas. The p-value from the t-test was 0.32. It is noteworthy that this estimated 18.8% reduction is almost identical in magnitude to the significant (p=0.005) estimate obtained from the log-linear regression model (explained below) after adjustment for the number of baseline herds and historic incidence (calculated over three years). Furthermore, the overdispersion factor obtained from the log-linear regression model with no adjustment for historic incidence, 2.32 as reported in Table 9, is similar to that obtained from the paired t-test (the square route of the observed variance, 0.394, divided by the mean over the ten triplets of the expected variance ($1/n_P + 1/n_S$), 0.055, was equal to 2.67). Thus, it was clear that there was substantial variation left unexplained by this simple analysis.

Similar results for other comparisons led to the conclusion that more detailed modelling, through the use of log-linear regression models, was required.

Log-linear regression models, estimated treatment effects and predicted incidence

Log-linear regression was used to analyse the incidence data and predictions, such as those plotted in Figure 1 of the main text. Table 8 presents the fitted log-linear model for confirmed breakdowns in whole trial areas (inner and outer trial areas combined) since the initial proactive cull, based on the VETNET location data.

Table 8 Log-linear regression model fitted to confirmed breakdowns in whole trial areas (inner and outer regions combined), since the initial proactive cull, based on VETNET location data.

Parameter		Estimate	Overdispersion-adjusted SE	Overdispersion-adjusted p-value
Intercept		-0.362	0.932	0.70
Treatment	Proactive	-0.207	0.073	0.005
	Survey-only	-	-	-
Triplet	A	-0.269	0.234	
	B	0.156	0.166	
	C	0.432	0.161	
	D	-0.304	0.190	
	E	-0.026	0.175	
	F	-0.159	0.186	
	G	0.482	0.195	
	H	-0.248	0.189	
	I	-0.502	0.197	
	J	-	-	-
Log of baseline herds		0.047	0.248	0.85
Log historic incidence*		1.241	0.213	<0.001

* Historic incidence was calculated over a three-year period, and was restricted to confirmed breakdowns because the model was analysing confirmed breakdowns.

We calculate the estimated impact of proactive culling as:

$$\exp(-0.207)-1 = -0.187 = -18.7\%$$

and the 95% confidence bounds are calculated as:

$$\exp(-0.207 - 1.96*0.073)-1 = -0.295 = -29.5\% \text{ and}$$

$$\exp(-0.207 + 1.96*0.073)-1 = -0.062 = -6.2\%.$$

Furthermore, we can calculate the predicted number of breakdowns in a proactive trial area, had that area received no culling. For example, the triplet B proactive trial area had 152 baseline herds and 42 historic breakdowns. Thus, the predicted incidence in this trial area, had that area received no culling, would be:

$$\exp(-0.362 + 0 + 0.156 + 0.047 \ln (152) + 1.241 \ln (42)) = \exp(4.670) = 106.7$$

confirmed breakdowns since the initial proactive cull in triplet B. This value was plotted against the observed value, 87, in Figure 1 of the main text.

For comparison, the predicted incidence in the triplet B proactive trial area, having received proactive culling would be

$$\exp(-0.362 - 0.207 + 0.156 + 0.047 \ln (152) + 1.241 \ln (42)) = \exp(4.463) = 86.8$$

(18.7% lower than the prediction had the area received no culling) which is very similar to the observed value, 87.

P-values

All p-values for tests of effects associated with proactive culling are two-sided. All p-values for tests of overdispersion are one-sided in that significant results will emerge only if significantly more variation (than expected) is observed.

6. Detailed results for confirmed breakdowns and all breakdowns

The estimated beneficial effect of culling was stronger inside ‘inner trial areas’ (≥2km inside trial area boundaries (Figure SI2)), which showed a 20% reduction following initial culls (p=0.25, 95% CI: a 46% reduction to an 17% increase (Figure SI3b)), than in ‘outer trial areas’ (<2km inside) which showed a 13% reduction (p=0.30, 95% CI: a 34% reduction to a 14% increase (Figure SI3c)). This gave a non-significant (p=0.72) suggestion that the effects of proactive culling might increase as one moves deeper inside trial area boundaries. However, if the adjustment for historical incidence is based on one year, rather than three years, of data, then the estimated beneficial effect was weaker inside ‘inner trial areas’ (≥2km inside trial area boundaries), which showed a 20% reduction (p=0.32, 95% CI: a 48% reduction to a 24% increase), than in ‘outer trial areas’ (<2km inside) which showed a 25% reduction (p=0.007, 95% CI: 7.5-38% reduction), giving a non-significant (p=0.80) suggestion that the effects of proactive culling decreased as one moves deeper inside trial area boundaries. This limited, and indeed contradictory, evidence of a within-trial-area trend will be the subject of further analysis.

We performed analyses only adjusting for the population at risk (in terms of the number of baseline herds) but found considerable overdispersion (Table 9 and Table 10). This unexplained variation was considerably reduced, to the extent that overdispersion was generally no longer significant, after adjusting for each trial area’s historic cattle TB incidence (calculated over three years). Adjustments based on historic cattle TB incidence calculated over 10 years were less successful, with overdispersion remaining significant in most cases. Adjustments based on historic cattle TB incidence calculated over one year performed similarly to the adjustment based on three years of historic data, though the primary comparison of incidence in whole trial areas from the initial proactive cull had the lowest overdispersion when the calculations were based on VETNET location data and adjustment was made for historic TB incidence calculated

over three years. Thus, we present detailed results (adjusting for historic cattle TB incidence calculated over three years and over one year) for confirmed breakdowns (Table 11and Table 13 respectively) and all (confirmed and unconfirmed) breakdowns (Table 12 and Table 14 respectively) for whole trial areas, inner trial areas, outer trial areas, and neighbouring areas. The key difference between the results obtained for the three- and one-year-based adjustments is that (as described above) the reduction in the incidence of confirmed breakdowns observed in outer trial areas is greater after adjustment for one-year historic TB incidence, making it similar to the reduction observed in the inner trial areas. The increased incidence observed in neighbouring areas is similar whether the adjustment for historic incidence is based on three or one year's data.

We also tested for a time-trend in the effect of proactive culling on TB incidence by stratifying the data by year (since the initial cull in each triplet, rather than calendar year) and fitting log-linear models allowing for an interaction between a linear time effect and the proactive treatment effect. These tests revealed no significant time trends. However, it was noteworthy that the estimates suggested a trend of increasing positive effects of proactive culling within trial areas (an estimated 5.2% greater reduction in TB incidence for each year since the initial cull, 95%CI: 16.1% greater to 7.0% less, p=0.39 for confirmed TB breakdowns in whole trial areas based on VETNET location data), whereas the estimates suggested a trend of decreasing negative effects of proactive culling within neighbouring areas (an estimated 3.7% smaller increase in TB incidence for each year since the initial cull, 95% CI: 17.3% smaller to 12.1% greater, p=0.63 for confirmed TB breakdowns in whole trial areas based on VETNET location data).

The 'neighbouring area' was chosen to extend 2km outside trial area boundaries because ecological studies had shown effects on badger population density and ranging behaviour across this spatial scale[1]. We also analysed incidence in a 3km-wide

‘neighbouring area’ (omitting any herds falling within 3km of more than one trial area). We found a non-significant trend of increased incidence on land neighbouring proactive areas (within 3km), relative to land neighbouring survey-only areas (within 3km), (11.4% increase, 95% CI: 15.7% reduction to 47.1% increase, p=0.45) based on confirmed TB breakdowns from the initial proactive cull. Furthermore, no evidence was found of an effect in this 3km-wide neighbouring area from the first follow-up cull (2.7% increase near proactive areas, 95% CI: 23.0% reduction to 36.9% increase, p=0.86). These reduced estimates (although in the same direction as the analysis of the 2km band) and correspondingly non-significant p-values lend support to our choice of 2km as the likely distance across which perturbation effects would be likely to operate.

Results for all breakdowns (both confirmed and unconfirmed) are qualitatively similar to those for confirmed breakdowns, but effects are weaker. Results using herd locations calculated from the RBCT database are likewise qualitatively similar although they tend to show greater overdispersion than analyses based on VETNET locations, thus we consider the VETNET-based results, after adjustment for historic incidence calculated over three years, as our key results.

Table 9 Overdispersion factors* and their significance levels for models estimating treatment effects based on confirmed TB breakdowns adjusted for triplet, baseline herds and varying durations of historic TB incidence. Higher overdispersion factors (and greater significance in the tests of overdispersion) indicate poorer model fit.

		Incidence adjusted for**							
		3 years		1 year		10 years		No historic	
		Factor	P-value	Factor	P-value	Factor	P-value	Factor	P-value
**Whole trial area[1]**	**Using VETNET location data**								
	From initial proactive cull	0.90	0.58	1.37	0.069	**1.87**	**0.001**	**2.32**	**<0.001**
	From first follow-up	1.15	0.24	1.24	0.15	**1.70**	**0.005**	**1.90**	**<0.001**
	**Using the RBCT location data**								
	From initial proactive cull	1.26	0.14	**1.52**	**0.023**	**2.01**	**<0.001**	**2.19**	**<0.001**
	From first follow-up	1.34	0.082	1.36	0.075	**1.84**	**0.001**	**1.83**	**0.001**
**Inner trial area[2]**	**Using VETNET location data**								
	From initial proactive cull	**1.44**	**0.044**	**1.64**	**0.009**	**1.59**	**0.014**	**1.70**	**0.003**
	From first follow-up	1.36	0.075	**1.46**	**0.037**	1.37	0.067	**1.50**	**0.022**
	**Using the RBCT location data**								
	From initial proactive cull	0.75	0.79	1.15	0.23	1.01	0.42	1.35	0.070
	From first follow-up	0.83	0.69	1.18	0.21	0.84	0.66	1.18	0.19
**Outer trial area[3]**	**Using VETNET location data**								
	From initial proactive cull	**1.59**	**0.013**	1.07	0.34	**2.09**	**<0.001**	**2.08**	**<0.001**
	From first follow-up	1.26	0.14	0.86	0.64	**1.65**	**0.008**	**1.63**	**0.007**
	**Using the RBCT location data**								
	From initial proactive cull	**1.66**	**0.007**	1.41	0.053	**1.93**	**<0.001**	**1.87**	**<0.001**
	From first follow-up	**1.43**	**0.044**	1.27	0.13	**1.64**	**0.009**	**1.55**	**0.014**
**Neighbouring area[4]**	**Using VETNET location data**								
	From initial proactive cull	1.03	0.38	0.94	0.52	1.24	0.15	**1.44**	**0.034**
	From first follow-up	1.15	0.24	1.20	0.19	1.21	0.17	1.29	0.10

1 Inner and outer regions combined; 2 More than or equal to 2km inside the boundary; 3 Up to 2km inside the trial area boundary; 4 Outside the trial area but within 2km of the boundary
*The overdispersion factor was estimated as the square-root of the deviance divided by the degrees of freedom.
**Significant effects are shown in bold type.

Table 10 Overdispersion factors* and their significance levels for models estimating treatment effects based on all (confirmed and unconfirmed) TB breakdowns adjusted for triplet, baseline herds and varying durations of historic TB incidence. Higher overdispersion factors (and greater significance in the tests of overdispersion) indicate poorer model fit.

		Incidence adjusted for**							
		3 years		1 year		10 years		No historic	
		Factor	P-value	Factor	P-value	Factor	P-value	Factor	P-value
**Whole trial area[1]**	**Using VETNET location data**								
	From initial proactive cull	**1.52**	**0.023**	**1.62**	**0.011**	**1.92**	**0.001**	**2.00**	**<0.001**
	From first follow-up	**1.50**	**0.028**	**1.52**	**0.024**	**1.64**	**0.008**	**1.66**	**0.005**
	**Using the RBCT location data**								
	From initial proactive cull	**1.74**	**0.003**	**1.72**	**0.004**	**2.06**	**<0.001**	**1.97**	**<0.001**
	From first follow-up	**1.67**	**0.007**	**1.67**	**0.007**	**1.82**	**0.002**	**1.71**	**0.003**
**Inner trial area[2]**	**Using VETNET location data**								
	From initial proactive cull	**1.52**	**0.023**	**2.01**	**<0.001**	**1.89**	**0.001**	**1.96**	**<0.001**
	From first follow-up	**1.70**	**0.005**	**1.91**	**0.001**	**1.74**	**0.003**	**1.79**	**0.001**
	**Using the RBCT location data**								
	From initial proactive cull	0.88	0.61	**1.50**	**0.027**	**1.47**	**0.034**	**1.41**	**0.043**
	From first follow-up	1.24	0.15	**1.42**	**0.050**	**1.46**	**0.037**	1.37	0.061
**Outer trial area[3]**	**Using VETNET location data**								
	From initial proactive cull	1.41	0.051	1.13	0.26	**1.66**	**0.007**	**1.67**	**0.004**
	From first follow-up	1.08	0.32	0.93	0.53	1.28	0.12	1.33	0.077
	**Using the RBCT location data**								
	From initial proactive cull	**1.49**	**0.029**	1.24	0.15	**1.61**	**0.011**	**1.51**	**0.019**
	From first follow-up	1.22	0.16	1.14	0.24	1.34	0.083	1.26	0.12
**Neighbouring area[4]**	**Using VETNET location data**								
	From initial proactive cull	1.21	0.17	0.86	0.64	1.37	0.069	**1.52**	**0.018**
	From first follow-up	1.08	0.32	0.94	0.52	1.14	0.25	1.27	0.11

1 Inner and outer regions combined; 2 More than or equal to 2km inside the boundary; 3 Up to 2km inside the trial area boundary; 4 Outside the trial area but within 2km of the boundary
*The overdispersion factor was estimated as the square-root of the deviance divided by the degrees of freedom.
**Significant effects are shown in bold type.

Table 11 Estimated treatment effects based on confirmed TB breakdowns adjusted for triplet, baseline herds and historic TB incidence (calculated over 3 years). Significant effects are shown in bold type.

		Proactive Effect	95% confidence bounds**		p-value for effect	Overdispersion* Factor	Overdispersion* p-value
**Whole trial area[1]**	**Using VETNET location data**						
	From initial proactive cull	**-18.7%**	**-29.5%**	**-6.2%**	**0.005**	0.90	0.58
	From first follow-up	**-22.7%**	**-36.1%**	**-6.5%**	**0.008**	1.15	0.24
	**Using the RBCT location data**						
	From initial proactive cull	-11.9%	-26.2%	5.3%	0.17	1.26	0.14
	From first follow-up	-17.3%	-33.6%	3.1%	0.091	1.34	0.082
**Inner trial area[2]**	**Using VETNET location data**						
	From initial proactive cull	-20.3%	-45.8%	17.2%	0.25	**1.44**	**0.044**
	From first follow-up	-33.7%	-57.9%	4.3%	0.076	1.36	0.075
	**Using the RBCT location data**						
	From initial proactive cull	-3.7%	-26.6%	26.4%	0.79	0.75	0.79
	From first follow-up	-15.0%	-38.4%	17.4%	0.32	0.83	0.69
**Outer trial area[3]**	**Using VETNET location data**						
	From initial proactive cull	-13.2%	-33.6%	13.5%	0.30	**1.59**	**0.013**
	From first follow-up	-16.5%	-34.6%	6.5%	0.15	1.26	0.14
	**Using the RBCT location data**						
	From initial proactive cull	-8.5%	-30.8%	21.1%	0.54	**1.66**	**0.007**
	From first follow-up	-13.3%	-34.5%	14.6%	0.32	**1.43**	**0.044**
**Neighbouring area[4]**	**Using VETNET location data**						
	From initial proactive cull	**28.8%**	**5.0%**	**57.9%**	**0.015**	1.03	0.38
	From first follow-up	21.7%	-6.9%	59.0%	0.15	1.15	0.24

1 Inner and outer regions combined; 2 More than or equal to 2km inside the boundary; 3 Up to 2km inside the trial area boundary; 4 Outside the trial area but within 2km of the boundary
* The overdispersion factor was estimated as the square-root of the deviance divided by the degrees of freedom.
**Reported confidence intervals are adjusted for the overdispersion factor (if it was >1).

Table 12 **Estimated treatment effects based on all (confirmed and unconfirmed) TB breakdowns adjusted for triplet, baseline herds and historic TB incidence (calculated over 3 years). Significant effects are shown in bold type.**

		Proactive Effect	95% confidence bounds**		p-value for effect	Overdispersion* Factor	Overdispersion* p-value
**Whole trial area**[1]	**Using VETNET location data**						
	From initial proactive cull	-7.9%	-22.6%	9.5%	0.35	**1.52**	**0.023**
	From first follow-up	-9.3%	-25.5%	10.5%	0.33	**1.50**	**0.028**
	**Using the RBCT location data**						
	From initial proactive cull	-4.1%	-21.6%	17.3%	0.68	**1.74**	**0.003**
	From first follow-up	-4.5%	-23.5%	19.2%	0.68	**1.67**	**0.007**
**Inner trial area**[2]	**Using VETNET location data**						
	From initial proactive cull	-18.1%	-41.9%	15.5%	0.26	**1.52**	**0.023**
	From first follow-up	-32.3%	-57.6%	7.9%	0.10	**1.70**	**0.005**
	**Using the RBCT location data**						
	From initial proactive cull	-9.0%	-28.3%	15.4%	0.44	0.88	0.61
	From first follow-up	-23.0%	-46.1%	10.0%	0.15	1.24	0.15
**Outer trial area**[3]	**Using VETNET location data**						
	From initial proactive cull	-2.1%	-19.3%	18.8%	0.83	1.41	0.051
	From first follow-up	-0.4%	-15.9%	18.1%	0.97	1.08	0.32
	**Using the RBCT location data**						
	From initial proactive cull	0.9%	-18.6%	25.1%	0.94	**1.49**	**0.029**
	From first follow-up	2.3%	-16.5%	25.3%	0.83	1.22	0.16
**Neighbouring area**[4]	**Using VETNET location data**						
	From initial proactive cull	14.1%	-6.2%	38.9%	0.19	1.21	0.17
	From first follow-up	10.3%	-9.9%	34.9%	0.34	1.08	0.32

1 Inner and outer regions combined; 2 More than or equal to 2km inside the boundary; 3 Up to 2km inside the trial area boundary; 4 Outside the trial area but within 2km of the boundary

* The overdispersion factor was estimated as the square-root of the deviance divided by the degrees of freedom.

**Reported confidence intervals are adjusted for the overdispersion factor (if it was >1).

Table 13 Estimated treatment effects based on confirmed TB breakdowns adjusted for triplet, baseline herds and historic TB incidence (calculated over 1 year). Significant effects are shown in bold type.

		Proactive	95% confidence bounds**		p-value for effect	Overdispersion*	
						Factor	p-value
**Whole trial area[1]**	**Using VETNET location data**						
	From initial proactive cull	-16.4%	-31.0%	1.3%	0.067	1.37	0.069
	From first follow-up	**-21.0%**	**-35.4%**	**-3.4%**	**0.021**	1.24	0.15
	**Using the RBCT location data**						
	From initial proactive cull	-13.1%	-30.2%	8.2%	0.21	**1.52**	**0.023**
	From first follow-up	-19.3%	-35.7%	1.4%	0.066	1.36	0.075
**Inner trial area[2]**	**Using VETNET location data**						
	From initial proactive cull	-19.8%	-48.2%	24.1%	0.32	**1.64**	**0.009**
	From first follow-up	-32.8%	-58.6%	9.1%	0.11	**1.46**	**0.037**
	**Using the RBCT location data**						
	From initial proactive cull	-11.6%	-34.8%	19.7%	0.42	1.15	0.23
	From first follow-up	-23.8%	-47.0%	9.7%	0.14	1.18	0.21
**Outer trial area[3]**	**Using VETNET location data**						
	From initial proactive cull	**-24.5%**	**-38.4%**	**-7.5%**	**0.007**	1.07	0.34
	From first follow-up	**-25.7%**	**-40.2%**	**-7.7%**	**0.007**	0.86	0.64
	**Using the RBCT location data**						
	From initial proactive cull	-21.4%	-40.8%	4.3%	0.095	1.41	0.053
	From first follow-up	-24.5%	-43.9%	1.6%	0.063	1.27	0.13
**Neighbouring area[4]**	**Using VETNET location data**						
	From initial proactive cull	**36.1%**	**10.0%**	**68.3%**	**0.005**	0.94	0.52
	From first follow-up	23.4%	-8.5%	66.5%	0.17	1.20	0.19

1 Inner and outer regions combined; 2 More than or equal to 2km inside the boundary; 3 Up to 2km inside the trial area boundary; 4 Outside the trial area but within 2km of the boundary
* The overdispersion factor was estimated as the square-root of the deviance divided by the degrees of freedom.
**Reported confidence intervals are adjusted for the overdispersion factor (if it was >1).

Table 14 Estimated treatment effects based on all (confirmed and unconfirmed) TB breakdowns adjusted for triplet, baseline herds and historic TB incidence (calculated over 1 year). Significant effects are shown in bold type.

		Proactive Effect	95% confidence bounds**		p-value for effect	Overdispersion* Factor	Overdispersion* p-value
**Whole trial area[1]**	**Using VETNET location data**						
	From initial proactive cull	-7.0%	-22.7%	12.0%	0.45	**1.62**	**0.011**
	From first follow-up	-8.7%	-25.2%	11.5%	0.37	**1.52**	**0.024**
	**Using the RBCT location data**						
	From initial proactive cull	-4.6%	-21.9%	16.5%	0.64	**1.72**	**0.004**
	From first follow-up	-5.3%	-24.2%	18.4%	0.63	**1.67**	**0.007**
**Inner trial area[2]**	**Using VETNET location data**						
	From initial proactive cull	-18.3%	-49.2%	31.4%	0.40	**2.01**	**<0.001**
	From first follow-up	-36.0%	-63.5%	12.3%	0.12	**1.91**	**0.001**
	**Using the RBCT location data**						
	From initial proactive cull	-20.1%	-42.9%	11.8%	0.19	**1.50**	**0.027**
	From first follow-up	**-32.0%**	**-53.3%**	**-0.9%**	**0.045**	**1.42**	**0.050**
**Outer trial area[3]**	**Using VETNET location data**						
	From initial proactive cull	-3.8%	-17.6%	12.3%	0.63	1.13	0.26
	From first follow-up	-2.1%	-16.5%	14.7%	0.79	0.93	0.53
	**Using the RBCT location data**						
	From initial proactive cull	-3.1%	-19.5%	16.5%	0.74	1.24	0.15
	From first follow-up	-0.6%	-18.5%	21.1%	0.95	1.14	0.24
**Neighbouring area[4]**	**Using VETNET location data**						
	From initial proactive cull	**24.1%**	**3.9%**	**48.3%**	**0.017**	0.86	0.64
	From first follow-up	17.6%	-4.4%	44.6%	0.12	0.94	0.52

1 Inner and outer regions combined; 2 More than or equal to 2km inside the boundary; 3 Up to 2km inside the trial area boundary; 4 Outside the trial area but within 2km of the boundary
* The overdispersion factor was estimated as the square-root of the deviance divided by the degrees of freedom.
**Reported confidence intervals are adjusted for the overdispersion factor (if it was >1).

Figure SI3 Observed and predicted (in the absence of culling) confirmed breakdowns in proactive areas, following the initial proactive culls, in (a) whole trial areas (total observed = 404, total predicted = 496.7); (b) inner trial areas (≥2km inside) (observed = 107, predicted = 134.3); (c) outer trial areas (<2km inside) (observed = 297, predicted = 342.1); and (d) neighbouring areas (≤2km outside) (observed = 307, predicted = 238.4). If the herd incidence rates in or near the proactive areas matched those in or near the survey-only areas (after adjustment for triplet, baseline herds and historic TB incidence), the points would fall on the solid lines. The dashed lines correspond to the reductions or increases in incidence recorded in or near the proactive areas. The prediction for each proactive area was obtained based on the specific features of that trial area, replacing the parameter associated with proactive culling for that associated with survey-only.

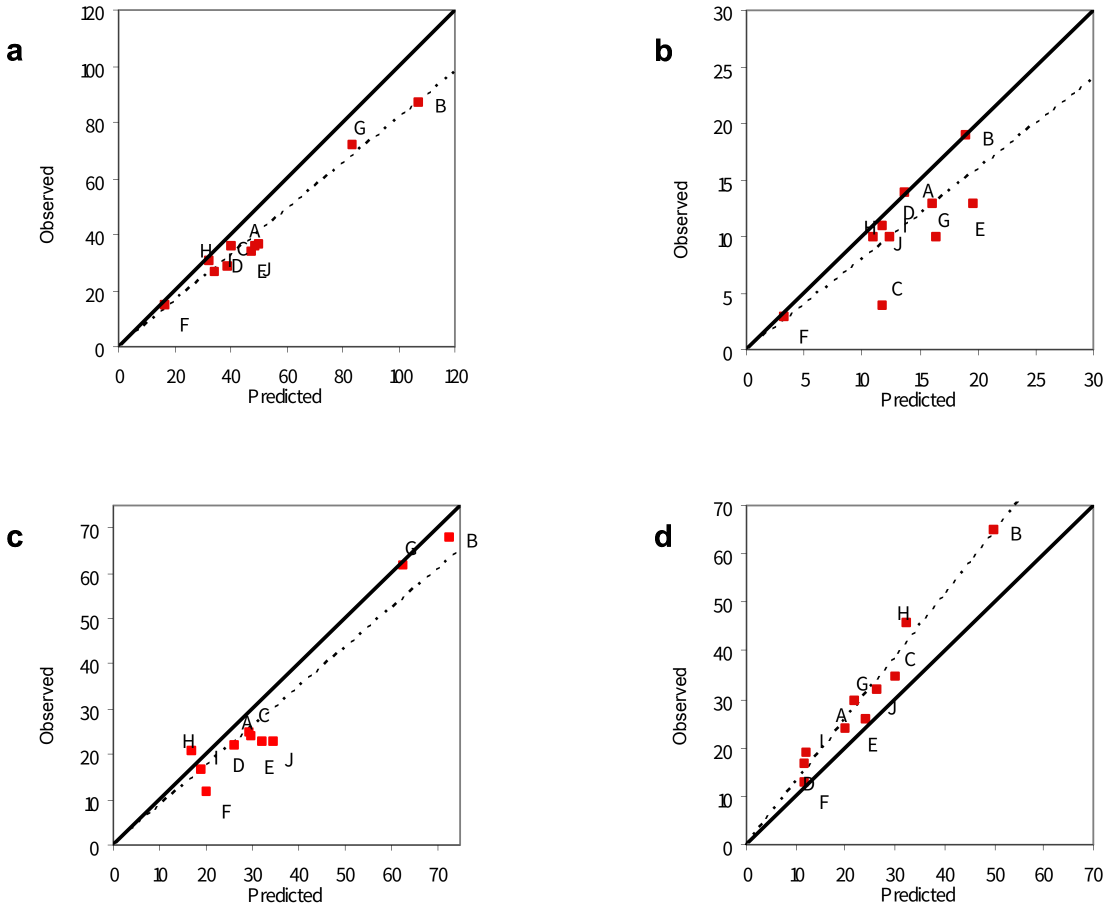

Figure SI4 Observed and predicted (as survey only) confirmed breakdowns in survey only areas, in (a) whole trial areas (total observed = total predicted = 478); (b) inner trial areas (≥2km inside) (observed = predicted = 132); (c) outer trial areas (<2km inside) (observed = predicted = 346); and (d) neighbouring areas (≤2km outside) (observed = predicted = 263). If the herd incidence rates in or near the survey-only areas matched the model predictions (which depend on the triplet, the number of baseline herds and historic TB incidence), the points would fall on the solid lines. The prediction for each survey-only area was obtained based on the specific features of that trial area.

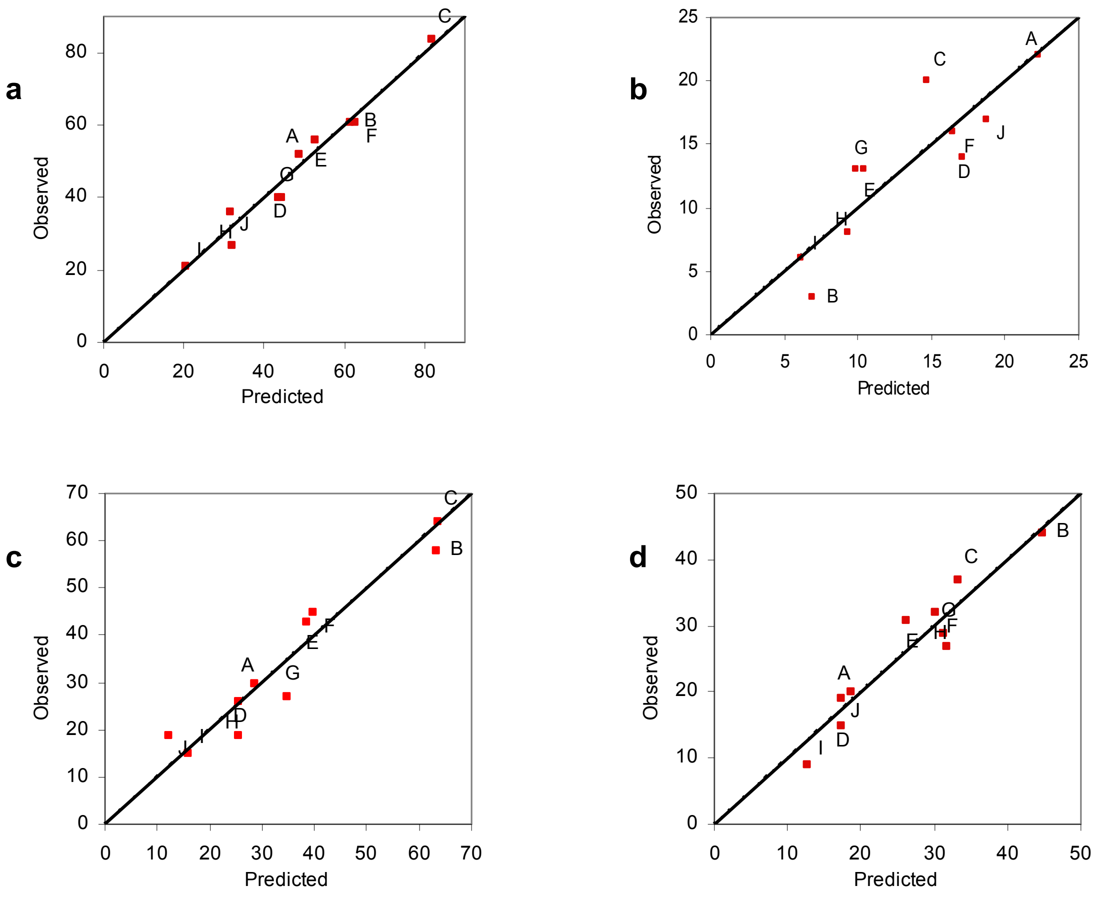

7. The search for systematic variation in the effect associated with proactive culling

We investigated whether the effect associated with proactive culling varied systematically between different triplets. By systematic, we mean that the variation can be explained in terms of a feature of the triplet or of the implementation of the strategy. In statistical terms, this would appear as an interaction between the so-called proactive treatment effect and the feature in question. An intensive search for such interactions has been made based on covariates including historic cattle TB incidence and other variables described in Table 15.

Table 15 Covariates tested for interaction with the treatment comparisons

Covariate	Description
**1. Historical variables**	
Number of badgers culled in interim badger removal operations*	Number of badgers caught during the interim strategy (April 1 1986 to December 12 1998)
Number of interim badger removal operations*	Number of culling operations conducted during the interim strategy (April 1 1986 to December 12 1998)
Badger *M. bovis* prevalence in badger removal operations	Percentage of *M. bovis* positive badgers caught during the interim strategy
**2. Time related variables**	
Triplet duration	Total number of years since the end of the initial proactive cull in the triplet
**3. Trial badger variables**	
Density of setts*	Number of setts identified during initial surveys before the random allocation of treatments divided by the estimated size of the surveyed trial area in km^2
Density of active setts*	Number of active setts identified during initial surveys before the random allocation of treatments divided by the estimated size of the surveyed trial area in km^2
Density of main setts*	Number of main setts identified during initial surveys before the random allocation of treatments divided by the estimated size of the surveyed trial area in km^2
Density of active holes*	Number of active holes identified during initial surveys before the random allocation of treatments divided by the estimated size of the surveyed trial area in km^2
Density of latrines*	Number of latrines identified during initial surveys before the random allocation of treatments divided by the estimated size of the surveyed trial area in km^2
Number of badgers caught*	Number of badgers caught in initial and first follow-up proactive culls
Proactive badger *M. bovis* prevalence	Percentage of *M. bovis* positive badgers caught at initial and first follow-up proactive culls
Permeability of trial area boundary for badgers	Percentage of the trial area boundary considered likely to substantially reduce badgers' ability to enter the area; barriers comprised coastline, major rivers, and motorways
**4. Other trial variables**	
Number of baseline herds*	Baseline herds must have had a whole herd test in the five years before the initial proactive cull or during the RBCT
Trap opportunities	Percentage of all cage traps set to catch, which were available to catch badgers, i.e. that were not damaged or removed, and did not catch non-target species etc.
Occupier compliance	Percentage of occupiers agreeing to cull and survey in August 2004
Wildlife Unit	The two Defra Wildlife Unit bases, which undertook trial fieldwork including culling operations.

*Values for these variables were log-transformed for the purposes of these analyses, with a value of 0.0001 when a value of 0 required log-transformation.

Table 16 presents results from tests for interactions between features of trial areas and the impact of the proactive treatment. The only significant interactions for the effect of the proactive treatment were those with the number of interim badger removal operations, and with the number of badgers culled in interim badger removal operations, for the analysis of all (confirmed and unconfirmed) breakdowns based on the VETNET location data. Proactive areas with higher numbers of interim badger removal operations were associated with a greater reduction in TB incidence, with the median number of operations (18) predicted to give rise to a 15% reduction in all TB breakdowns, from the initial proactive cull, whereas triplet B with the highest number of operations (54) was predicted to experience a 21% reduction in all TB breakdowns over this period and triplet G with no operations was predicted to experience a 54% increase, though this was the only triplet predicted to experience any increase. Similarly, proactive areas with higher numbers of badgers culled under the interim strategy were associated with a greater reduction in TB incidence, with the median number culled (157) predicted to give rise to a 14% reduction in all TB breakdowns, from the initial proactive cull, whereas triplet F with the highest number culled (480) was predicted to experience a 19% reduction in all TB breakdowns over this period and triplet G with no badgers culled was predicted to experience a 48% increase, though, once again, this was the only triplet predicted to experience any increase. The lack of any such effects among the confirmed breakdowns, which in most analyses were the most affected by proactive culling, suggests that these results should be regarded with great caution.

Table 16 P-values for the tests of interactions between treatments and the other covariates on the incidence of TB herd breakdowns in proactive versus survey-only whole trial areas. Significant effects are shown in bold type.

Covariate	Confirmed breakdowns		All (confirmed and unconfirmed) breakdowns	
	Using the RBCT location data	Using VETNET location data	Using the RBCT location data	Using VETNET location data
Number of historical breakdowns	0.74	0.71	0.43	0.93
Number of badgers culled in interim badger removal operations	0.095	0.25	0.34	**0.047**
Number of interim badger removal operations	0.062	0.21	0.23	**0.019**
Badger *M. bovis* prevalence in badger removal operations	0.80	0.16	0.98	0.36
Triplet duration	0.67	0.85	0.67	0.97
Density of setts	0.89	0.81	0.97	0.82
Density of active setts	0.96	0.61	0.80	0.71
Density of main setts	0.39	0.83	0.40	0.68
Density of active holes	0.70	0.81	0.88	0.94
Density of latrines	0.29	0.33	0.83	0.39
Number of badgers caught	0.94	0.58	0.79	0.71
Proactive badger *M. bovis*	0.90	0.97	0.80	0.98
Permeability of trial area boundary for badgers	0.94	0.75	0.83	0.97
Number of baseline herds	0.49	0.44	0.32	0.94
Occupier compliance	0.68	0.72	0.70	0.92
Trap opportunity	0.59	0.71	0.97	0.83
Wildlife Unit	0.56	0.98	0.47	0.84

8. Robustness of results to different measures of the size of the population at risk

Table 17 (for confirmed breakdowns) and Table 18 (for all – confirmed and unconfirmed – breakdowns) demonstrate the robustness of the estimated treatment effects to different measures of the size of the population at risk. (In all other analyses "baseline herds" is the measure of population size used.)

Table 17 Estimated treatment effects based on confirmed TB breakdowns adjusted for triplet and historic TB incidence and a measure of population size (a, b, c, or d as specified and defined in the table). Significant effects are shown in bold type.

		Proactive Effect	95% confidence bounds**		p-value for effect	Overdispersion*	
						Factor	p-value
**Using the VETNET location data**	**From initial proactive cull**						
	(a) baseline herds	**-18.7%**	**-29.5%**	**-6.2%**	**0.005**	0.90	0.58
	(b) baseline cattle	**-20.8%**	**-32.7%**	**-6.8%**	**0.005**	0.88	0.61
	(c) number of herd tests	**-18.8%**	**-29.5%**	**-6.4%**	**0.004**	0.90	0.58
	(d) number of cattle tested	**-20.2%**	**-31.2%**	**-7.4%**	**0.003**	0.87	0.62
	**From first follow-up**						
	(a) baseline herds	**-22.7%**	**-36.1%**	**-6.5%**	**0.008**	1.15	0.24
	(b) baseline cattle	**-24.5%**	**-39.5%**	**-5.6%**	**0.014**	1.17	0.21
	(c) number of herd tests	**-23.1%**	**-36.4%**	**-7.0%**	**0.007**	1.16	0.22
	(d) number of cattle tested	**-24.7%**	**-38.3%**	**-8.2%**	**0.005**	1.16	0.22
**Using RBCT location data**	**From initial proactive cull**						
	(a) baseline herds	-11.9%	-26.2%	5.3%	0.17	1.26	0.14
	(b) baseline cattle	-16.1%	-30.3%	1.0%	0.063	1.29	0.12
	(c) number of herd tests	-11.5%	-25.0%	4.4%	0.15	1.18	0.20
	(d) number of cattle tested	-14.7%	-29.6%	3.3%	0.10	1.33	0.089
	**From first follow-up**						
	(a) baseline herds	-17.3%	-33.6%	3.1%	0.091	1.34	0.082
	(b) baseline cattle	**-21.6%**	**-37.5%**	**-1.7%**	**0.035**	1.38	0.066
	(c) number of herd tests	-17.1%	-32.5%	1.9%	0.075	1.28	0.12
	(d) number of cattle tested	-20.4%	-36.9%	0.4%	0.055	1.41	0.052

* The overdispersion factor was estimated as the square-root of the deviance divided by the degrees of freedom

**Reported confidence intervals are adjusted for the overdispersion factor (if it was >1)

Table 18 Estimated treatment effects based on all (confirmed and unconfirmed) TB breakdowns adjusted for triplet and historic TB incidence and a measure of population size (a, b, c, or d as specified and defined in the table). Significant effects are shown in bold type

		Proactive Effect	95% confidence bounds**		p-value for effect	Overdispersion* Factor	Overdispersion* p-value
**Using the VETNET location data**	**From initial proactive cull**						
	(a) baseline herds	-7.9%	-22.6%	9.5%	0.35	**1.52**	**0.023**
	(b) baseline cattle	-6.0%	-25.7%	18.9%	0.61	**1.91**	**0.001**
	(c) number of herd tests	-8.3%	-23.0%	9.1%	0.33	**1.53**	**0.021**
	(d) number of cattle tested	-7.8%	-26.5%	15.8%	0.48	**1.94**	**<0.001**
	**From first follow-up**						
	(a) baseline herds	-9.3%	-25.5%	10.5%	0.33	**1.50**	**0.028**
	(b) baseline cattle	-5.7%	-27.0%	21.9%	0.65	**1.81**	**0.002**
	(c) number of herd tests	-10.3%	-26.7%	9.9%	0.30	**1.55**	**0.019**
	(d) number of cattle tested	-9.4%	-29.9%	17.0%	0.45	**1.91**	**0.001**
**Using RBCT location data**	**From initial proactive cull**						
	(a) baseline herds	-4.1%	-21.6%	17.3%	0.68	**1.74**	**0.003**
	(b) baseline cattle	-8.6%	-29.1%	17.8%	0.49	**2.20**	**<0.001**
	(c) number of herd tests	-4.4%	-19.4%	13.4%	0.60	**1.50**	**0.028**
	(d) number of cattle tested	-7.2%	-27.5%	18.9%	0.56	**2.14**	**<0.001**
	**From first follow-up**						
	(a) baseline herds	-4.5%	-23.5%	19.2%	0.68	**1.67**	**0.007**
	(b) baseline cattle	-9.6%	-31.5%	19.3%	0.47	**2.12**	**<0.001**
	(c) number of herd tests	-5.5%	-21.8%	14.3%	0.56	**1.45**	**0.039**
	(d) number of cattle tested	-8.2%	-29.9%	20.2%	0.53	**2.05**	**<0.001**

* The overdispersion factor was estimated as the square-root of the deviance divided by the degrees of freedom

**Reported confidence intervals are adjusted for the overdispersion factor (if it was >1)

9. Interpretation of analyses from different time periods

The main text presents analyses from two time periods, one dating from the completion of the initial cull (which shows statistically significant effects both inside and outside trial areas), and one dating from completion of the first follow-up cull (which shows a significant effect inside, but a non-significant trend outside). The reasons for considering these two time periods, and their implications for the interpretation of our findings, merit further comment.

We used incidence from the date of the initial cull as our primary analysis, mainly because this measure is the most relevant to policy: the effects detected reflect what one could expect to achieve from a proactive culling policy implemented on the timescale measured. As described briefly in the main text, there were two reasons for performing secondary analyses which excluded data from before the first follow-up cull. First, this excluded breakdowns that might have originated prior to the onset of culling, even though (given annual testing) they were not detected until after culling had begun. Such breakdowns would lead to under-estimation of culling-induced effects on incidence in the first year – essentially this would bias estimates of increases or reductions toward no effect. Our findings of statistically significant effects dating from completion of the initial cull, both inside and outside trial areas, therefore indicate the strength of both positive and negative effects of culling.

An additional reason for performing the secondary analyses was that a more complete badger removal would have been achieved from the date of the first follow-up cull (see Section 1 above). This more complete cull would be expected to generate a greater reduction in cattle TB inside trial areas, and the results of the secondary analysis are indeed consistent with this prediction, albeit with a wider confidence interval due to the smaller dataset.

In contrast with the situation inside trial areas, however, the circumstances of incomplete badger removal that would have occurred between the initial cull and the first follow-up could be expected to *increase* any detrimental effects of culling, if such effects were caused by disruption of badger territorial organisation at artificially reduced population densities. The frequency of potentially infectious contacts between cattle and badgers will be related to both the density of badgers, and the ranging behaviour of those badgers. We have hypothesised[1] that, where densities are substantially reduced, contact rates will be reduced despite expanded ranging behaviour, but that smaller reductions in density will generate increased contact rates if (as observed) they are also accompanied by expanded ranging. In this scenario, we would expect detrimental effects to be particularly marked following the initial cull since densities were probably reduced to a lesser extent during this period. Our secondary analysis excluded this potentially important time period and this, along with the reduced sample size, helps to explain why the culling effect in 'neighbouring areas' was found to be weaker.

An important component of this argument is the time taken for badger culling to generate additional cases of TB in cattle. If, as hypothesised, these effects are caused by disruption of badgers' spatial organisation at reduced population densities, this process would entail three steps: (i) culling triggers expansion of badger ranging behaviour and consequently contact with additional cattle herds; (ii) contact leads to infection in cattle; and (iii) infected cattle become responsive to the tuberculin test. Behavioural data show that local reductions in badger density cause badgers to alter their ranging behaviour within a few days or weeks[11-13], suggesting that step (i) could occur rapidly. The time taken for step (ii) to occur is unknown. Step (iii), the acquisition of responsiveness to the tuberculin test following exposure, takes approximately three weeks (personal communication from C. Howard, Institute for Animal Health, cited in Le Fevre (2005)[14]. Hence, if badgers can infect susceptible cattle rapidly on contact,

increased cattle incidence would be detectable 2-3 months after badger culling. Thus, breakdowns initiated following the initial cull should be detectable well before completion of the first follow-up cull about a year later.

This scenario assumes that detrimental effects of culling reflect greater contact between badgers and cattle as a consequence of expanded badger movement. An alternative scenario is that expanded movement might lead to greater transmission of infection among badgers, with consequently higher prevalence, ultimately causing greater transmission to cattle[15, 16]. In this scenario, detrimental effects of culling might indeed arise slowly because an additional (badger-badger) transmission stage is involved. The simpler scenario is, however, well supported by ecological data[1] and is more parsimonious.

10. Effects of widespread culling in previous studies

To facilitate comparison with our findings, we used published data[10-12] to estimate the effects of widespread badger culling on the incidence of cattle TB in previous studies. Table 19 and Table 20 present rates of cattle herd breakdowns in areas subjected to either intensive or localised culling ('removal' and 'reference' areas respectively) in two studies conducted in the Republic of Ireland[17, 18]. Data are presented only for the years when culling occurred, to ensure comparability with our study. Estimates presented in Table 19 of the reductions in cattle TB incidence associated with widespread culling are based on direct comparisons of incidence in reference and removal areas, without adjustment for any covariates. When log-linear regression models were fitted to the 'four areas' data (Table 19) adjusting for the (log-transformed) average number of herds at risk, the estimated reductions in TB incidence in intensively

culled areas were a 58% (95% CI: 41% to 70%) reduction from the start of culling and a 71% (95% CI: 55% to 81%) reduction from one year after the start of culling. There was no significant overdispersion in either model, and there was no improvement in the model with further adjustment for historic TB incidence.

The quantitative impact of badger culling in Britain's Thornbury area cannot meaningfully be compared with our results since the primary comparison was before and after culling[19]. During the culling period, the average incidence of cattle TB was higher in the removal area than in a nearby comparison area[19]. This difference almost certainly reflected background variation in historical incidence in the two areas. Although incidence later declined in the removal area, the published data preclude a comparison in the timeframe equivalent to the one used in the current study.

None of these studies investigated effects of culling on neighbouring areas. We would expect such effects to be weak in Ireland's 'four areas' trial[18] and at Thornbury[19], because culled areas were isolated from neighbouring cattle and badger populations by geographic barriers. The East Offaly study[17] compared data from herds ≥1.6km inside a single culling zone (roughly equivalent to our inner trial areas), with herds in an 8km wide 'control zone' immediately outside the culled area. By excluding data from the intervening (culled) 'buffer zone' and incorporating herds adjacent to the culled area in the large 'control zone', this study would fail to detect locally detrimental effects of nearby culling. However, since any such effects would occur inside the control zone, the beneficial effects of culling might have been somewhat over-estimated.

Table 19 Numbers of cattle herds experiencing confirmed TB breakdowns, and numbers of herds at risk, in the 'Four Areas Trial' carried out in the Republic of Ireland[10]. Data are presented only from years during which badger culling occurred.

	Cork		Donegal		Kilkenny		Monaghan	
Year	reference	remova	referenc	remova	reference	remova	reference	removal
1997-8	30/272	29/288	4/361	3/375	20/230	14/230	57/554	19/687
1998-9	45/271	22/285	5/349	6/375	28/222	4/230	62/565	32/701
1999-	33/271	11/282	5/343	3/375	25/214	6/229	42/565	24/681
2000-01	12/274	2/270	4/334	1/370	12/213	6/225	38/559	24/661
2001-02	13/269	3/259	18/320	1/365	16/206	4/214	29/545	13/644
Incidence								
All years	133/135	67/138	36/1707	14/186	101/108	34/112	228/2788	112/337
	9.80%	4.84%	2.11%	0.75%	9.31%	3.01%	8.18%	3.32%
Reductio		51%		64%		68%		59%
First year excluded	103/108	38/109	32/1346	11/148	81/855	20/898	171/2234	93/2687
	9.49%	3.47%	2.38%	0.74%	9.47%	2.23%	7.65%	3.46%
Reductio		63%		69%		76%		55%

Table 20 Numbers of individual cattle showing evidence of TB exposure, and numbers of cattle tested, in the East Offaly study carried out in the Republic of Ireland[11]. Data are presented only from years during which badger culling occurred.

Year	reference	removal
1989	982/294,088	362/103,032
1990	904/286,425	299/103,332
1991	979/218,813	194/72,202
1992	594/234,888	89/65,803
1993	404/212,382	54/67,086
1994	443/210,339	54/68,527
Incidence		
All years	4306/1,456,935	1052/479,982
	0.296%	0.219%
Reduction		26%
First year excluded	3324/1,162,847	690/376,950
	0.286%	0.183%
Reduction		36%

11. Literature cited
