# Supplementary material for: Absence of effects of widespread badger culling on tuberculosis in cattle
Source: Sci Rep. 2024 Jul 15;14:16326. doi: 10.1038/s41598-024-67160-0 (PMC11251155; doi:10.1038/s41598-024-67160-0)
Supplement: Supplementary file 5 — Supplementary Information 5. [file 41598_2024_67160_MOESM5_ESM.pdf]

Supplentary File 5. R code for: Absence of effects of widespread badger culling on tuberculosis in cattle

Torgerson P. R., Hartnack, S., Rasmussen, P., Lewis, F., Langton, T. E. S.

2024-03-06

```
library(glmmTMB)
library(MuMIn)
library(rstanarm)

## Loading required package: Rcpp

## This is rstanarm version 2.21.3

## - See https://mc-stan.org/rstanarm/articles/priors for changes to default priors
   !

## - Default priors may change, so it's safest to specify priors, even if
   equivalent to the defaults.

## - For execution on a local, multicore CPU with excess RAM we recommend calling

##   options(mc.cores = parallel::detectCores())

##
## Attaching package: 'rstanarm'

## The following object is masked from 'package:MuMIn':
##
##     loo

library(bridgesampling)
library(DHARMa)

## This is DHARMa 0.4.5. For overview type '?DHARMa'. For recent changes, type news
   (package = 'DHARMa')

library(readxl)
library(ggplot2)

#Load the data

rbctconf <- read_excel("rbctconf.xlsx", sheet = "Sheet1")


#short piece of code to make sure the reference level
#of variable exactly aligns with the analysis in Nature, 2006
rbctconf$Triplet<-relevel(factor(rbctconf$Triplet), ref="J")
rbctconf$Treatment<-relevel(factor(rbctconf$Treatment), ref="Survey-only")
```

```
#Model from 2006 paper
rs1<-glm(Incidence~Treatment+Triplet+log(Hist3yr)+log(Baseline), poisson, data=
    rbctconf)

summary(rs1)

##
## Call:
## glm(formula = Incidence ~ Treatment + Triplet + log(Hist3yr) +
##     log(Baseline), family = poisson, data = rbctconf)
##
## Deviance Residuals:
##      Min        1Q    Median        3Q       Max
## -0.85321  -0.54680  -0.00241   0.47137   0.88688
##
## Coefficients:
##                     Estimate Std. Error z value Pr(>|z|)
## (Intercept)         -0.36167    0.93212  -0.388  0.69801
## TreatmentProactive -0.20661    0.07287  -2.835  0.00458 **
## TripletA            -0.26894    0.23410  -1.149  0.25064
## TripletB             0.15640    0.16604   0.942  0.34621
## TripletC             0.43196    0.16141   2.676  0.00745 **
## TripletD            -0.30400    0.19043  -1.596  0.11041
## TripletE            -0.02578    0.17511  -0.147  0.88298
## TripletF            -0.15938    0.18547  -0.859  0.39017
## TripletG             0.48182    0.19488   2.472  0.01342 *
## TripletH            -0.24769    0.18918  -1.309  0.19044
## TripletI            -0.50242    0.19730  -2.546  0.01088 *
## log(Hist3yr)         1.24144    0.21255   5.841  5.2e-09 ***
## log(Baseline)        0.04678    0.24805   0.189  0.85042
## ---
## Signif. codes:
## 0 '***' 0.001 '**' 0.01 '*' 0.05 '.' 0.1 ' ' 1
##
## (Dispersion parameter for poisson family taken to be 1)
##
##     Null deviance: 167.3255  on 19  degrees of freedom
## Residual deviance:   5.6596  on  7  degrees of freedom
## AIC: 142.29
##
## Number of Fisher Scoring iterations: 4

AICc(rs1)

## [1] 202.9587

# This is model 1 in the text and gives the results in Table 1,
#and in the additional material of the 2006 paper.
# Post hoc analysis of model 1, and other (non quasi) models

plot(simulateResiduals(rs1))
```

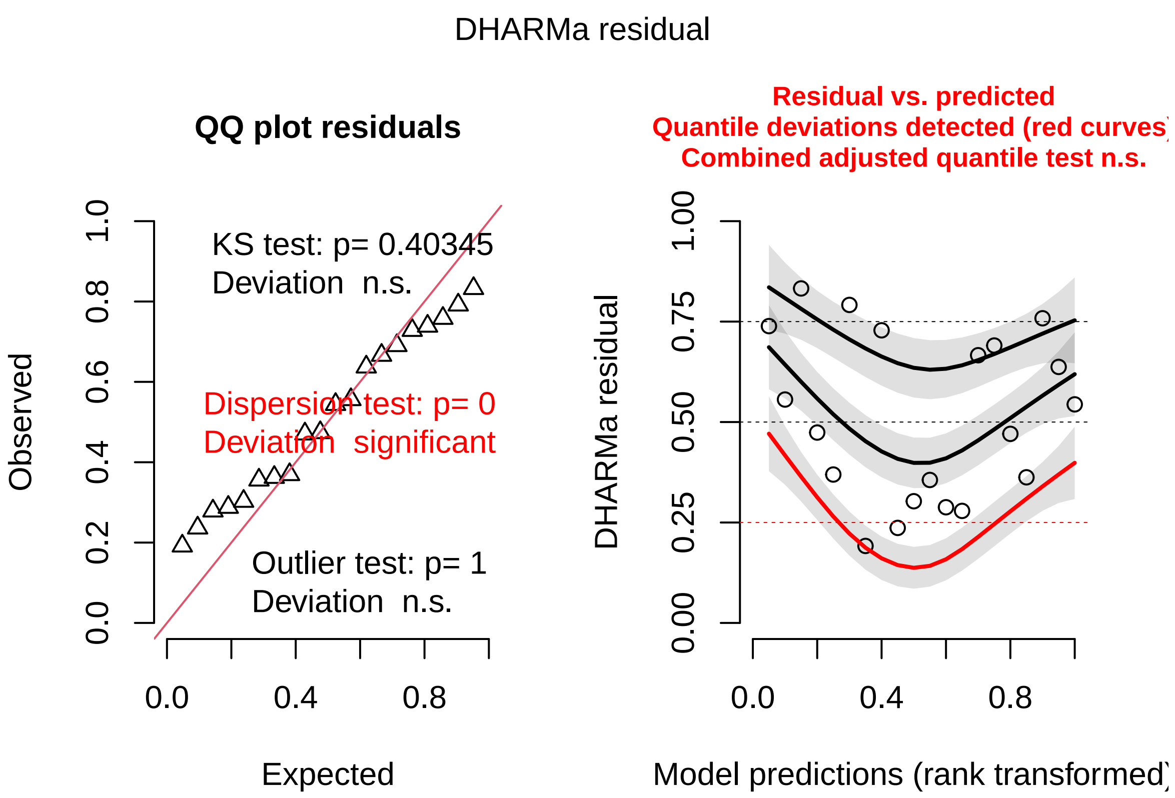


```
#This gives figure 1 in the text

# Model 2 in the text
rs2<-glm(Incidence~Treatment+Triplet+log(Hist3yr)+offset(log(Baseline)),
         quasipoisson, data=rbctconf)

summary(rs2)

##
## Call:
## glm(formula = Incidence ~ Treatment + Triplet + log(Hist3yr) +
##     offset(log(Baseline)), family = quasipoisson, data = rbctconf)
##
## Deviance Residuals:
##      Min        1Q    Median        3Q       Max
## -2.25722  -0.28151   0.01045   0.22610   2.90309
##
## Coefficients:
##                       Estimate Std. Error t value Pr(>|t|)
## (Intercept)         -3.4083259  0.8244394  -4.134  0.00328
## TreatmentProactive  -0.1464174  0.1169442  -1.252  0.24592
## TripletA             0.3275810  0.2851203   1.149  0.28378
## TripletB             0.2144111  0.2709480   0.791  0.45157
## TripletC             0.2447265  0.2525308   0.969  0.36088
## TripletD            -0.0039618  0.2843936  -0.014  0.98923
## TripletE             0.2051618  0.2675414   0.767  0.46520
## TripletF            -0.3955031  0.2831510  -1.397  0.20001
## TripletG            -0.0004596  0.2510366  -0.002  0.99858
## TripletH            -0.0478847  0.2924001  -0.164  0.87398
## TripletI            -0.2774756  0.3087597  -0.899  0.39507
## log(Hist3yr)         0.7407251  0.2634804   2.811  0.02280
##
```

```
## (Intercept)         **
## TreatmentProactive
## TripletA
## TripletB
## TripletC
## TripletD
## TripletE
## TripletF
## TripletG
## TripletH
## TripletI
## log(Hist3yr)        *
## ---
## Signif. codes:
## 0 '***' 0.001 '**' 0.01 '*' 0.05 '.' 0.1 ' ' 1
##
## (Dispersion parameter for quasipoisson family taken to be 2.686752)
##
##     Null deviance: 119.882  on 19  degrees of freedom
## Residual deviance:  20.128  on  8  degrees of freedom
## AIC: NA
##
## Number of Fisher Scoring iterations: 4
```

```
#Model 3 in the text
rs3<-glmmTMB(Incidence~Treatment+Triplet+log(Hist3yr)+log(Baseline),family=genpois,
    data=rbctconf)

summary(rs3)
```

```
##  Family: genpois  ( log )
## Formula:
## Incidence ~ Treatment + Triplet + log(Hist3yr) + log(Baseline)
## Data: rbctconf
##
##      AIC      BIC   logLik deviance df.resid
##    133.2    147.1    -52.6    105.2        6
##
##
## Dispersion parameter for genpois family (): 0.28
##
## Conditional model:
##                    Estimate Std. Error z value Pr(>|z|)
## (Intercept)        -0.34633    0.50217  -0.690  0.49041
## TreatmentProactive -0.20663    0.03861  -5.351 8.73e-08 ***
## TripletA           -0.27815    0.12361  -2.250  0.02443 *
## TripletB            0.14526    0.08835   1.644  0.10014
## TripletC            0.42270    0.08429   5.015 5.31e-07 ***
## TripletD           -0.31088    0.10055  -3.092  0.00199 **
## TripletE           -0.03455    0.09394  -0.368  0.71303
## TripletF           -0.16994    0.09619  -1.767  0.07728 .
## TripletG            0.47684    0.10351   4.607 4.09e-06 ***
## TripletH           -0.24178    0.10453  -2.313  0.02072 *
## TripletI           -0.52013    0.10479  -4.964 6.92e-07 ***
## log(Hist3yr)        1.24342    0.10867  11.442  < 2e-16 ***
## log(Baseline)       0.04384    0.13139   0.334  0.73865
## ---
```

```
## Signif. codes:
## 0 '***' 0.001 '**' 0.01 '*' 0.05 '.' 0.1 ' ' 1

AICc(rs3)

## [1] 217.1978

#Model 3a in the text
rs3a<-glmmTMB(Incidence~log(Hist3yr)+log(Baseline),family=genpois,  data=rbctconf)

summary(rs3a)

##  Family: genpois  ( log )
## Formula:          Incidence ~ log(Hist3yr) + log(Baseline)
## Data: rbctconf
##
##      AIC      BIC   logLik deviance df.resid
##    157.7    161.7    -74.9    149.7       16
##
##
## Dispersion parameter for genpois family (): 2.63
##
## Conditional model:
##               Estimate Std. Error z value Pr(>|z|)
## (Intercept)    -2.4021     0.9581  -2.507   0.0122 *
## log(Hist3yr)    0.9352     0.1938   4.825 1.40e-06 ***
## log(Baseline)   0.6569     0.1650   3.982 6.83e-05 ***
## ---
## Signif. codes:
## 0 '***' 0.001 '**' 0.01 '*' 0.05 '.' 0.1 ' ' 1

AICc(rs3a)

## [1] 160.3831

#Model 3b in the text
all.modelall2<-dredge(rs3,options(na.action = na.fail))

## Warning in eval(.expr_beta_arg): invalid value for 'beta' :
## the argument is taken to be "none"

## Fixed terms are "cond((Int))" and "disp((Int))"

final.models2=get.models(all.modelall2,cumsum(weight<=0.95))
table.final2=model.avg(final.models2)
summary(table.final2)

##
## Call:
## model.avg(object = final.models2)
##
## Component model call:
## glmmTMB(formula = Incidence ~ <16 unique rhs>, data =
##      rbctconf, family = genpois, ziformula = ~0,
##      dispformula = ~1)
##
## Component models:
```

```
##          df logLik   AICc  delta weight
## 12        4 -74.86 160.38  0.00   0.75
## 123       5 -74.22 162.72  2.33   0.23
## 2         3 -80.40 168.30  7.92   0.01
## 23        4 -79.50 169.67  9.28   0.01
## 1         3 -83.40 174.31 13.93   0.00
## 13        4 -83.04 176.74 16.36   0.00
## (Null)    2 -86.04 176.78 16.40   0.00
## 3         3 -85.46 178.42 18.04   0.00
## 234      13 -52.65 191.98 31.59   0.00
## 24       12 -62.09 192.75 32.36   0.00
## 124      13 -61.56 209.79 49.41   0.00
## 4        11 -79.26 213.52 53.14   0.00
## 14       12 -73.76 216.09 55.71   0.00
## 1234     14 -52.60 217.20 56.81   0.00
## 34       12 -78.50 225.58 65.20   0.00
## 134      13 -72.96 232.60 72.21   0.00
##
## Term codes:
## cond(log(Baseline))  cond(log(Hist3yr))      cond(Treatment)
##                   1                   2                    3
##       cond(Triplet)
##                   4
##
## Model-averaged coefficients:
## (full average)
##                         Estimate Std. Error Adjusted SE
## cond((Int))           -2.309e+00  1.057e+00   1.129e+00
## cond(log(Baseline))    6.387e-01  1.887e-01   2.005e-01
## cond(log(Hist3yr))     9.379e-01  1.976e-01   2.135e-01
## cond(TreatmentProactive) -2.967e-02  7.444e-02   7.768e-02
## cond(TripletA)        -4.954e-08  1.290e-04   1.327e-04
## cond(TripletB)         2.638e-08  7.881e-05   8.424e-05
## cond(TripletC)         7.907e-08  1.954e-04   1.973e-04
## cond(TripletD)        -5.343e-08  1.376e-04   1.410e-04
## cond(TripletE)        -4.975e-09  4.809e-05   5.629e-05
## cond(TripletF)        -2.069e-08  7.205e-05   7.837e-05
## cond(TripletG)         8.535e-08  2.097e-04   2.115e-04
## cond(TripletH)        -4.401e-08  1.170e-04   1.212e-04
## cond(TripletI)        -9.105e-08  2.253e-04   2.279e-04
##                       z value Pr(>|z|)
## cond((Int))             2.045  0.04081 *
## cond(log(Baseline))     3.186  0.00144 **
## cond(log(Hist3yr))      4.394 1.11e-05 ***
## cond(TreatmentProactive)  0.382  0.70253
## cond(TripletA)          0.000  0.99970
## cond(TripletB)          0.000  0.99975
## cond(TripletC)          0.000  0.99968
## cond(TripletD)          0.000  0.99970
## cond(TripletE)          0.000  0.99993
## cond(TripletF)          0.000  0.99979
## cond(TripletG)          0.000  0.99968
## cond(TripletH)          0.000  0.99971
## cond(TripletI)          0.000  0.99968
##
## (conditional average)
```

```
##                            Estimate Std. Error Adjusted SE
## cond((Int))               -2.30876    1.05703     1.12872
## cond(log(Baseline))        0.65281    0.16479     0.17843
## cond(log(Hist3yr))         0.93906    0.19498     0.21109
## cond(TreatmentProactive)  -0.12388    0.10708     0.11631
## cond(TripletA)            -0.28666    0.11887     0.14039
## cond(TripletB)             0.15263    0.11245     0.13330
## cond(TripletC)             0.45750    0.10783     0.12638
## cond(TripletD)            -0.30914    0.11808     0.13967
## cond(TripletE)            -0.02878    0.11204     0.13231
## cond(TripletF)            -0.11972    0.12531     0.14560
## cond(TripletG)             0.49384    0.10305     0.12231
## cond(TripletH)            -0.25465    0.11972     0.14212
## cond(TripletI)            -0.52681    0.12756     0.15145
##                           z value Pr(>|z|)
## cond((Int))                 2.045 0.040809 *
## cond(log(Baseline))         3.659 0.000254 ***
## cond(log(Hist3yr))          4.449  8.6e-06 ***
## cond(TreatmentProactive)    1.065 0.286852
## cond(TripletA)              2.042 0.041169 *
## cond(TripletB)              1.145 0.252202
## cond(TripletC)              3.620 0.000295 ***
## cond(TripletD)              2.213 0.026877 *
## cond(TripletE)              0.218 0.827788
## cond(TripletF)              0.822 0.410930
## cond(TripletG)              4.038  5.4e-05 ***
## cond(TripletH)              1.792 0.073179 .
## cond(TripletI)              3.479 0.000504 ***
## ---
## Signif. codes:
## 0 '***' 0.001 '**' 0.01 '*' 0.05 '.' 0.1 ' ' 1
```

```
model.avg(object = final.models2)
```

```
##
## Call:
## model.avg(object = final.models2)
##
## Component models:
## '12'      '123'     '2'       '23'      '1'       '13'
## '(Null)'  '3'       '234'     '24'      '124'     '4'
## '14'      '1234'    '34'      '134'
##
## Coefficients:
##        cond((Int)) cond(log(Baseline)) cond(log(Hist3yr))
## full     -2.308765           0.6386521          0.9379255
## subset   -2.308765           0.6528074          0.9390614
##        cond(TreatmentProactive) cond(TripletA)
## full                -0.02966757  -4.954329e-08
## subset              -0.12387556  -2.866606e-01
##        cond(TripletB) cond(TripletC) cond(TripletD)
## full     2.637875e-08   7.906988e-08  -5.342789e-08
## subset   1.526291e-01   4.575033e-01  -3.091371e-01
##        cond(TripletE) cond(TripletF) cond(TripletG)
## full    -4.974584e-09  -2.069192e-08   8.534944e-08
## subset  -2.878326e-02  -1.197248e-01   4.938373e-01
##        cond(TripletH) cond(TripletI)
```

```
## full      -4.401043e-08   -9.104859e-08
## subset   -2.546472e-01   -5.268129e-01

#Model 4 in the text
rs4<-glmmTMB(Incidence~Treatment+Triplet+
                  log(Hist3yr)+offset(log(hdyrsrisk)), family=genpois,data=rbctconf)
summary(rs4)

##  Family: genpois  ( log )
## Formula:
## Incidence ~ Treatment + Triplet + log(Hist3yr) + offset(log(hdyrsrisk))
## Data: rbctconf
##
##      AIC      BIC   logLik deviance df.resid
##    156.7    169.7    -65.4    130.7        7
##
##
## Dispersion parameter for genpois family (): 1.05
##
## Conditional model:
##                    Estimate Std. Error z value Pr(>|z|)
## (Intercept)        -4.46387    0.51442  -8.677  < 2e-16 ***
## TreatmentProactive -0.14686    0.07310  -2.009 0.044550 *
## TripletA           -0.33736    0.17784  -1.897 0.057827 .
## TripletB           -0.63445    0.16898  -3.755 0.000174 ***
## TripletC           -0.46422    0.15755  -2.946 0.003215 **
## TripletD            0.05311    0.17742   0.299 0.764677
## TripletE           -0.40196    0.16705  -2.406 0.016120 *
## TripletF           -0.97294    0.17656  -5.510 3.58e-08 ***
## TripletG           -0.51586    0.15667  -3.293 0.000992 ***
## TripletH           -0.54741    0.18653  -2.935 0.003339 **
## TripletI           -0.28745    0.19257  -1.493 0.135511
## log(Hist3yr)        0.74027    0.16433   4.505 6.64e-06 ***
## ---
## Signif. codes:
## 0 '***' 0.001 '**' 0.01 '*' 0.05 '.' 0.1 ' ' 1

AICc(rs4)

## [1] 217.404

#Model 4a
rs4a<-glmmTMB(Incidence~log(Hist3yr)+offset(log(hdyrsrisk)), family=genpois,data=
    rbctconf)

summary(rs4a)

##  Family: genpois  ( log )
## Formula:
## Incidence ~ log(Hist3yr) + offset(log(hdyrsrisk))
## Data: rbctconf
##
##      AIC      BIC   logLik deviance df.resid
##    166.6    169.6    -80.3    160.6       17
##
##
## Dispersion parameter for genpois family (): 3.96
```

```
##
## Conditional model:
##              Estimate Std. Error z value Pr(>|z|)
## (Intercept)   -4.6754     0.6689  -6.989 2.76e-12 ***
## log(Hist3yr)   0.6399     0.2025   3.161  0.00157 **
## ---
## Signif. codes:
## 0 '***' 0.001 '**' 0.01 '*' 0.05 '.' 0.1 ' ' 1
```

```
AICc(rs4a)
```

```
## [1] 168.1069
```

```
#Model 4b
all.modelall2<-dredge(rs4,options(na.action = na.fail))
```

```
## Warning in eval(.expr_beta_arg): invalid value for 'beta' :
## the argument is taken to be "none"

## Fixed terms are "cond((Int))" and "disp((Int))"
```

```
final.models2=get.models(all.modelall2,cumsum(weight<=0.95))
table.final2=model.avg(final.models2)
summary(table.final2)
```

```
##
## Call:
## model.avg(object = final.models2)
##
## Component model call:
## glmmTMB(formula = Incidence ~ <16 unique rhs>, data =
##      rbctconf, family = genpois, ziformula = ~0,
##      dispformula = ~1)
##
## Component models:
##         df logLik   AICc delta weight
## 12       3 -80.30 168.11  0.00   0.37
## 1        3 -80.40 168.30  0.20   0.33
## 13       4 -79.50 169.67  1.56   0.17
## 123      4 -80.02 170.71  2.61   0.10
## 2        2 -84.81 174.33  6.22   0.02
## 23       3 -84.33 176.16  8.05   0.01
## (Null)   2 -86.04 176.78  8.67   0.00
## 3        3 -85.46 178.42 10.32   0.00
## 134     13 -52.65 191.98 23.87   0.00
## 14      12 -62.09 192.75 24.64   0.00
## 24      11 -73.84 202.69 34.58   0.00
## 124     12 -67.26 203.09 34.98   0.00
## 4       11 -79.26 213.52 45.41   0.00
## 234     12 -73.01 214.60 46.49   0.00
## 1234    13 -65.37 217.40 49.30   0.00
## 34      12 -78.50 225.58 57.47   0.00
##
## Term codes:
##           cond(log(Hist3yr)) cond(offset(log(hdyrsrisk)))
##                            1                            2
##              cond(Treatment)                cond(Triplet)
```

```
##                                3                               4
##
## Model-averaged coefficients:
## (full average)
##                          Estimate Std. Error Adjusted SE
## cond((Int))             -1.897e+00  2.760e+00   2.777e+00
## cond(log(Hist3yr))       7.920e-01  3.201e-01   3.336e-01
## cond(TreatmentProactive) -4.304e-02  1.023e-01   1.065e-01
## cond(TripletA)          -1.166e-06  6.266e-04   6.447e-04
## cond(TripletB)           6.099e-07  3.891e-04   4.153e-04
## cond(TripletC)           1.847e-06  9.492e-04   9.586e-04
## cond(TripletD)          -1.250e-06  6.681e-04   6.851e-04
## cond(TripletE)          -1.235e-07  2.399e-04   2.792e-04
## cond(TripletF)          -5.047e-07  3.745e-04   4.037e-04
## cond(TripletG)           1.993e-06  1.019e-03   1.028e-03
## cond(TripletH)          -1.044e-06  5.730e-04   5.937e-04
## cond(TripletI)          -2.142e-06  1.093e-03   1.105e-03
##                          z value Pr(>|z|)
## cond((Int))                0.683   0.4946
## cond(log(Hist3yr))         2.374   0.0176 *
## cond(TreatmentProactive)   0.404   0.6860
## cond(TripletA)             0.002   0.9986
## cond(TripletB)             0.001   0.9988
## cond(TripletC)             0.002   0.9985
## cond(TripletD)             0.002   0.9985
## cond(TripletE)             0.000   0.9996
## cond(TripletF)             0.001   0.9990
## cond(TripletG)             0.002   0.9985
## cond(TripletH)             0.002   0.9986
## cond(TripletI)             0.002   0.9985
##
## (conditional average)
##                          Estimate Std. Error Adjusted SE
## cond((Int))               -1.8968     2.7600      2.7774
## cond(log(Hist3yr))         0.8163     0.2928      0.3079
## cond(TreatmentProactive)  -0.1552     0.1426      0.1532
## cond(TripletA)            -0.2860     0.1204      0.1419
## cond(TripletB)             0.1496     0.1214      0.1411
## cond(TripletC)             0.4530     0.1254      0.1418
## cond(TripletD)            -0.3066     0.1244      0.1452
## cond(TripletE)            -0.0303     0.1148      0.1349
## cond(TripletF)            -0.1238     0.1381      0.1570
## cond(TripletG)             0.4886     0.1267      0.1430
## cond(TripletH)            -0.2560     0.1222      0.1445
## cond(TripletI)            -0.5254     0.1301      0.1538
##                          z value Pr(>|z|)
## cond((Int))                0.683 0.494633
## cond(log(Hist3yr))         2.651 0.008027 **
## cond(TreatmentProactive)   1.013 0.310883
## cond(TripletA)             2.016 0.043838 *
## cond(TripletB)             1.060 0.289245
## cond(TripletC)             3.194 0.001402 **
## cond(TripletD)             2.111 0.034774 *
## cond(TripletE)             0.225 0.822315
## cond(TripletF)             0.788 0.430434
## cond(TripletG)             3.417 0.000634 ***
```

```
## cond(TripletH)              1.772 0.076379 .
## cond(TripletI)              3.415 0.000638 ***
## ---
## Signif. codes:
## 0 '***' 0.001 '**' 0.01 '*' 0.05 '.' 0.1 ' ' 1

model.avg(object = final.models2)

##
## Call:
## model.avg(object = final.models2)
##
## Component models:
## '12'      '1'      '13'     '123'    '2'      '23'
## '(Null)' '3'      '134'    '14'     '24'     '124'
## '4'      '234'    '1234'   '34'
##
## Coefficients:
##         cond((Int)) cond(log(Hist3yr))
## full      -1.896854          0.7919597
## subset    -1.896854          0.8163398
##         cond(TreatmentProactive) cond(TripletA)
## full                 -0.04303815  -1.166232e-06
## subset               -0.15520178  -2.859893e-01
##         cond(TripletB) cond(TripletC) cond(TripletD)
## full      6.099495e-07   1.847351e-06  -1.250216e-06
## subset    1.495749e-01   4.530166e-01  -3.065841e-01
##         cond(TripletE) cond(TripletF) cond(TripletG)
## full     -1.235489e-07  -5.046909e-07   1.992651e-06
## subset   -3.029727e-02  -1.237628e-01   4.886478e-01
##         cond(TripletH) cond(TripletI)
## full     -1.044149e-06  -2.142371e-06
## subset   -2.560515e-01  -5.253629e-01

#Model 4c in the text
rs4c<-glmmTMB(Incidence~Treatment+Triplet+log(Hist3yr)+log(hdyrsrisk), family=
    genpois,data=rbctconf)

summary(rs4c)

##  Family: genpois  ( log )
## Formula:
## Incidence ~ Treatment + Triplet + log(Hist3yr) + log(hdyrsrisk)
## Data: rbctconf
##
##      AIC      BIC   logLik deviance df.resid
##    133.2    147.1    -52.6    105.2        6
##
##
## Dispersion parameter for genpois family (): 0.28
##
## Conditional model:
##                     Estimate Std. Error z value Pr(>|z|)
## (Intercept)         -0.39270    0.62459  -0.629 0.529523
## TreatmentProactive -0.20663    0.03861  -5.351 8.74e-08 ***
## TripletA            -0.30730    0.09189  -3.344 0.000825 ***
## TripletB             0.10805    0.13476   0.802 0.422653
```

```
## TripletC             0.39164     0.14236    2.751  0.005940 **
## TripletD            -0.30837     0.10398   -2.966  0.003020 **
## TripletE            -0.06112     0.09613   -0.636  0.524907
## TripletF            -0.19525     0.14068   -1.388  0.165178
## TripletG             0.45426     0.15540    2.923  0.003465 **
## TripletH            -0.26343     0.09355   -2.816  0.004861 **
## TripletI            -0.52058     0.10436   -4.988  6.10e-07 ***
## log(Hist3yr)         1.24342     0.10868   11.441   < 2e-16 ***
## log(hdyrsrisk)       0.04384     0.13139    0.334  0.738653
## ---
## Signif. codes:
## 0 '***' 0.001 '**' 0.01 '*' 0.05 '.' 0.1 ' ' 1

AICc(rs4c)

## [1] 217.1978

#Model 4d in the text
rs4d<-glmmTMB(Incidence~log(Hist3yr)+log(hdyrsrisk), family=genpois,
              data=rbctconf)

summary(rs4d)

##  Family: genpois  ( log )
## Formula:          Incidence ~ log(Hist3yr) + log(hdyrsrisk)
## Data: rbctconf
##
##      AIC      BIC   logLik deviance df.resid
##    151.5    155.5    -71.8    143.5       16
##
##
## Dispersion parameter for genpois family (): 1.89
##
## Conditional model:
##                Estimate Std. Error z value Pr(>|z|)
## (Intercept)    -2.13664    0.71509  -2.988  0.00281 **
## log(Hist3yr)    0.82669    0.16096   5.136 2.81e-07 ***
## log(hdyrsrisk)  0.51201    0.09616   5.325 1.01e-07 ***
## ---
## Signif. codes:
## 0 '***' 0.001 '**' 0.01 '*' 0.05 '.' 0.1 ' ' 1

AICc(rs4d)

## [1] 154.2039

#Model 4e in the text
all.modelall2<-dredge(rs4c,options(na.action = na.fail))

## Warning in eval(.expr_beta_arg): invalid value for 'beta' :
## the argument is taken to be "none"

## Fixed terms are "cond((Int))" and "disp((Int))"

final.models2=get.models(all.modelall2,cumsum(weight<=0.95))
table.final2=model.avg(final.models2)
summary(table.final2)
```

```
##
## Call:
## model.avg(object = final.models2)
##
## Component model call:
## glmmTMB(formula = Incidence ~ <16 unique rhs>, data =
##      rbctconf, family = genpois, ziformula = ~0,
##      dispformula = ~1)
##
## Component models:
##        df logLik   AICc delta weight
## 12      4 -71.77 154.20  0.00    0.7
## 123     5 -70.79 155.87  1.66    0.3
## 2       3 -80.40 168.30 14.10    0.0
## 23      4 -79.50 169.67 15.46    0.0
## 1       3 -81.50 170.50 16.29    0.0
## 13      4 -80.88 172.42 18.22    0.0
## (Null)  2 -86.04 176.78 22.58    0.0
## 3       3 -85.46 178.42 24.22    0.0
## 234    13 -52.65 191.98 37.77    0.0
## 24     12 -62.09 192.75 38.54    0.0
## 124    13 -61.56 209.79 55.59    0.0
## 4      11 -79.26 213.52 59.31    0.0
## 14     12 -73.76 216.09 61.89    0.0
## 1234   14 -52.60 217.20 62.99    0.0
## 34     12 -78.50 225.58 71.38    0.0
## 134    13 -72.96 232.60 78.39    0.0
##
## Term codes:
## cond(log(hdyrsrisk))   cond(log(Hist3yr))
##                    1                    2
##      cond(Treatment)        cond(Triplet)
##                    3                    4
##
## Model-averaged coefficients:
## (full average)
##                           Estimate Std. Error Adjusted SE
## cond((Int))             -2.105e+00  7.143e-01   7.726e-01
## cond(log(hdyrsrisk))     5.085e-01  9.632e-02   1.041e-01
## cond(log(Hist3yr))       8.293e-01  1.603e-01   1.735e-01
## cond(TreatmentProactive) -3.851e-02  7.588e-02   7.867e-02
## cond(TripletA)          -2.105e-09  2.659e-05   2.735e-05
## cond(TripletB)           1.121e-09  1.624e-05   1.736e-05
## cond(TripletC)           3.359e-09  4.028e-05   4.067e-05
## cond(TripletD)          -2.270e-09  2.836e-05   2.907e-05
## cond(TripletE)          -2.114e-10  9.912e-06   1.160e-05
## cond(TripletF)          -8.791e-10  1.485e-05   1.615e-05
## cond(TripletG)           3.626e-09  4.323e-05   4.359e-05
## cond(TripletH)          -1.870e-09  2.411e-05   2.499e-05
## cond(TripletI)          -3.868e-09  4.644e-05   4.697e-05
##                         z value Pr(>|z|)
## cond((Int))               2.724  0.00645 **
## cond(log(hdyrsrisk))      4.884  1.0e-06 ***
## cond(log(Hist3yr))        4.779  1.8e-06 ***
## cond(TreatmentProactive)  0.490  0.62445
## cond(TripletA)            0.000  0.99994
```

```
## cond(TripletB)             0.000  0.99995
## cond(TripletC)             0.000  0.99993
## cond(TripletD)             0.000  0.99994
## cond(TripletE)             0.000  0.99999
## cond(TripletF)             0.000  0.99996
## cond(TripletG)             0.000  0.99993
## cond(TripletH)             0.000  0.99994
## cond(TripletI)             0.000  0.99993
##
## (conditional average)
##                         Estimate Std. Error Adjusted SE
## cond((Int))             -2.10479    0.71435     0.77265
## cond(log(hdyrsrisk))     0.50902    0.09512     0.10302
## cond(log(Hist3yr))       0.82955    0.15970     0.17299
## cond(TreatmentProactive) -0.12687   0.08809     0.09579
## cond(TripletA)          -0.28667    0.11884     0.14037
## cond(TripletB)           0.15261    0.11247     0.13332
## cond(TripletC)           0.45749    0.10787     0.12642
## cond(TripletD)          -0.30914    0.11809     0.13967
## cond(TripletE)          -0.02880    0.11204     0.13231
## cond(TripletF)          -0.11974    0.12534     0.14563
## cond(TripletG)           0.49383    0.10309     0.12235
## cond(TripletH)          -0.25466    0.11972     0.14212
## cond(TripletI)          -0.52681    0.12756     0.15145
##                         z value Pr(>|z|)
## cond((Int))               2.724 0.006447 **
## cond(log(hdyrsrisk))      4.941 8.00e-07 ***
## cond(log(Hist3yr))        4.795 1.60e-06 ***
## cond(TreatmentProactive)  1.324 0.185362
## cond(TripletA)            2.042 0.041124 *
## cond(TripletB)            1.145 0.252334
## cond(TripletC)            3.619 0.000296 ***
## cond(TripletD)            2.213 0.026880 *
## cond(TripletE)            0.218 0.827715
## cond(TripletF)            0.822 0.410961
## cond(TripletG)            4.036 5.43e-05 ***
## cond(TripletH)            1.792 0.073166 .
## cond(TripletI)            3.479 0.000504 ***
## ---
## Signif. codes:
## 0 '***' 0.001 '**' 0.01 '*' 0.05 '.' 0.1 ' ' 1
```

```
model.avg(object = final.models2)
```

```
##
## Call:
## model.avg(object = final.models2)
##
## Component models:
## '12'     '123'    '2'      '23'     '1'      '13'
## '(Null)' '3'      '234'    '24'     '124'    '4'
## '14'     '1234'   '34'     '134'
##
## Coefficients:
##        cond((Int)) cond(log(hdyrsrisk)) cond(log(Hist3yr))
## full     -2.104793            0.5085481          0.8293050
## subset   -2.104793            0.5090170          0.8295464
```

```
##          cond(TreatmentProactive) cond(TripletA)
## full                  -0.03851175   -2.104788e-09
## subset                -0.12687190   -2.866743e-01
##          cond(TripletB) cond(TripletC) cond(TripletD)
## full       1.120488e-09   3.358923e-09  -2.269704e-09
## subset     1.526116e-01   4.574887e-01  -3.091360e-01
##          cond(TripletE) cond(TripletF) cond(TripletG)
## full      -2.114209e-10  -8.791171e-10   3.625719e-09
## subset    -2.879574e-02  -1.197367e-01   4.938267e-01
##          cond(TripletH) cond(TripletI)
## full      -1.869717e-09  -3.867909e-09
## subset    -2.546573e-01  -5.268131e-01

#post hoc analysis

plot(simulateResiduals(rs4d))
```

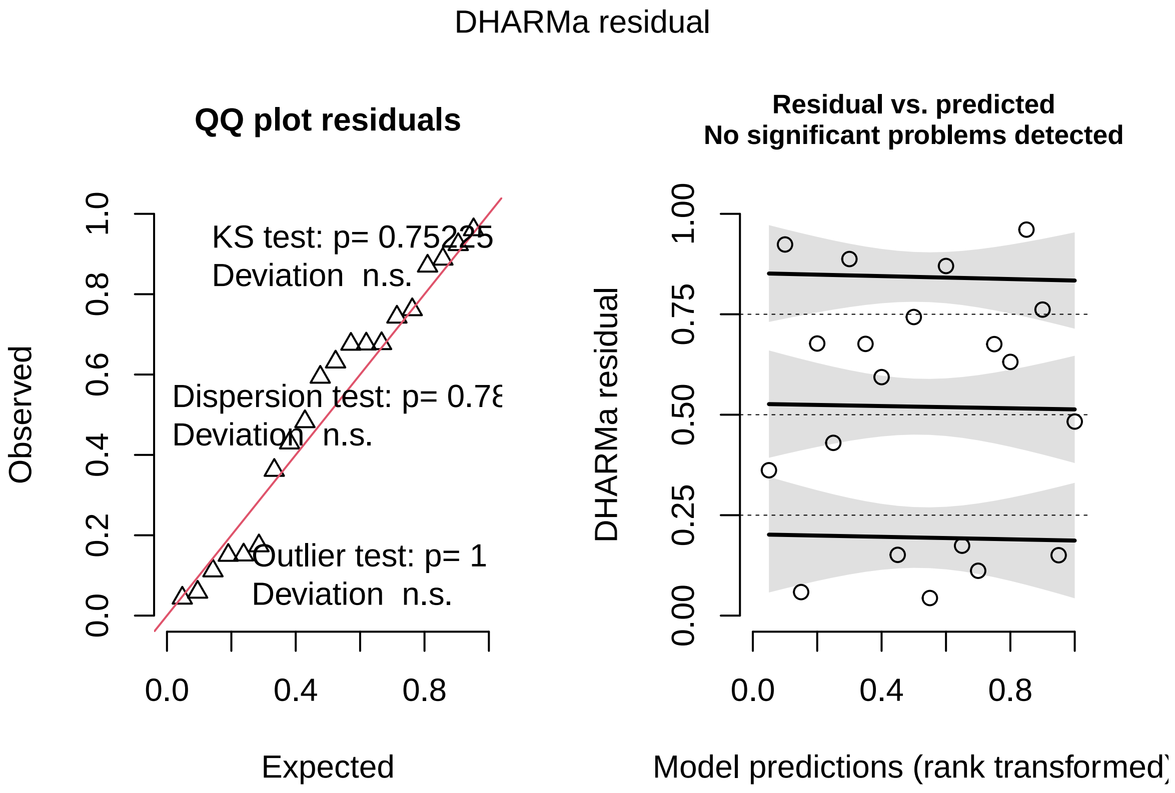


```
#This gives figure 2 in the text

#Model 5 in the text
rs5<-lm(Incidence/hdyrsrisk~Treatment+Triplet+log(Hist3yr), data=rbctconf)

summary(rs5)

##
## Call:
## lm(formula = Incidence/hdyrsrisk ~ Treatment + Triplet + log(Hist3yr),
##     data = rbctconf)
##
## Residuals:
##       Min        1Q    Median        3Q       Max
## -0.031619 -0.003847  0.000000  0.003847  0.031619
```

```
##
## Coefficients:
##                      Estimate Std. Error t value Pr(>|t|)
## (Intercept)         -0.012492   0.060197  -0.208   0.8408
## TreatmentProactive -0.004699   0.008993  -0.523   0.6154
## TripletA            -0.018472   0.021692  -0.852   0.4192
## TripletB            -0.043502   0.021956  -1.981   0.0829 .
## TripletC            -0.035699   0.020134  -1.773   0.1141
## TripletD             0.023001   0.020971   1.097   0.3046
## TripletE            -0.027780   0.020625  -1.347   0.2149
## TripletF            -0.059544   0.020119  -2.960   0.0182 *
## TripletG            -0.038922   0.020122  -1.934   0.0891 .
## TripletH            -0.030720   0.020140  -1.525   0.1657
## TripletI            -0.023273   0.020201  -1.152   0.2825
## log(Hist3yr)         0.038445   0.019078   2.015   0.0786 .
## ---
## Signif. codes:
## 0 '***' 0.001 '**' 0.01 '*' 0.05 '.' 0.1 ' ' 1
##
## Residual standard error: 0.02011 on 8 degrees of freedom
## Multiple R-squared:  0.8152, Adjusted R-squared:  0.5611
## F-statistic: 3.209 on 11 and 8 DF,  p-value: 0.05445

AICc(rs5)

## [1] -31.16628

#Model 5a in the text
rs5a<-lm(Incidence/hdyrsrisk~log(Hist3yr), data=rbctconf)

summary(rs5a)

##
## Call:
## lm(formula = Incidence/hdyrsrisk ~ log(Hist3yr), data = rbctconf)
##
## Residuals:
##       Min        1Q    Median        3Q       Max
## -0.038556 -0.024733 -0.001586  0.019927  0.047031
##
## Coefficients:
##              Estimate Std. Error t value Pr(>|t|)
## (Intercept)  -0.08077    0.06357  -1.271   0.2200
## log(Hist3yr)  0.05107    0.01976   2.584   0.0187 *
## ---
## Signif. codes:
## 0 '***' 0.001 '**' 0.01 '*' 0.05 '.' 0.1 ' ' 1
##
## Residual standard error: 0.02663 on 18 degrees of freedom
## Multiple R-squared:  0.2706, Adjusted R-squared:  0.2301
## F-statistic: 6.678 on 1 and 18 DF,  p-value: 0.01871

AICc(rs5a)

## [1] -82.87219

#Model 5b in the text
all.modelall2<-dredge(rs5,options(na.action = na.fail))
```

```
## Warning in eval(.expr_beta_arg): invalid value for 'beta' :
## the argument is taken to be "none"

## Fixed term is "(Intercept)"

final.models2=get.models(all.modelall2,cumsum(weight<=0.95))
table.final2=model.avg(final.models2)
summary(table.final2)

##
## Call:
## model.avg(object = final.models2)
##
## Component model call:
## lm(formula = Incidence/hdyrsrisk ~ <8 unique rhs>,
##      data = rbctconf)
##
## Component models:
##         df logLik    AICc delta weight
## 1        3  45.19 -82.87  0.00   0.69
## 12       4  45.27 -79.87  3.00   0.16
## (Null)   2  42.03 -79.36  3.52   0.12
## 2        3  42.10 -76.70  6.17   0.03
## 3       11  54.57 -54.14 28.73   0.00
## 13      12  58.58 -48.59 34.28   0.00
## 23      12  54.81 -41.05 41.82   0.00
## 123     13  58.92 -31.17 51.71   0.00
##
## Term codes:
## log(Hist3yr)    Treatment      Triplet
##            1            2            3
##
## Model-averaged coefficients:
## (full average)
##                     Estimate Std. Error Adjusted SE
## (Intercept)       -5.557e-02  8.314e-02   8.624e-02
## log(Hist3yr)       4.334e-02  2.587e-02   2.683e-02
## TreatmentProactive -8.738e-04  5.704e-03   6.092e-03
## TripletA          -1.293e-09  1.487e-05   1.683e-05
## TripletB          -1.140e-08  2.289e-05   2.421e-05
## TripletC          -1.600e-08  2.848e-05   2.954e-05
## TripletD           1.459e-08  2.672e-05   2.785e-05
## TripletE          -8.117e-09  1.915e-05   2.070e-05
## TripletF          -2.587e-08  4.221e-05   4.294e-05
## TripletG          -1.717e-08  3.003e-05   3.104e-05
## TripletH          -1.217e-08  2.361e-05   2.488e-05
## TripletI          -8.344e-09  1.933e-05   2.086e-05
##                    z value Pr(>|z|)
## (Intercept)          0.644    0.519
## log(Hist3yr)         1.615    0.106
## TreatmentProactive   0.143    0.886
## TripletA             0.000    1.000
## TripletB             0.000    1.000
## TripletC             0.001    1.000
## TripletD             0.001    1.000
## TripletE             0.000    1.000
## TripletF             0.001    1.000
```

```
## TripletG              0.001     1.000
## TripletH              0.000     1.000
## TripletI              0.000     1.000
##
## (conditional average)
##                    Estimate Std. Error Adjusted SE z value
## (Intercept)        -0.055570   0.083142    0.086238   0.644
## log(Hist3yr)        0.051062   0.019854    0.021299   2.397
## TreatmentProactive -0.004681   0.012509    0.013453   0.348
## TripletA           -0.003040   0.022597    0.025624   0.119
## TripletB           -0.026780   0.022668    0.025691   1.042
## TripletC           -0.037605   0.022191    0.025244   1.490
## TripletD            0.034293   0.022406    0.025445   1.348
## TripletE           -0.019076   0.022317    0.025361   0.752
## TripletF           -0.060792   0.022187    0.025240   2.409
## TripletG           -0.040343   0.022188    0.025241   1.598
## TripletH           -0.028598   0.022192    0.025245   1.133
## TripletI           -0.019610   0.022208    0.025260   0.776
##                    Pr(>|z|)
## (Intercept)          0.5193
## log(Hist3yr)         0.0165 *
## TreatmentProactive   0.7279
## TripletA             0.9056
## TripletB             0.2972
## TripletC             0.1363
## TripletD             0.1777
## TripletE             0.4519
## TripletF             0.0160 *
## TripletG             0.1100
## TripletH             0.2573
## TripletI             0.4376
## ---
## Signif. codes:
## 0 '***' 0.001 '**' 0.01 '*' 0.05 '.' 0.1 ' ' 1
```

```
model.avg(object = final.models2)
```

```
##
## Call:
## model.avg(object = final.models2)
##
## Component models:
## '1'      '12'      '(Null)' '2'      '3'      '13'
## '23'     '123'
##
## Coefficients:
##        (Intercept) log(Hist3yr) TreatmentProactive
## full   -0.05557001   0.04333949      -0.0008737708
## subset -0.05557001   0.05106212      -0.0046809081
##             TripletA      TripletB      TripletC
## full   -1.293408e-09 -1.139451e-08 -1.600073e-08
## subset -3.039795e-03 -2.677963e-02 -3.760525e-02
##            TripletD      TripletE      TripletF
## full   1.459159e-08 -8.116786e-09 -2.586643e-08
## subset 3.429345e-02 -1.907624e-02 -6.079183e-02
##             TripletG      TripletH      TripletI
## full   -1.716567e-08 -1.216806e-08 -8.343886e-09
```

```
## subset -4.034312e-02 -2.859763e-02 -1.960998e-02

#post hoc analysis

plot(simulateResiduals(rs5a))
```

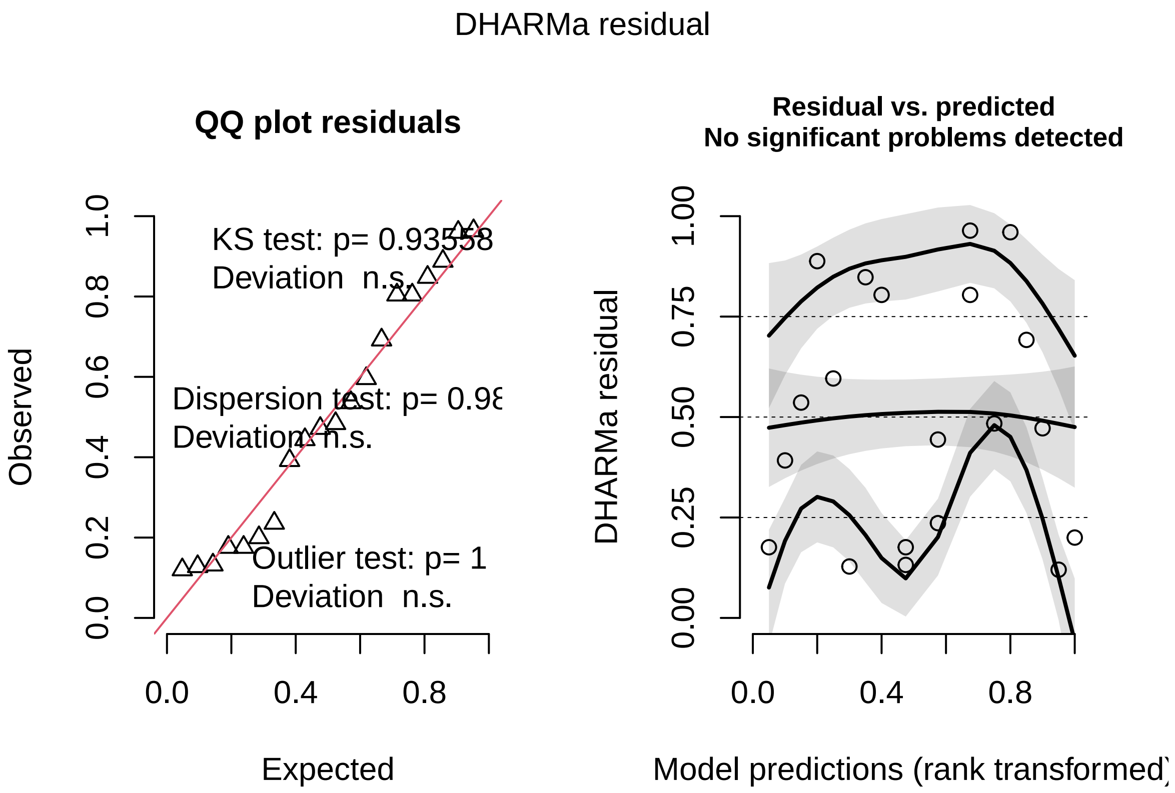


```
#This gives figure 3 in the text


#Model 6 in the text
perturb <- read_excel("perturb.xlsx")
perturb$Triplet<-relevel(factor(perturb$Triplet), ref="J")
perturb$Treatment<-relevel(factor(perturb$Treatment), ref="Survey-only")

rs6<-glm(Incidence~Treatment+Triplet+log(Hist3yr)+log(Baseline),
         poisson, data=perturb)

summary(rs6)

##
## Call:
## glm(formula = Incidence ~ Treatment + Triplet + log(Hist3yr) +
##     log(Baseline), family = poisson, data = perturb)
##
## Deviance Residuals:
##      Min        1Q    Median        3Q       Max
## -1.07254  -0.51256  -0.00993   0.45459   0.94001
##
## Coefficients:
##                    Estimate Std. Error z value Pr(>|z|)
## (Intercept)         1.87929    0.98260   1.913  0.05580 .
## TreatmentProactive  0.25310    0.10083   2.510  0.01207 *
```

```
## TripletA            -0.27471    0.26640  -1.031  0.30245
## TripletB             0.67142    0.17204   3.903 9.51e-05 ***
## TripletC             0.33289    0.18395   1.810  0.07034 .
## TripletD            -0.33151    0.28116  -1.179  0.23837
## TripletE             0.10470    0.20659   0.507  0.61229
## TripletF             0.12297    0.21294   0.577  0.56362
## TripletG             0.24100    0.20903   1.153  0.24894
## TripletH             0.31090    0.20272   1.534  0.12513
## TripletI            -0.56201    0.26726  -2.103  0.03548 *
## log(Hist3yr)         0.29362    0.09797   2.997  0.00273 **
## log(Baseline)        0.10807    0.22944   0.471  0.63762
## ---
## Signif. codes:
## 0 '***' 0.001 '**' 0.01 '*' 0.05 '.' 0.1 ' ' 1
##
## (Dispersion parameter for poisson family taken to be 1)
##
##     Null deviance: 108.3610  on 19  degrees of freedom
## Residual deviance:   7.4481  on  7  degrees of freedom
## AIC: 135.36
##
## Number of Fisher Scoring iterations: 4
```

```
AICc(rs6)
```

```
## [1] 196.0238
```

```
#Model 6a in the text
rs6a<-glmmTMB(Incidence~Treatment+Triplet+log(Hist3yr)+
              offset(log(hdyrsrisk)),family=genpois,data=perturb)

summary(rs6a)
```

```
##  Family: genpois  ( log )
## Formula:
## Incidence ~ Treatment + Triplet + log(Hist3yr) + offset(log(hdyrsrisk))
## Data: perturb
##
##      AIC      BIC   logLik deviance df.resid
##      150      163      -62      124        7
##
##
## Dispersion parameter for genpois family (): 1.11
##
## Conditional model:
##                     Estimate Std. Error z value Pr(>|z|)
## (Intercept)         -2.87216    0.28990  -9.907   <2e-16 ***
## TreatmentProactive   0.09493    0.09725   0.976   0.3290
## TripletA            -0.31860    0.22401  -1.422   0.1549
## TripletB            -0.13601    0.18320  -0.742   0.4578
## TripletC            -0.42375    0.19305  -2.195   0.0282 *
## TripletD             0.37845    0.23834   1.588   0.1123
## TripletE            -0.21437    0.20389  -1.051   0.2931
## TripletF            -0.46373    0.21996  -2.108   0.0350 *
## TripletG            -0.55056    0.20557  -2.678   0.0074 **
## TripletH             0.13117    0.19517   0.672   0.5015
## TripletI            -0.07469    0.24811  -0.301   0.7634
```

```
## log(Hist3yr)          0.11657     0.09243    1.261    0.2072
## ---
## Signif. codes:
## 0 '***' 0.001 '**' 0.01 '*' 0.05 '.' 0.1 ' ' 1
```

```
AICc(rs6a)
```

```
## [1] 210.7029
```

```
#Model 6b in the text
rs6b<-glmmTMB(Incidence~offset(log(hdyrsrisk)),family=genpois, data=perturb)
```

```
## Warning in (function (start, objective, gradient = NULL,
## hessian = NULL, : NA/NaN function evaluation
```

```
summary(rs6b)
```

```
##  Family: genpois  ( log )
## Formula:          Incidence ~ offset(log(hdyrsrisk))
## Data: perturb
##
##      AIC      BIC   logLik deviance df.resid
##    149.2    151.2    -72.6    145.2       18
##
##
## Dispersion parameter for genpois family (): 3.04
##
## Conditional model:
##             Estimate Std. Error z value Pr(>|z|)
## (Intercept) -2.74879    0.07308  -37.61   <2e-16 ***
## ---
## Signif. codes:
## 0 '***' 0.001 '**' 0.01 '*' 0.05 '.' 0.1 ' ' 1
```

```
AICc(rs6b)
```

```
## [1] 149.9424
```

```
#Model 6c
all.modelall2<-dredge(rs6a,options(na.action = na.fail))
```

```
## Warning in eval(.expr_beta_arg): invalid value for 'beta' :
## the argument is taken to be "none"
```

```
## Fixed terms are "cond((Int))" and "disp((Int))"
```

```
## Warning in (function (start, objective, gradient = NULL,
## hessian = NULL, : NA/NaN function evaluation
```

```
final.models2=get.models(all.modelall2,cumsum(weight<=0.95))
```

```
## Warning in (function (start, objective, gradient = NULL,
## hessian = NULL, : NA/NaN function evaluation
```

```
table.final2=model.avg(final.models2)
summary(table.final2)
```

```
##
## Call:
## model.avg(object = final.models2)
##
## Component model call:
## glmmTMB(formula = Incidence ~ <16 unique rhs>, data =
##      perturb, family = genpois, ziformula = ~0,
##      dispformula = ~1)
##
## Component models:
##          df logLik   AICc delta weight
## 2         2 -72.62 149.94  0.00   0.43
## 12        3 -71.47 150.45  0.50   0.34
## 23        3 -72.59 152.68  2.73   0.11
## 123       4 -71.03 152.73  2.78   0.11
## (Null)    2 -77.58 159.86  9.91   0.00
## 1         3 -76.32 160.15 10.21   0.00
## 13        4 -75.32 161.30 11.35   0.00
## 3         3 -77.37 162.23 12.29   0.00
## 24       11 -62.96 180.92 30.98   0.00
## 4        11 -63.36 181.72 31.78   0.00
## 14       12 -59.24 187.05 37.11   0.00
## 134      13 -51.23 189.13 39.19   0.00
## 34       12 -61.90 192.37 42.43   0.00
## 124      12 -62.49 193.55 43.61   0.00
## 234      12 -62.80 194.18 44.23   0.00
## 1234     13 -62.02 210.70 60.76   0.00
##
## Term codes:
##          cond(log(Hist3yr)) cond(offset(log(hdyrsrisk)))
##                           1                            2
##             cond(Treatment)                cond(Triplet)
##                           3                            4
##
## Model-averaged coefficients:
## (full average)
##                           Estimate Std. Error Adjusted SE
## cond((Int))             -2.907e+00  6.235e-01   6.294e-01
## cond(log(Hist3yr))       7.923e-02  1.172e-01   1.212e-01
## cond(TreatmentProactive)  1.896e-02  7.966e-02   8.416e-02
## cond(TripletA)          -2.967e-08  1.181e-04   1.280e-04
## cond(TripletB)           3.860e-08  1.987e-04   2.028e-04
## cond(TripletC)          -1.342e-08  1.655e-04   1.712e-04
## cond(TripletD)           2.585e-09  1.793e-04   1.872e-04
## cond(TripletE)          -9.108e-09  1.012e-04   1.112e-04
## cond(TripletF)          -4.993e-08  1.746e-04   1.814e-04
## cond(TripletG)          -3.941e-08  1.924e-04   1.977e-04
## cond(TripletH)           3.324e-08  1.226e-04   1.303e-04
## cond(TripletI)          -4.140e-08  1.783e-04   1.870e-04
##                         z value Pr(>|z|)
## cond((Int))               4.618  3.9e-06 ***
## cond(log(Hist3yr))        0.654    0.513
## cond(TreatmentProactive)  0.225    0.822
## cond(TripletA)            0.000    1.000
## cond(TripletB)            0.000    1.000
## cond(TripletC)            0.000    1.000
```

```
## cond(TripletD)              0.000     1.000
## cond(TripletE)              0.000     1.000
## cond(TripletF)              0.000     1.000
## cond(TripletG)              0.000     1.000
## cond(TripletH)              0.000     1.000
## cond(TripletI)              0.000     1.000
##
## (conditional average)
##                           Estimate Std. Error Adjusted SE
## cond((Int))               -2.90686    0.62352     0.62941
## cond(log(Hist3yr))         0.17592    0.11606     0.12490
## cond(TreatmentProactive)   0.08564    0.15150     0.16212
## cond(TripletA)            -0.20905    0.23359     0.26779
## cond(TripletB)             0.27200    0.45184     0.46461
## cond(TripletC)            -0.09455    0.42901     0.44454
## cond(TripletD)             0.01822    0.47564     0.49674
## cond(TripletE)            -0.06418    0.26081     0.28825
## cond(TripletF)            -0.35184    0.30159     0.32867
## cond(TripletG)            -0.27773    0.42853     0.44546
## cond(TripletH)             0.23428    0.22588     0.25437
## cond(TripletI)            -0.29172    0.37257     0.40159
##                           z value Pr(>|z|)
## cond((Int))                 4.618  3.9e-06 ***
## cond(log(Hist3yr))          1.408    0.159
## cond(TreatmentProactive)    0.528    0.597
## cond(TripletA)              0.781    0.435
## cond(TripletB)              0.585    0.558
## cond(TripletC)              0.213    0.832
## cond(TripletD)              0.037    0.971
## cond(TripletE)              0.223    0.824
## cond(TripletF)              1.070    0.284
## cond(TripletG)              0.623    0.533
## cond(TripletH)              0.921    0.357
## cond(TripletI)              0.726    0.468
## ---
## Signif. codes:
## 0 '***' 0.001 '**' 0.01 '*' 0.05 '.' 0.1 ' ' 1
```

```
model.avg(object = final.models2)
```

```
##
## Call:
## model.avg(object = final.models2)
##
## Component models:
## '2'      '12'     '23'     '123'    '(Null)' '1'
## '13'     '3'      '24'     '4'      '14'     '134'
## '34'     '124'    '234'    '1234'
##
## Coefficients:
##        cond((Int)) cond(log(Hist3yr))
## full     -2.906856         0.07923304
## subset   -2.906856         0.17591936
##        cond(TreatmentProactive) cond(TripletA)
## full                 0.01896063  -2.966526e-08
## subset               0.08564357  -2.090518e-01
##        cond(TripletB) cond(TripletC) cond(TripletD)
```

```
## full       3.859813e-08  -1.341715e-08   2.584808e-09
## subset     2.720019e-01  -9.455095e-02   1.821521e-02
##           cond(TripletE) cond(TripletF) cond(TripletG)
## full      -9.107516e-09  -4.992736e-08  -3.941153e-08
## subset    -6.418088e-02  -3.518393e-01  -2.777340e-01
##           cond(TripletH) cond(TripletI)
## full       3.324458e-08  -4.139683e-08
## subset     2.342754e-01  -2.917244e-01
```

```
#Model 7
rbct_allbreakdowns <- read_excel("rbct-allbreakdowns.xlsx")
rbct_allbreakdowns$Triplet<-relevel(factor(rbct_allbreakdowns$Triplet), ref="J")
rbct_allbreakdowns$Treatment<-relevel(factor(rbct_allbreakdowns$Treatment), ref="
    Survey-only")
rs7<-glm(Incidence~Treatment+Triplet+log(Hist3yr)+log(Baseline),
          quasipoisson, data=rbct_allbreakdowns)

summary(rs7)
```

```
##
## Call:
## glm(formula = Incidence ~ Treatment + Triplet + log(Hist3yr) +
##     log(Baseline), family = quasipoisson, data = rbct_allbreakdowns)
##
## Deviance Residuals:
##      Min        1Q    Median        3Q       Max
## -1.73868  -0.48053  -0.00895   0.50661   2.18503
##
## Coefficients:
##                    Estimate Std. Error t value Pr(>|t|)
## (Intercept)        -1.14735    1.19621  -0.959   0.3694
## TreatmentProactive -0.08281    0.08923  -0.928   0.3843
## TripletA            0.20783    0.25706   0.808   0.4454
## TripletB            0.16854    0.19550   0.862   0.4172
## TripletC            0.21106    0.19890   1.061   0.3239
## TripletD           -0.15656    0.22378  -0.700   0.5067
## TripletE            0.25219    0.19686   1.281   0.2410
## TripletF           -0.20472    0.21974  -0.932   0.3825
## TripletG           -0.14892    0.23113  -0.644   0.5399
## TripletH           -0.02507    0.21947  -0.114   0.9123
## TripletI           -0.19182    0.22665  -0.846   0.4253
## log(Hist3yr)        0.57475    0.22413   2.564   0.0373 *
## log(Baseline)       0.68024    0.30352   2.241   0.0600 .
## ---
## Signif. codes:
## 0 '***' 0.001 '**' 0.01 '*' 0.05 '.' 0.1 ' ' 1
##
## (Dispersion parameter for quasipoisson family taken to be 2.353964)
##
##     Null deviance: 168.79  on 19  degrees of freedom
## Residual deviance:  16.23  on  7  degrees of freedom
## AIC: NA
##
## Number of Fisher Scoring iterations: 4
```

```
#Model 7a
rs7a<-glmmTMB(round(Incidence/0.85)~Treatment+Triplet+log(Hist3yr)+log(hrdyrsrisk),
```

```
               family=genpois, data=rbct_allbreakdowns)

summary(rs7a)

##  Family: genpois  ( log )
## Formula:
## round(Incidence/0.85) ~ Treatment + Triplet + log(Hist3yr) +
##     log(hrdyrsrisk)
## Data: rbct_allbreakdowns
##
##      AIC      BIC   logLik deviance df.resid
##    169.3    183.3    -70.7    141.3        6
##
##
## Dispersion parameter for genpois family (): 0.979
##
## Conditional model:
##                     Estimate Std. Error z value Pr(>|z|)
## (Intercept)         -1.67851    0.87908  -1.909 0.056211 .
## TreatmentProactive  -0.08107    0.05308  -1.527 0.126666
## TripletA            -0.24879    0.12407  -2.005 0.044930 *
## TripletB            -0.41399    0.19594  -2.113 0.034618 *
## TripletC            -0.27470    0.20025  -1.372 0.170126
## TripletD            -0.12548    0.13654  -0.919 0.358109
## TripletE            -0.16442    0.14293  -1.150 0.249984
## TripletF            -0.59681    0.19668  -3.034 0.002410 **
## TripletG            -0.50722    0.20407  -2.485 0.012937 *
## TripletH            -0.36522    0.14731  -2.479 0.013164 *
## TripletI            -0.20438    0.13415  -1.524 0.127629
## log(Hist3yr)         0.57239    0.13317   4.298 1.72e-05 ***
## log(hrdyrsrisk)      0.67793    0.18083   3.749 0.000178 ***
## ---
## Signif. codes:
## 0 '***' 0.001 '**' 0.01 '*' 0.05 '.' 0.1 ' ' 1

AICc(rs7a)

## [1] 253.3444

#Model 7b
rs7b<-glmmTMB(round(Incidence/0.85)~log(Hist3yr)+log(hrdyrsrisk),
               family=genpois, data=rbct_allbreakdowns)

summary(rs7b)

##  Family: genpois  ( log )
## Formula:
## round(Incidence/0.85) ~ log(Hist3yr) + log(hrdyrsrisk)
## Data: rbct_allbreakdowns
##
##      AIC      BIC   logLik deviance df.resid
##    165.2    169.2    -78.6    157.2       16
##
##
## Dispersion parameter for genpois family (): 2.17
##
## Conditional model:
```

```
##                    Estimate Std. Error z value Pr(>|z|)
## (Intercept)        -0.54874    0.57630  -0.952    0.341
## log(Hist3yr)        0.63235    0.13815   4.577 4.71e-06 ***
## log(hrdyrsrisk)     0.41247    0.08293   4.974 6.56e-07 ***
## ---
## Signif. codes:
## 0 '***' 0.001 '**' 0.01 '*' 0.05 '.' 0.1 ' ' 1
```

```
AICc(rs7b)
```

```
## [1] 167.871
```

```
#Model 7c
all.modelall2<-dredge(rs7a,options(na.action = na.fail))
```

```
## Warning in eval(.expr_beta_arg): invalid value for 'beta' :
## the argument is taken to be "none"
```

```
## Fixed terms are "cond((Int))" and "disp((Int))"
```

```
final.models2=get.models(all.modelall2,cumsum(weight<=0.95))
table.final2=model.avg(final.models2)
summary(table.final2)
```

```
##
## Call:
## model.avg(object = final.models2)
##
## Component model call:
## glmmTMB(formula = round(Incidence/0.85) ~ <16 unique
##      rhs>, data = rbct_allbreakdowns, family = genpois,
##      ziformula = ~0, dispformula = ~1)
##
## Component models:
##         df logLik   AICc delta weight
## 12       4 -78.60 167.87  0.00   0.79
## 123      5 -78.12 170.52  2.65   0.21
## 2        3 -86.51 180.52 12.65   0.00
## 1        3 -86.70 180.91 13.04   0.00
## 23       4 -85.82 182.32 14.45   0.00
## 13       4 -86.29 183.26 15.38   0.00
## (Null)   2 -92.87 190.45 22.58   0.00
## 3        3 -92.20 191.90 24.03   0.00
## 14      12 -77.30 223.17 55.30   0.00
## 24      12 -77.99 224.54 56.67   0.00
## 124     13 -71.78 230.23 62.36   0.00
## 4       11 -87.95 230.91 63.04   0.00
## 134     13 -76.24 239.14 71.27   0.00
## 234     13 -77.18 241.04 73.17   0.00
## 34      12 -87.08 242.72 74.85   0.00
## 1234    14 -70.67 253.34 85.47   0.00
##
## Term codes:
##    cond(log(Hist3yr)) cond(log(hrdyrsrisk))
##                     1                     2
##       cond(Treatment)         cond(Triplet)
##                     3                     4
```

```
##
## Model-averaged coefficients:
## (full average)
##                         Estimate Std. Error Adjusted SE
## cond((Int))           -5.247e-01  5.854e-01   6.323e-01
## cond(log(Hist3yr))     6.295e-01  1.410e-01   1.522e-01
## cond(log(hrdyrsrisk))  4.115e-01  8.426e-02   9.097e-02
## cond(TreatmentProactive) -1.567e-02  4.563e-02   4.789e-02
## cond(TripletA)        -1.989e-13  2.676e-07   2.915e-07
## cond(TripletB)        -1.723e-13  5.586e-07   5.759e-07
## cond(TripletC)        -2.176e-14  6.184e-07   6.324e-07
## cond(TripletD)        -1.967e-13  3.314e-07   3.528e-07
## cond(TripletE)        -2.873e-14  3.585e-07   3.774e-07
## cond(TripletF)        -3.955e-13  6.845e-07   6.982e-07
## cond(TripletG)        -3.159e-13  6.629e-07   6.779e-07
## cond(TripletH)        -3.408e-13  4.724e-07   4.899e-07
## cond(TripletI)        -2.941e-13  3.544e-07   3.758e-07
##                       z value Pr(>|z|)
## cond((Int))             0.830    0.407
## cond(log(Hist3yr))      4.137 3.51e-05 ***
## cond(log(hrdyrsrisk))   4.523 6.10e-06 ***
## cond(TreatmentProactive)   0.327    0.743
## cond(TripletA)          0.000    1.000
## cond(TripletB)          0.000    1.000
## cond(TripletC)          0.000    1.000
## cond(TripletD)          0.000    1.000
## cond(TripletE)          0.000    1.000
## cond(TripletF)          0.000    1.000
## cond(TripletG)          0.000    1.000
## cond(TripletH)          0.000    1.000
## cond(TripletI)          0.000    1.000
##
## (conditional average)
##                       Estimate Std. Error Adjusted SE
## cond((Int))           -0.52474    0.58535     0.63231
## cond(log(Hist3yr))     0.63077    0.13828     0.14967
## cond(log(hrdyrsrisk))  0.41210    0.08277     0.08960
## cond(TreatmentProactive) -0.07449    0.07425     0.08074
## cond(TripletA)        -0.16582    0.17941     0.20818
## cond(TripletB)        -0.14368    0.48939     0.50582
## cond(TripletC)        -0.01814    0.56439     0.57718
## cond(TripletD)        -0.16398    0.25432     0.27730
## cond(TripletE)        -0.02395    0.32650     0.34377
## cond(TripletF)        -0.32972    0.53092     0.54565
## cond(TripletG)        -0.26334    0.54497     0.56020
## cond(TripletH)        -0.28412    0.32457     0.34555
## cond(TripletI)        -0.24516    0.21121     0.24012
##                       z value Pr(>|z|)
## cond((Int))             0.830    0.407
## cond(log(Hist3yr))      4.214  2.5e-05 ***
## cond(log(hrdyrsrisk))   4.600  4.2e-06 ***
## cond(TreatmentProactive)   0.923    0.356
## cond(TripletA)          0.797    0.426
## cond(TripletB)          0.284    0.776
## cond(TripletC)          0.031    0.975
## cond(TripletD)          0.591    0.554
```

```
## cond(TripletE)              0.070    0.944
## cond(TripletF)              0.604    0.546
## cond(TripletG)              0.470    0.638
## cond(TripletH)              0.822    0.411
## cond(TripletI)              1.021    0.307
## ---
## Signif. codes:
## 0 '***' 0.001 '**' 0.01 '*' 0.05 '.' 0.1 ' ' 1
```

```
model.avg(object = final.models2)
```

```
##
## Call:
## model.avg(object = final.models2)
##
## Component models:
## '12'     '123'    '2'      '1'      '23'     '13'
## '(Null)' '3'      '14'     '24'     '124'    '4'
## '134'    '234'    '34'     '1234'
##
## Coefficients:
##        cond((Int)) cond(log(Hist3yr)) cond(log(hrdyrsrisk))
## full     -0.5247448          0.6295046             0.4114704
## subset   -0.5247448          0.6307654             0.4121032
##        cond(TreatmentProactive) cond(TripletA)
## full                -0.01567253  -1.989004e-13
## subset              -0.07448827  -1.658243e-01
##        cond(TripletB) cond(TripletC) cond(TripletD)
## full    -1.723352e-13  -2.175951e-14  -1.966831e-13
## subset  -1.436767e-01  -1.814102e-02  -1.639757e-01
##        cond(TripletE) cond(TripletF) cond(TripletG)
## full    -2.872937e-14  -3.954925e-13  -3.158718e-13
## subset  -2.395182e-02  -3.297241e-01  -2.633439e-01
##        cond(TripletH) cond(TripletI)
## full    -3.407907e-13  -2.940621e-13
## subset  -2.841189e-01  -2.451610e-01
```

```
#Bayesian Analysis
#2006 paper
library(rstanarm)
library(bridgesampling)
rs<-stan_glm(Incidence~Treatment+A+B+C+D+E+F+G+H+I+log(Hist3yr)+log(Baseline),
             prior_intercept=normal(0,10), prior=normal(0,10),poisson,data=rbctconf
             ,
             diagnostic_file = file.path(tempdir(), "df.csv"),refresh=0)

#Marginal Likelihood
bridge<-bridge_sampler(rs)
```

```
## Iteration: 1
## Iteration: 2
## Iteration: 3
## Iteration: 4
```

```
bridge
```

```
## Bridge sampling estimate of the log marginal likelihood: -116.149
## Estimate obtained in 4 iteration(s) via method "normal".
```

```
#Most parsimonious (overdispersed) Poisson model.
#This is a negative binomial model where the variance
#is proportional to the square of the mean.
rs1B<-stan_glm.nb(Incidence~Treatment+log(hdyrsrisk)+log(Hist3yr),
                  prior_intercept=normal(0,10), prior=normal(0,10),
                  data=rbctconf,diagnostic_file =file.path(tempdir(),"df.csv"),
                     refresh=0)

summary(rs1B, probs=c(0.025,0.5,0.975), digits=3)

##
## Model Info:
##  function:     stan_glm.nb
##  family:       neg_binomial_2 [log]
##  formula:      Incidence ~ Treatment + log(hdyrsrisk) + log(Hist3yr)
##  algorithm:    sampling
##  sample:       4000 (posterior sample size)
##  priors:       see help('prior_summary')
##  observations: 20
##  predictors:   4
##
## Estimates:
##                         mean    sd     2.5%    50%    97.5%
## (Intercept)           -2.097  1.641  -5.429  -2.092   1.103
## TreatmentProactive    -0.156  0.203  -0.556  -0.154   0.236
## log(hdyrsrisk)         0.488  0.206   0.083   0.486   0.902
## log(Hist3yr)           0.891  0.361   0.189   0.889   1.598
## reciprocal_dispersion  6.298  2.189   2.778   6.020  11.385
##
## Fit Diagnostics:
##            mean    sd     2.5%    50%     97.5%
## mean_PPD  46.031  7.152  33.899  45.250  61.851
##
## The mean_ppd is the sample average posterior predictive distribution of the
   outcome variable (for details see help('summary.stanreg')).
##
## MCMC diagnostics
##                       mcse  Rhat  n_eff
## (Intercept)           0.024 1.000 4544
## TreatmentProactive    0.003 0.999 3540
## log(hdyrsrisk)        0.003 0.999 4386
## log(Hist3yr)          0.006 1.001 3488
## reciprocal_dispersion 0.039 1.001 3234
## mean_PPD              0.124 1.001 3328
## log-posterior         0.044 1.004 1682
##
## For each parameter, mcse is Monte Carlo standard error, n_eff is a crude measure
    of effective sample size, and Rhat is the potential scale reduction factor on
   split chains (at convergence Rhat=1).

#And with the herd years at risk set as an offset
#Again with the overdispersed Poisson (negative binomial) model.
rs1aB<-stan_glm.nb(Incidence~Treatment+log(hdyrsrisk)+log(Hist3yr),
                   prior_intercept=normal(0,10), prior=normal(0,10),data=rbctconf,
                   diagnostic_file =file.path(tempdir(),"df.csv"),refresh=0)

summary(rs1aB, probs=c(0.025,0.5,0.975), digits=3)
```

```
##
## Model Info:
##  function:     stan_glm.nb
##  family:       neg_binomial_2 [log]
##  formula:      Incidence ~ Treatment + log(hdyrsrisk) + log(Hist3yr)
##  algorithm:    sampling
##  sample:       4000 (posterior sample size)
##  priors:       see help('prior_summary')
##  observations: 20
##  predictors:   4
##
## Estimates:
##                         mean    sd     2.5%    50%     97.5%
## (Intercept)            -2.075   1.669  -5.281  -2.075   1.271
## TreatmentProactive     -0.157   0.213  -0.571  -0.157   0.262
## log(hdyrsrisk)          0.484   0.214   0.070   0.480   0.904
## log(Hist3yr)            0.891   0.364   0.156   0.896   1.605
## reciprocal_dispersion   6.168   2.055   2.851   5.918  10.853
##
## Fit Diagnostics:
##           mean     sd     2.5%    50%     97.5%
## mean_PPD 45.860   7.517  33.450  45.025  63.250
##
## The mean_ppd is the sample average posterior predictive distribution of the
   outcome variable (for details see help('summary.stanreg')).
##
## MCMC diagnostics
##                       mcse  Rhat  n_eff
## (Intercept)           0.025 1.000 4492
## TreatmentProactive    0.003 1.000 4142
## log(hdyrsrisk)        0.003 1.000 4106
## log(Hist3yr)          0.006 1.000 3807
## reciprocal_dispersion 0.034 1.000 3595
## mean_PPD              0.126 1.000 3562
## log-posterior         0.047 1.001 1542
##
## For each parameter, mcse is Monte Carlo standard error, n_eff is a crude measure
    of effective sample size, and Rhat is the potential scale reduction factor on
   split chains (at convergence Rhat=1).

#Upper panel Figure 4 in the text
title <- paste(" Posterior distributions")
subheader <- paste("with medians and 95% intervals")
plot(rs1B, plotfun="areas",prob=0.95, pars=c("TreatmentProactive", "log(Hist3yr)",
  "log(hdyrsrisk)"))+labs(y="Density", x="Parameter Value")+
  ggtitle(bquote(atop(bold(.(title)), atop(.(subheader), ""))))+
  theme(plot.title = element_text(hjust = 0.5))+
  theme(axis.text.y=element_blank())+
  annotate("text", x=.5, y=3.7,size=3, label= "Treatment Proactive")+
  annotate("text", x=1.5, y=2.7,size=3, label= "Log(Historical 3\n Year Incidence)"
      )+
  annotate("text", x=1.3, y=1.5,size=3, label= "Log (Herd Years at Risk)")
```

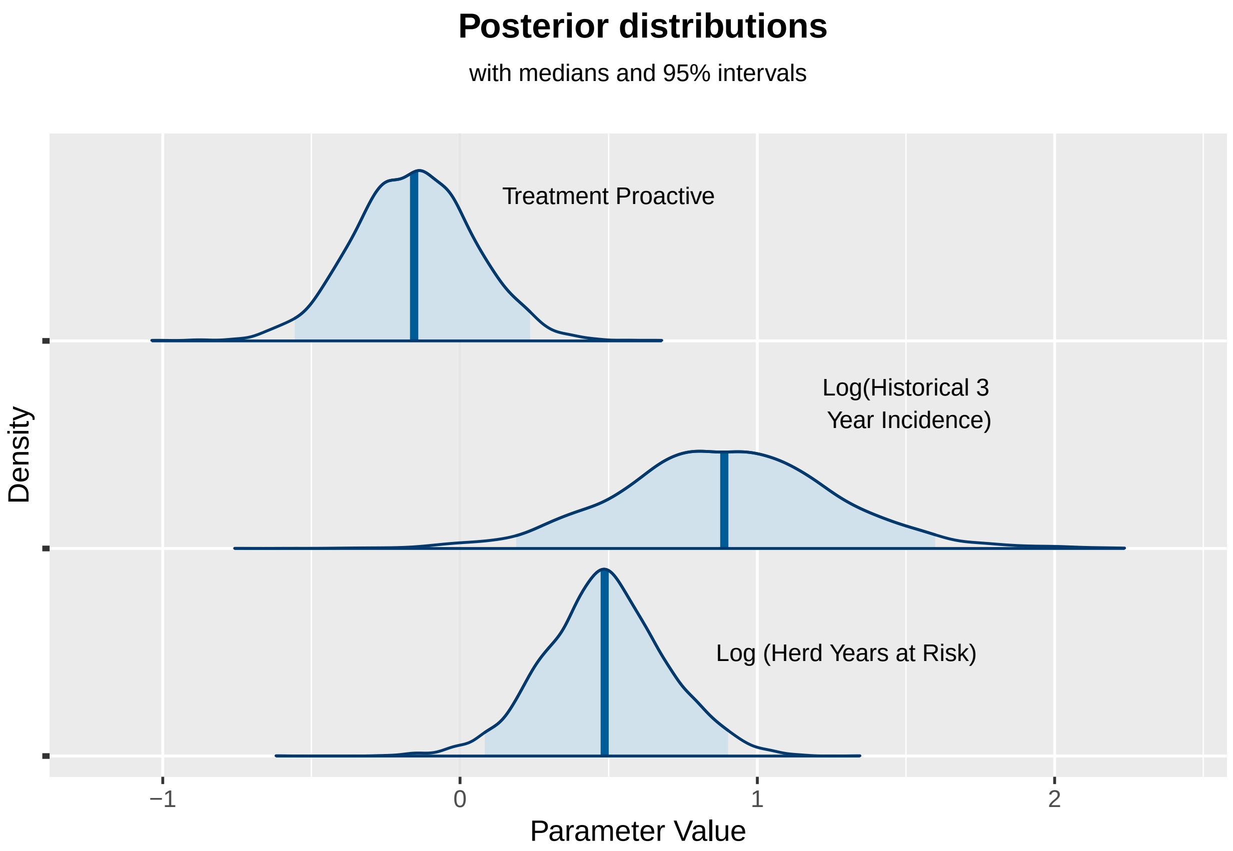


```
#Lower panel figure 4
plot(rs1aB, plotfun="areas",prob=0.95, pars=c("TreatmentProactive", "log(Hist3yr)")
   )+
  labs(y="Density", x="Parameter␣Value")+
  theme(axis.text.y=element_blank())+
  annotate("text", x=.5, y=2.7,size=3, label= "Treatment␣Proactive")+
  annotate("text", x=1.5, y=1.7,size=3, label= "Log(Historical␣3\n␣Year␣Incidence)"
     )
```

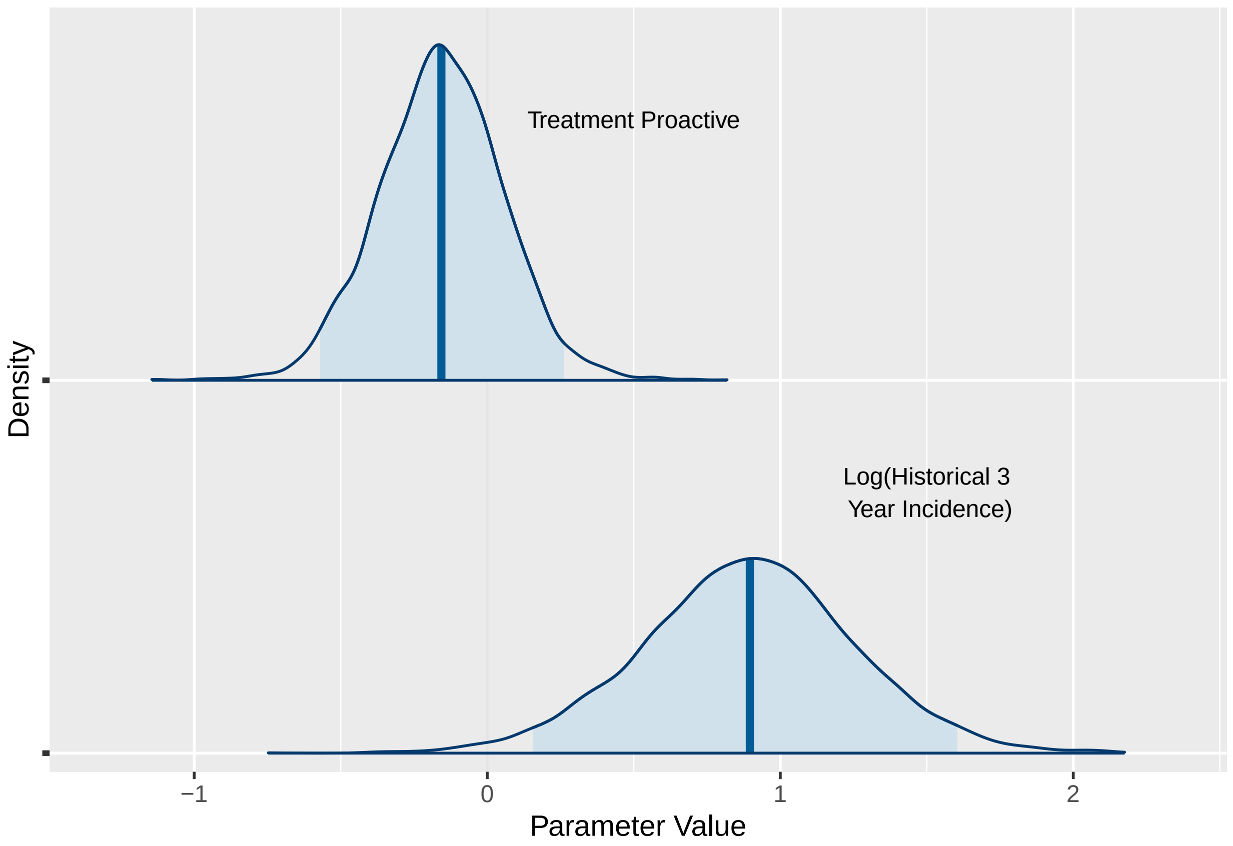


```
bridge1<-bridge_sampler(rs1B)

## Iteration: 1
## Iteration: 2
## Iteration: 3
## Iteration: 4

bridge1

## Bridge sampling estimate of the log marginal likelihood: -98.56249
## Estimate obtained in 4 iteration(s) via method "normal".

#Removal of treatment as a covariate further improves model
#support compared to the Nature model
rs2B<-stan_glm.nb(Incidence~log(hdyrsrisk)+log(Hist3yr),
                 prior_intercept=normal(0,10), prior=normal(0,10),
                 data=rbctconf,diagnostic_file = file.path(tempdir(), "df.csv"),
                    refresh=0)

#Comparison of most parsimonious (overdispersed) Poisson model
#in terms of Bayes factor

bridge2<-bridge_sampler(rs2B)

## Iteration: 1
## Iteration: 2
## Iteration: 3
## Iteration: 4
## Iteration: 5
```

```
bayes_factor(bridge2, bridge1)
```

```
## Estimated Bayes factor in favor of x1 over x2: 36.36294
```
